# Synthesis of Functionalized Hydrazines: Facile Homogeneous (N‐Heterocyclic Carbene)‐Palladium(0)‐Catalyzed Diboration and Silaboration of Azobenzenes

**DOI:** 10.1002/adsc.201601106

**Published:** 2016-11-09

**Authors:** Melvyn B. Ansell, George E. Kostakis, Holger Braunschweig, Oscar Navarro, John Spencer

**Affiliations:** ^1^ Department of ChemistryUniversity of SussexBrightonBN1 9QJU.K.; ^2^ Institut für Anorganische ChemieJulius-Maximilians-Universität WürzburgAm Hubland97074WürzburgGermany; ^3^ Institute for Sustainable Chemistry & Catalysis with BoronJulius-Maximilians-Universität WürzburgAm Hubland970704WürzburgGermany; ^4^ Division of Biomaterials and BiomechanicsDepartment of Restorative DentistrySchool of DentistryOregon Health & Science University2730 SW Moody Ave.PortlandOregon97239USA

**Keywords:** azobenzenes, diboration, N-heterocyclic carbenes, palladium, silaboration

## Abstract

The bis(N‐heterocyclic carbene)(diphenylacetylene)palladium complex [Pd(ITMe)_2_(PhC≡CPh)] (ITMe=1,3,4,5‐tetramethylimidazol‐2‐ylidene) acts as a highly active pre‐catalyst in the diboration and silaboration of azobenzenes to synthesize a series of novel functionalized hydrazines. The reactions proceed using commercially available diboranes and silaboranes under mild reaction conditions.

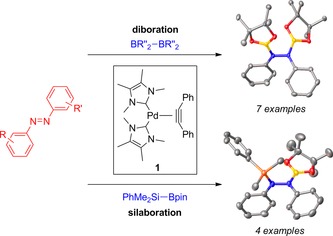

The transition metal‐catalyzed diboration (B‐B) and silaboration (Si‐B) reactions of carbon‐based unsaturated species such as alkynes,^1^ alkenes,^2^ and 1,3‐dienes,^3^ represent some of the most valuable and widely studied organic transformations in the literature. Nevertheless, the translation of this chemistry to other element‐based unsaturated bonds remains a considerable challenge. In particular, the diboration and silaboration of N=N (azo) bonds harnesses the potential for the synthesis of highly functionalized hydrazines as precursors to, for instance, polymeric materials,^4^ DNA modifiers,^5^ and glycosidase inhibitors.^6^ Despite this potential, such element‐element additions to azo moieties are extremely rare. There are only three reported isolated examples of azo diborations to yield the corresponding 1,2‐bis(boryl)hydrazines. These require the use of either an extremely reactive B–B bond in the form of azadiboriridenes,^7^ dichlorodiboranes,^8^ or a highly strained B–B bond as in [2]borametallarenophanes (Scheme 1).^9^ Recently, however, a combined computational and experimental article from Li and co‐workers showed that the diboration of N=N bonds using a commercially available and air‐stable tetraalkoxydiboron reagent such as bis(pinacolato)diboron is feasible.^10^ To the best of our knowledge there are no examples in the literature of N=N silaborations.

**Scheme 1:**
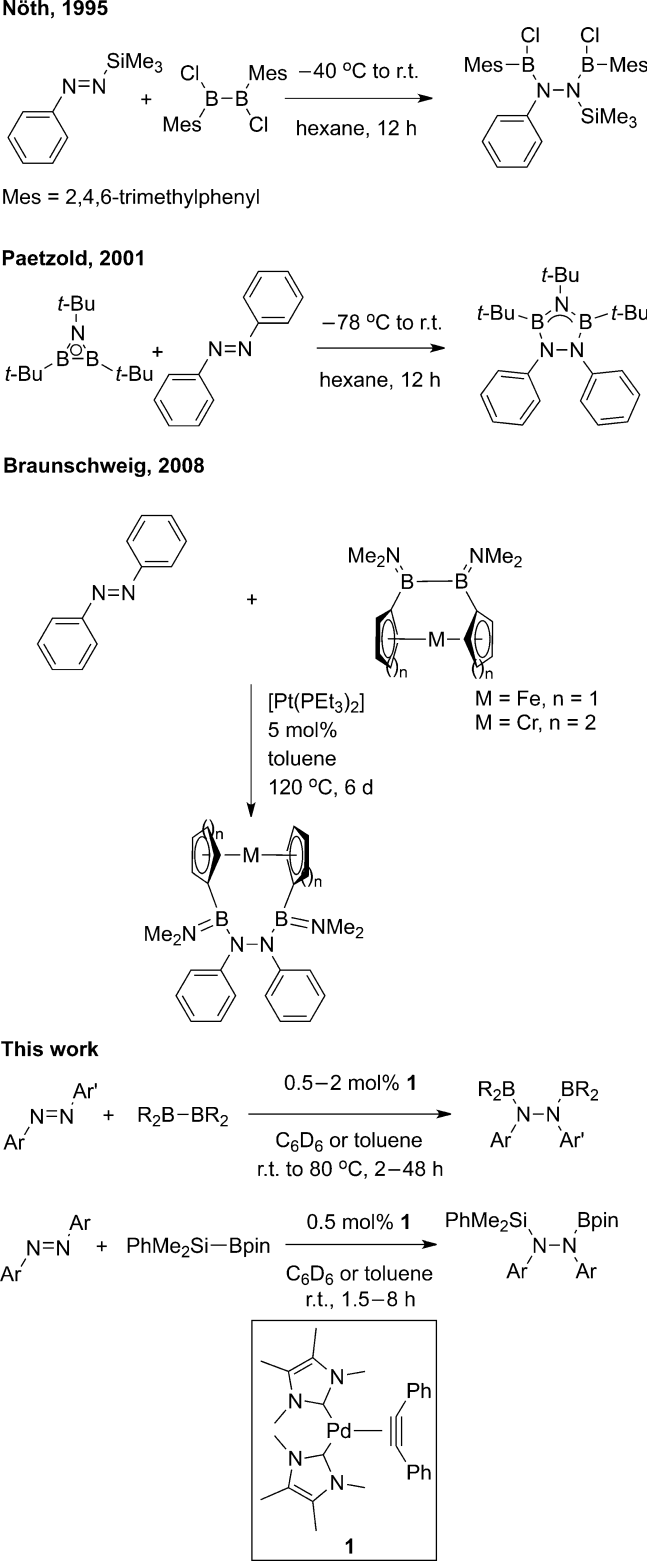
Element‐element additions to N=N bonds.

We have described the synthesis of the complex [Pd(ITMe)_2_(PhC≡CPh)] (**1**),^11^ which showed high catalytic reactivity in the regio‐ and stereoselective diboration^12^ and silaboration of alkynes.^13^ We pondered whether **1** could catalyze these element‐element additions across N=N bonds. Herein, we report the use of **1** as a very active pre‐catalyst in the diboration and silaboration of azobenzenes. The products represent the first isolated examples of 1,2‐bis(boryl)hydrazines and 1‐silyl‐2‐borylhydrazines, starting from commercially available diboranes and silaboranes, respectively.

The viability of the diboration of azobenzenes was assessed by combining, under an inert atmosphere and at room temperature, azobenzene (PhN=NPh), bis(pinacolato)diboron (B_2_pin_2_) and catalytic quantities of **1** in C_6_D_6_ in order to monitor the reaction progress by ^1^H NMR spectroscopy. The optimization of the reaction parameters resulted in 100% conversion to 1,2‐diphenyl‐1,2‐bis(4,4,5,5‐tetramethyl‐1,3,2‐dioxaborolan‐2‐yl)hydrazine (**2**) after 2 h, using as little as 0.5 mol% of **1** (Table 1).

**Table 1:** Diboration of azobenzenes.^[a]^

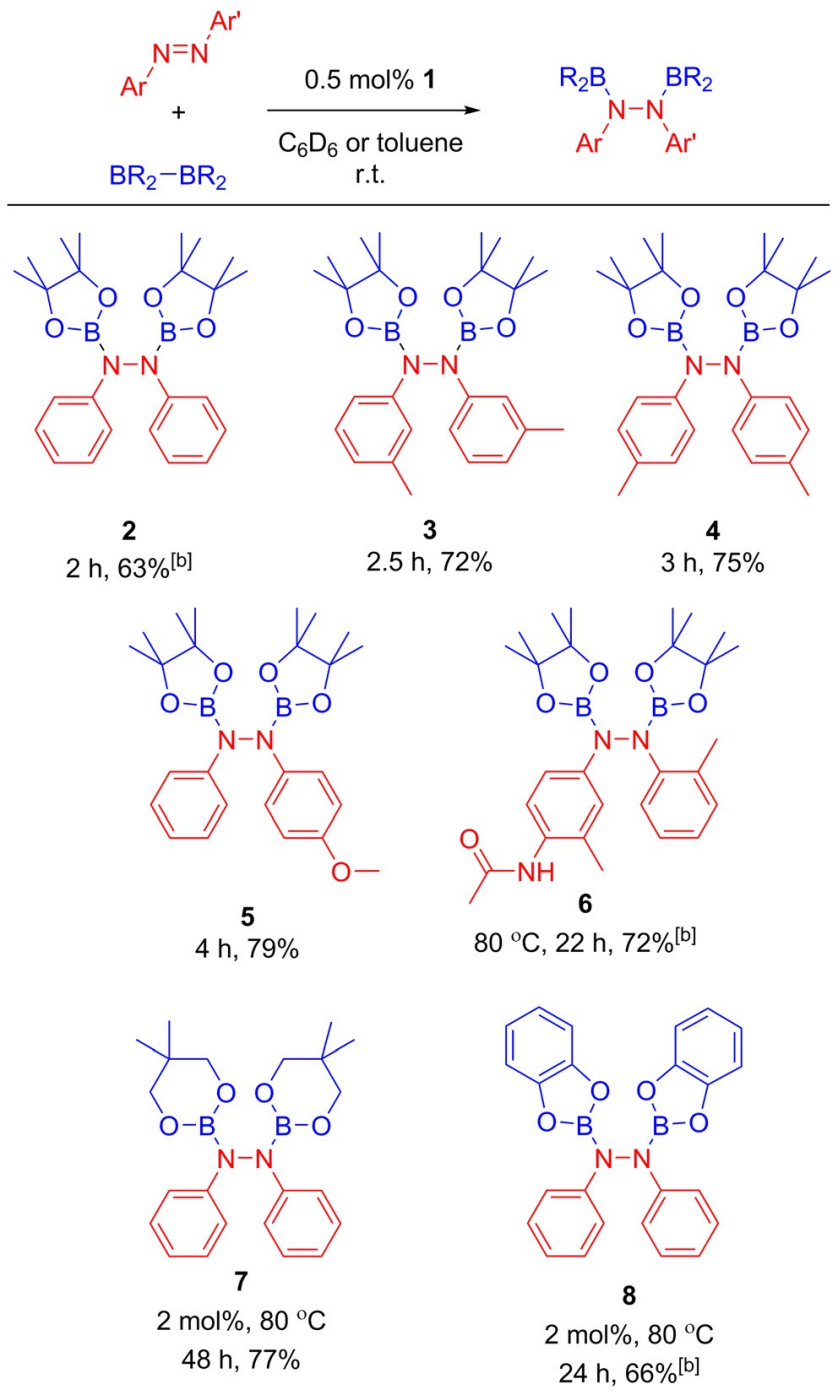

^[a]^ B_2_R_4_: 1–1.5 equiv. (see the Supporting Information for details). ^[b]^ Yields from scaled‐up reactions in toluene.

The reaction also proceeded in toluene under the same conditions on a larger scale. After several recrystallizations from hexanes, **2** was isolated as an air‐ and moisture‐sensitive white powder in 63% yield. Single crystals of **2** were isolated from a saturated hexane solution at −30 ^ο^C and the resulting X‐ray analysis is depicted in Figure 1. The crystalline structure of **2** was solved in the *P*2_1_2_1_2_1_ space group with one of the Bpin functionalities displaying a degree of dynamic disorder. A notable feature of this molecular structure is the length of the N–N bond [1.419(4) Å] which is, as expected, comparable to the N–N bond in diphenylhydrazines [1.394(7) Å],^14^ and much longer than the N=N bond of azobenzene (1.25 Å).^15^ Each N atom exhibits a distorted trigonal planar geometry [115.2(2)–128.4(6)^ο^; N1, N2: ∑=360^ο^]. The B–N bond lengths [1.410(14) Å and 1.433(4) Å] are in agreement with those of other aminoboranes of the form R_2_BNR′_2_,^9^,^15^,^16^ and imply partial double bond character.^17^ The distorted trigonal planar geometry surrounding each B atom is indicative of *sp* ^2^ hybridization [133.0(9)–127.6(9)^ο^; B1, B12: ∑=360°].

**Figure 1:**
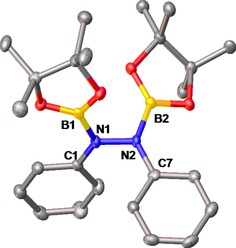
Molecular structure of **2** with thermal ellipsoids at 50% probability level. Hydrogen atoms are omitted for clarity. Selected bond lengths [Å] and angles [^ο^]: N1–N2: 1.419(4), N1–B1: 1.433(4), N2–B2: 1.410(14), C1–N1–B1: 128.1(3), N2–N1–C1: 116.2(2), N2–N1–B1: 115.2(2), C7–N2–N1: 116.1(2), B2–N2–N1: 115.5(6), B2–N2–C7: 128.4(6), O1–B1–N1: 124.6(3), O1–B1–O2: 114.3(3), O2–B1–N1: 121.3(3), O13–B2–N2: 119.4(12), O13–B2–O14: 113.0(9), O14–B2–N2: 127.6(9).

The versatility of this catalytic diboration using B_2_pin_2_ was assessed and this protocol was extended to a series of azobenzenes with a range of functionalities including alkyl, methoxy and amido moieties in the *ortho*, *meta* and *para* positions (Table 1). As with **2**, the syntheses of the novel compounds **3**–**5** only required 0.5 mol% of **1**, proceeded at room temperature and were completed in 2.5–4 h (Table 1) (see the Supporting Information for the X‐ray analysis of **4**). The synthesis of **6** required an increase of temperature (80 ^ο^C) and reaction time (22 h) to reach completion. This was attributed to the limited solubility of the azobenzene in C_6_D_6_ and in toluene. It was also possible to exchange the diboron reagent for other commercially available B–B analogues such as bis(neopentylglycolato)diboron and bis(catecolato)diboron. This resulted in the formation of **7** and **8**, respectively, albeit employing higher catalyst loadings, higher temperatures and longer reaction times than those for their B_2_pin_2_ counterpart.

We next turned our attention to the catalytic silaboration of azobenzenes. The silaborane of choice was the readily available (dimethylphenyl)silylboronic acid pinacol ester (PhMe_2_SiBpin). The reaction parameters were optimized using PhMe_2_SiBpin and azobenzene as the model substrates. To our delight, [dimethyl(phenyl)silyl]‐1,2‐diphenyl‐2‐(4,4,5,5‐tetramethyl‐1,3,2‐dioxaborolan‐2yl)hydrazine (**9**) was synthesized with 100% conversion using 0.5 mol% of **1** at room temperature in 2 h in C_6_D_6_ or toluene. Interestingly, compound **9** is air‐ and moisture‐stable which simplified purification. On stirring the crude reaction mixture in deionized H_2_O overnight, **9** was recovered as a white powder in 87% yield (Table 2). Single crystals of **9** were isolated from slow evaporation of a saturated acetone solution at room temperature. The molecular structure of **9** obtained from X‐ray analysis is shown in Figure 2. There are some noteworthy features in this molecular structure, the first was that it was solved in the P2_1_ space group. The N–N bond length [1.417(4) Å] is comparable to that of **2** [1.419(4) Å] and shorter than that of other silyl‐substituted hydrazines (e.g., Ph_2_Si{NHNH}SiPh_2_ and Ph_2_Si{NHNHMe}_2_) reported in the literature [1.421(5)–1.480(2) Å].^18^ The bonding around each N atom, as with **2**, showed a distorted trigonal planar geometry [115.3(2)–127.9(2) Å; N1, N2: ∑=360^ο^]. The B–N bond length [1.438(5) Å] is in agreement with those of other aminoboranes including **2**. Thegeometry surrounding the B‐atom is distorted trigonal planar [114.6(4)–123.8(3)^ο^; B_p_: ∑=360^ο^]. The Si–N bond length is longer [1.773(2) Å] than that of other silylamines of the form R_3_SiNR′_2_.^18^,^19^

**Table 2:** Silaboration of azobenzenes.^[a]^

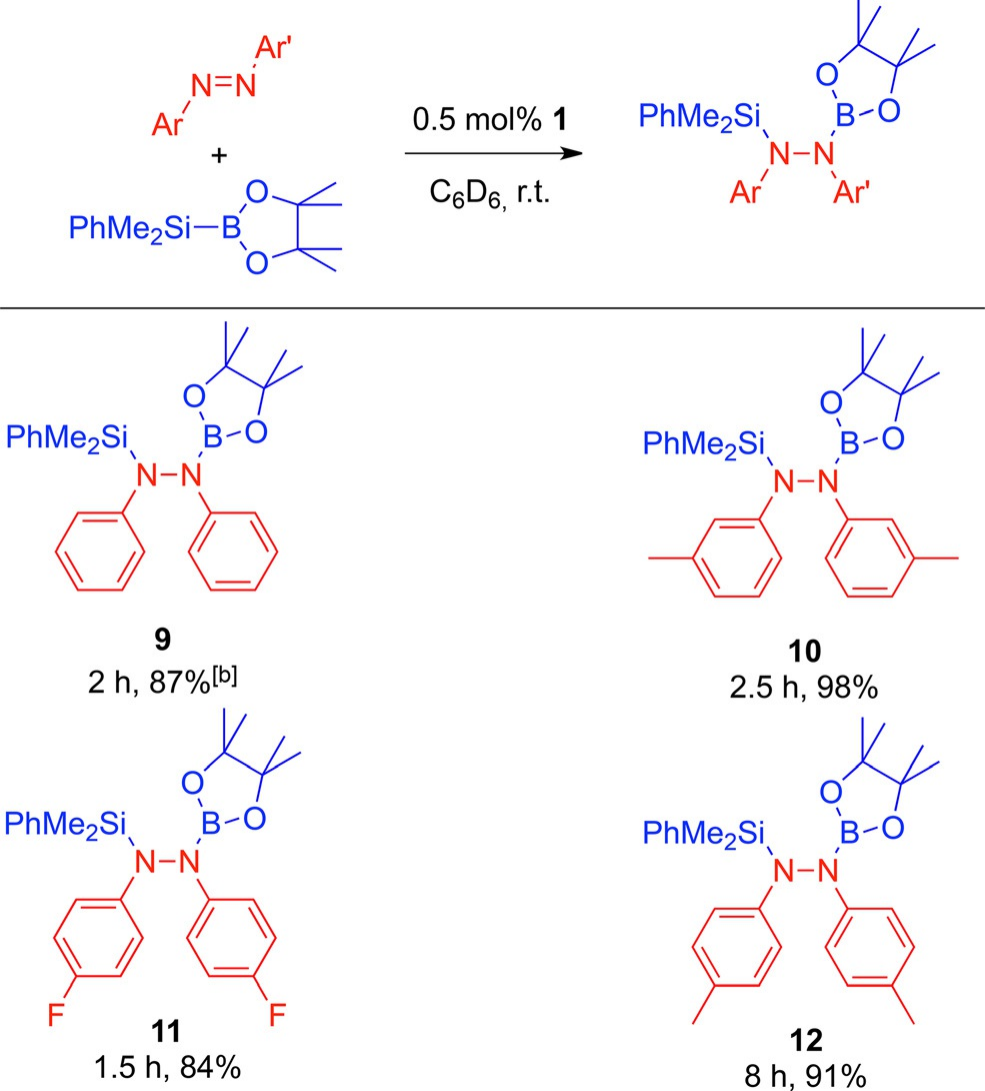

^[a]^ PhMe_2_SiBpin: 1.13–1.36 equiv. (see the Supporting Information for details). ^[b]^ Isolated yield from scaled‐up reactions in toluene. Reaction of 4‐methoxyazobenzene with PhMe_2_SiBpin gives a mixture of regioisomers (see the Supporting Information).

**Figure 2:**
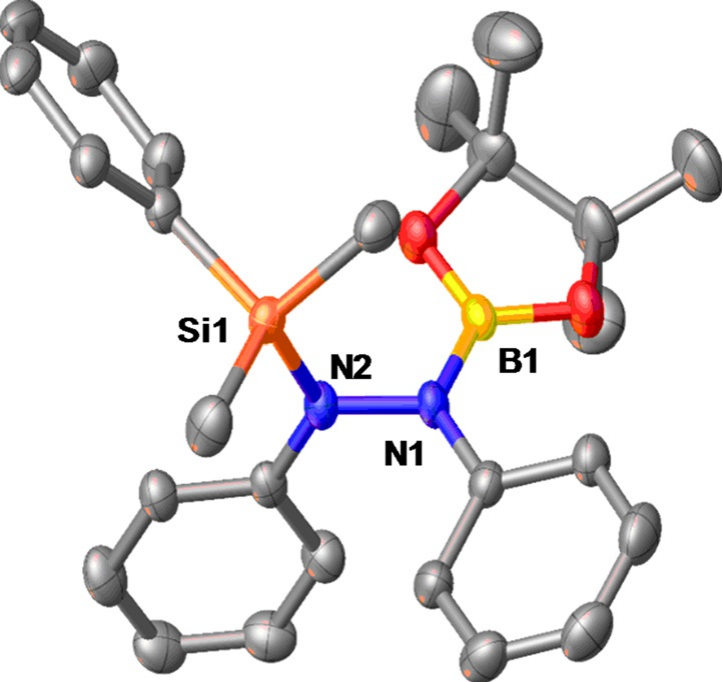
Molecular structure of **9** with thermal ellipsoids at the 50% probability level. Hydrogen atoms are omitted for clarity. Selected bond lengths [Å] and angles [^ο^]: N1–N2: 1.417(4), N1–B1: 1.438(5), Si1–N2: 1.773(3), N2–N1–C7: 115.8(3), N2–N1–B1: 116.3(3), C7–N1–B1: 127.8(3), N1–N2–Si1: 115.3(2), C6–N2–Si1: 127.9(2), C6–N2–N1: 116.3(3), O2–B1–N1: 123.8(3), O3–B1–O2: 114.6(3), O3–B1–N1: 121.6(3).

We then investigated the potential of **1** in the silaboration of other azobenzenes. This protocol was expanded to *ortho‐*, *meta‐* and *para‐*substituted symmetrical azobenzenes with alkyl and fluoro groups. The novel compounds **10**–**12** were synthesized using 0.5 mol% of **1** at room temperature, reaching completion in 1.5 to 8 h (Table 2). Compounds **10**–**12** were also stable to air and moisture (see the Supporting Information for X‐ray analysis of **11**). As expected, the application of this silaboration protocol to unsymmetrical azobenzenes resulted in a mixture of regioisomers (see the Supporting Information).

During our investigations, we found that **2** can undergo hydrolysis upon stirring in degassed deionized H_2_O overnight, affording the corresponding 1,2‐diphenylhydrazine (**13**, Scheme 2, *a*). This result, albeit accessed through palladium catalysis, supports the proposed mechanism by Li and co‐workers, whereby **2** was computationally calculated as an intermediate in the organocatalytic formation of hydrazines from their corresponding azobenzenes.^10^ Interestingly, theirreaction conditions proved to be ineffective for the hydrolysis of **9**. Instead, the cleavage of both the Si–N and B–N bonds was achieved using KO‐*t‐*Bu in a 2‐propanol/toluene mixture (Scheme 2, *b*). The cross‐coupling potential of the N–B bond in **9** was assessed, but, initial investigations, using standard reaction conditions, proved unsuccessful.^20^

**Scheme 2:**
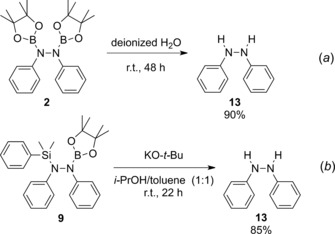
Synthesis of hydrazines from 1,2‐bis(boryl)hydrazine and 1‐silyl‐2‐borylhydrazine.

We have shown that **1** acts as a highly active pre‐catalyst in the diboration and silaboration of azobenzenes using commercially available diboranes and silaboranes, respectively. Novel 1,2‐bis(boryl)hydrazines and 1‐silyl‐2‐borylhydrazines were synthesized using low catalyst loadings, mild temperatures and short reaction times.^21^ Investigations into the reactivity potential of this novel set of compounds are ongoing in our laboratories.

## Experimental Section

### General Procedure for the Diboration of Azobenzenes

A mixture of **1** (0.5–2 mol%), azobenzene (1 equiv.) and diboron reagent (1.03–1.33 equiv.) was dissolved in deuterated benzene or toluene and stirred at room temperature or 80 °C under an N_2_ atmosphere. Upon reaching completion the reaction mixtures were cooled, filtered by cannula and the volatiles were removed under vacuum. The crude materials were purified by multiple recrystallizations in minimum volumes of hexane or hexane/toluene (3:1 or 3:2) at room temperature or −30 °C.

### General Procedure for the Silaboration of Azobenzenes

A mixture of **1** (0.5 mol%), azobenzene (1 equiv.) and (dimethylphenyl)silyl boronic acid pinacol ester (1.13–1.36 equiv.) was dissolved in deuterated benzene or toluene and stirred at room temperature under an N_2_ atmosphere. Upon completion, the volatiles were removed under vacuum, deionized water was added and the precipitated mixture was stirred overnight under ambient conditions. The products were isolated, without further purification, after filtration.

### Hydrolysis of 2

Degassed deionized water (10.0 mL) was added to **2** (32 μmol). The resulting mixture was stirred at room temperature under an argon atmosphere for 48 h. 1,2‐Diphenylhydrazine was isolated after filtration.

### Base‐Driven Alcoholysis of 9

Hydrazine **9** (0.06 mmol) and KO‐*t‐*Bu (0.12 mmol) were dissolved in 2‐PrOH/toluene (1:1, 2.0 mL) and stirred for 22 h at room temperature under an N_2_ atmosphere. The volatiles were removed under vacuum and 1,2‐diphenylhydrazine was extracted with hexane. Clean 1,2‐diphenylhydrazine was obtained by recrystallization in hexane at −30 °C.

## Supporting information

As a service to our authors and readers, this journal provides supporting information supplied by the authors. Such materials are peer reviewed and may be re‐organized for online delivery, but are not copy‐edited or typeset. Technical support issues arising from supporting information (other than missing files) should be addressed to the authors.

Supplementary
