# Supplementary material for: Synthesis of Functionalized Hydrazines: Facile Homogeneous (N‐Heterocyclic Carbene)‐Palladium(0)‐Catalyzed Diboration and Silaboration of Azobenzenes
Source: Adv Synth Catal. 2016 Nov 9;358(23):3765–9. doi: 10.1002/adsc.201601106 (PMC5215626; doi:10.1002/adsc.201601106)
Supplement: Supplementary file 1 — Supplementary [file ADSC-358-3765-s001.pdf]

Advanced Synthesis & Catalysis

Supporting Information

Supporting Information

Synthesis of Functionalized Hydrazines: Facile Homogeneous (N-Heterocyclic Carbene)-Pd(0) Catalyzed Diboration and Silaboration of Azobenzenes**

Melvyn B. Ansell, George E. Kostakis, Holger Braunschweig, Oscar Navarro,* and John Spencer**

Table of Contents

General Methods, Instrumentation and Starting Materials…………………………………..S1

Synthesis and Catalysis……………………………………………………………………...S2

Spectroscopic Data…………………………………………………………………………S12

X-ray Crystallography Data………………………………………………………………..S41

References………………………………………………………………………………….S84

General Methods, Instrumentation and Starting Materials

The manipulation of air sensitive compounds and their spectroscopic measurements were undertaken using standard Schlenk line techniques under pre-dried argon (using a BASF R2-11(G) catalyst and 4 Å molecular sieves), or in a MBraun glovebox under N_2 (O_2 < 10.0 ppm). All glassware was dried in a 160 ^{o}C oven prior to use. All solvents used for air sensitive compounds were dried by vacuum distillation followed by distillation over potassium or stored over activated 4 Å molecular sieves under an Ar atmosphere. Deuterated benzene, C_6D_6, was degassed and dried by refluxing over potassium for 3 days, vacuum transferred into ampoules and stored under N_2.

NMR spectra were recorded on a Varian VNMRS 400 (^{1}H 399.5 MHz; ^{13}C{^{1}H} 100.46 MHz; ^{29}Si{^{1}H} 79.4 MHz; ^{11}B{^{1}H} 128.2 MHz; ^{19}F 375.9 MHz). Chemical shifts are reported in ppm and are referenced to the residue solvent peak. All spectra carried out on the Varian VNMRS 400 were recorded at 303 K. Elemental analyses were carried out at the Elemental Analysis Service, London Metropolitan University.

Synthesis and Catalysis

Synthesis of 1,2-di-*p*-tolyldiazene[1]

p-Toluidine (0.331 g, 3.09 mmol), copper(I) bromide (0.013 g, 0.09 mmol) and pyridine (22.5 µl, 0.28 mmol) were dissolved in toluene (5 ml). The resulting reaction mixture was heated to 60 °C under an atmosphere of air for 3 d. At this point the reaction mixture was cooled to room temperature and filtered through a plug of silica. The resulting filtrate was concentrated and the crude mixture was purified by flash chromatography (eluent: 100% hexane). Yield: 0.110 g, 34%. ^{1}H NMR (399.5 MHz, C_6D_6): δ = 7.81 (d, $^{3}J_{HH}$ = 8.0 Hz, 4H), 7.30 (d, $^{3}J_{HH}$ = 8.0 Hz, 4H), 2.43 (s, 6H). ^{13}C{^{1}H} NMR (100.46 MHz, $CDCl_3$): 151.0, 141.3, 129.9, 122.9, 21.6.

Synthesis of 1,2-bis(4-fluorophenyl)diazene[1]

4-Fluoroaniline (94.6 µl, 1.00 mmol), copper(I) bromide (0.004 g, 0.03 mmol) and pyridine (8.9 µl, 0.11 mmol) were dissolved in toluene (4 ml). The resulting reaction mixture was heated to 60 °C for 48 h under 1 atmosphere of air. Upon cooling, the reaction mixture was filtered through a plug of silica, the filtrate volatiles were removed in vacuo and the crude solid was purified by flash chromatography (eluent: 100% hexane). Yield: 0.100 g, 92%. ^{1}H NMR (399.5 MHz, $CDCl_3$): δ = 7.92 (m, 4H), 7.20 (m, 4H). 1H NMR (399.5 MHz, C6D6): δ = 7.76 (m, 4H), 6.78 (m, 4H). ^{13}C{^{1}H} NMR (100.46 MHz, $CDCl_3$): δ = 164.6 (d, $^{1}J_{CF}$ = 251.2 Hz), 148.2 (d, $^{4}J_{CF}$ = 2.6 Hz), 125.0 (d, $^{3}J_{CF}$ = 9.0 Hz), 116.2 (d, $^{2}J_{CF}$ = 23.1 Hz). ^{19}F NMR (375.9 MHz, $CDCl_3$): δ = –109.38 (m).

Synthesis of Pd(ITMe)$_2$(PhC≡CPh) (1)

1 was synthesised following previous literature preparation. [2]

Stock solution of 1

Stock solutions were made in batches; in a glovebox 5mg of **1** was dissolved in 2 ml of C_6D_6 (9.38 µmol, 4.69 x 10^{-3} M)

Synthesis of 1,2-diphenyl-1,2-bis(4,4,5,5-tetramethyl-1,3,2-dioxaborolan-2-yl)hydrazine (2)

In C_6D_6

PhN=NPh (0.025 g, 0.14 mmol), B_2pin_2 (0.041 g, 0.16 mmol) and **1** (0.001 g, 2.62 µmol) were dissolved in C_6D_6 (0.7 ml). The resulting reaction mixture was stirred at room temperature for 2 h. At this point the reaction mixture was filtered by cannula and all volatiles were then removed in vacuo. The resulting off-white solid was recrystallized in hexane at –30 ˚C. On decanting the volatiles, the colourless crystals were dried under a high vacuum to give a white powder.

In toluene

In an ampoule, azobenzene (0.101 g, 0.55 mmol), bis(pinacalato)diboron (0.109 g, 0.43 mmol) and **1** (0.001 g, 2.06 µmol) were dissolved in toluene (2 ml). The resulting reaction mixture was stirred at ambient temperature under an N_2 atmosphere for 2 h. At this stage the reaction mixture was filtered via cannula and the filtrates volatiles were removed in vacuo. The resulting crude solid was re-crystallized in hexane (3 x 5 ml), which resulted in isolation of an off white powder. Yield: 0.119 g, 63%. ^{1}H NMR (399.5 MHz, C_6D_6): δ = 7.76 (m, 4H, Ph), 7.16 (m, 4H, Ph), 6.81 (m, 2H, *p*-Ph), 1.12 (s, 12H, Bpin), 1.08 (s, 12H, Bpin). ^{13}C{^{1}H} NMR (100.46 MHz, C_6D_6): δ = 146.5 (*i*-Ph), 129.2 (Ph), 121.6 (*p*-Ph), 117.0 (Ph), 83.5 (**C**, Bpin), 24.8 (**C**H_3, Bpin), 24.4 (**C**H_3, Bpin). ^{11}B{^{1}H} NMR (128.2 MHz, C_6D_6): δ = 25.84. Elem. Anal. Calcd for $C_{24}H_{34}O_4N_2B_2$: C, 66.09%; H, 7.86%; N, 6.42%. Found: C, 66.41%; H, 7.56%; N, 6.62%.

Synthesis of 1,2-bis(4,4,5,5-tetramethyl-1,3,2-dioxaborolan-2-yl)-1,2-di-m-tolylhydrazine (3)

3,3'-dimethylazobenzene (0.025 g, 0.12 mmol), B_2pin_2 (0.041 g, 0.16 mmol) and **1** (0.32 mg. 0.59 µmol) were dissolved in C_6D_6 (0.7 ml). The reaction mixture was stirred at room temperature under a nitrogen atmosphere for 2.5 h. At this point the volatiles were removed in vacuo. The resulting off-white solid was recrystallized in toluene/hexane (1:3, 5.0 ml) and then hexane (2 x 2.0 ml) at –30 °C. Yield: 0.040 g, 72%. ^{1}H NMR (399.5 MHz, C_6D_6): δ = 7.63 (m, 2H, 6-Ph), 7.63 (s, 2H, 2-Ph), 7.13 (m, 2H, 5-Ph), 6.68 (d, $^3J_{HH}$ = 7.4 Hz, 2H, 4-Ph), 2.11 (s, 6H, Me), 1.14 (s, 12H, Bpin), 1.11 (s, 12H, Bpin). ^{13}C{^{1}H} NMR (100.46 MHz, C_6D_6): δ = 146.7 (1-Ph), 138.6 (3-Ph), 129.1 (5-Ph), 122.5 (4-Ph), 117.7 (2-Ph), 114.5 (6-Ph), 83.5 (**C**, Bpin), 24.9 (CH_3, Bpin), 24.4 (CH_3, Bpin), 21.9 (Me). ^{11}B{^{1}H} NMR (128.2 MHz, C_6D_6): δ = 25.54. Elem. Anal. Calcd for $C_{26}H_{38}O_4N_2B_2$: C, 67.27%; H, 8.25%; N, 6.03%. Found: C, 67.33%; H, 8.27%; N, 6.10%.

Synthesis of 1,2-bis(4,4,5,5-tetramethyl-1,3,2-dioxaborolan-2-yl)-1,2-di-p-tolylhydrazine (4)

1,2-di-*p*-Tolyldiazene (0.025 g, 0.11 mmol), B_2pin_2 (0.033 g, 0.13 mmol) and **1** (0.30 mg, 0.56 µmol) were dissolved in C_6D_6 (0.7 ml). The resulting reaction mixture was stirred at room temperature under a N_2 atmosphere for 3 h. At this point, the volatiles were removed *in vacuo* and the crude reaction mixture was recrystallized in hexane (3 x 2 ml) at –30 °C resulting in the isolation of a white powder. Yield: 0.040 g, 75%. ^{1}H NMR (399.5 MHz, C_6D_6, 400 MHz): δ = 7.69 (m, 4H, *o*-PhMe), 7.00 (m, 4H, *m*-PhMe), 2.07 (s, 6H, Ph**Me**), 1.15 (s, 12H, Bpin), 1.11 (s, 12H, Bpin). ^{13}C{^{1}H} NMR (100.46 MHz, C_6D_6): δ = 144.2 (*i*-PhMe), 130.4 (*p*-PhMe), 129.7 (*m*-PhMe), 117.3 (*o*-PhMe), 83.4 (**C**, Bpin), 24.9 (**C**H_3, Bpin), 24.5 (**C**H_3, Bpin), 20.6

(Ph**Me**). $^{11}B\{^1H\}$ NMR (128.2 MHz, C_6D_6): δ = 25.53. Elem. Anal. Calcd for $C_{26}H_{38}O_4N_2B_2$: C, 67.27%; H, 8.25%; N, 6.03%. Found: C, 67.19%; H, 8.27%; N, 6.12%.

Synthesis of 1-(4-methoxyphenyl)-2-phenyl-1,2-bis(4,4,5,5-tetramethyl-1,3,2-dioxaborolan-2-yl)hydrazine (5)

1-(4-Methoxyphenyl)-2-phenyldiazene (0.099 g, 0.46 mmol), B_2pin_2 (0.144 g, 0.61 mmol) and **1** (0.001 g, 0.24 µmol) were dissolved in C_6D_6 (0.7 ml). The resulting reaction mixture was stirred at room temperature under a N_2 atmosphere for 4 h. At this stage the sample was filtered via a cannula, the filtrate volatiles were removed in vacuo and the resulting off-white solid was recrystallized in hexane (3 x 4 ml). Yield: 0.170 g, 79%. ^1H NMR (399.5 MHz, C_6D_6): δ = 7.77 (m, 2H, *m*-Ph), 7.62 (m, 2H, **Ph**OMe), 7.18 (m, 2H, *o*-Ph), 6.83 (m, 1H, *p*-Ph), 6.75 (m, 2H, **Ph**OMe), 3.28 (s, 3H, OMe), 1.15 (s, 6H, Bpin), 1.14 (s, 6H, Bpin), 1.11 (s, 6H, Bpin), 1.10 (s, 6H, Bpin). $^{13}C\{^1H\}$ NMR (100.46 MHz, C_6D_6): δ = 155.2 (*p*-PhOMe), 146.7 (*i*-Ph), 139.7 (*i*-PhOMe), 129.1 (*o*-Ph), 121.5 (*p*-Ph), 118.6 (**Ph**OMe), 117.2 (*m*-Ph), 114.6 (**Ph**OMe), 83.5 (**C**, Bpin), 55.0 (OMe), 24.9 (**C**H3, Bpin), 24.5 (**C**H3, Bpin). $^{11}B\{^1H\}$ NMR (128.2 MHz, C_6D_6): δ = 25.30. Elem. Anal. Calcd for $C_{25}H_{36}O_5N_2B_2$: C, 64.41%; H, 7.78%; N, 6.01%. Found: C, 64.28%; H, 7.65%; N, 6.09%.

Synthesis of N-(4-(1,2-bis(4,4,5,5-tetramethyl-1,3,2-dioxaborolan-2-yl)-2-(o-tolyl)hydrazinyl)-2-methylphenyl)acetamide (6)

4-Acetamido-2’,3-dimethylazobenzene (0.120 g, 0.45 mmol), B_2pin_2 (0.144 g, 0.57 mmol) and **1** (0.001 g, 2.25 µmol) were dissolved in toluene (1.5 ml). The resulting reaction mixture was heated to 80 °C under a N_2 atmosphere for 22 h. At this point, the reaction mixture was cooled to room temperature, dissolved in dioxane (15 ml) and filtered via cannula. The filtrate volatiles were removed in vacuo. The off white solid was recrystallized in toluene/hexane (5:1, 2 x 5

ml) at −30 °C and then washed with hexane (5 ml). Yield: 0.169 g, 72%. ^{1}H NMR (399.5 MHz, C_6D_6): δ = 7.92 (d, $^3J_{HH}$ = 8.7 Hz, 1H, [1]5-PhH), 7.74 (m, 1H, [2]4-PhH), 7.63 (d, $^3J_{HH}$ = 8.7 Hz, [1]6-PhH), 7.47 (s, 1H, [1]2-PhH), 7.06 (m, 2H, [2]3-PhH/[2]5-PhH), 6.88 (m, 1H, [2]6-PhH), 5.84 (s, 1H, C(O)N**H**), 2.63 (s, 3H, [2]2-Ph**Me**), 1.83 (s, 3H, [1]3-Ph**Me**), 1.51 (s, 3H, **Me**C(O)NH-), 1.15 (s, 12H, Bpin), 1.13 (s, 12H, Bpin). ^{13}C{^{1}H} NMR (100.46 MHz, C_6D_6): δ = 166.7 (**C**(O)NH), 144.9 ([2]1-Ph), 143.8 ([1]1-Ph), 132.9 ([2]2-Ph), 131.5, 131.4 ([2]3-Ph), 129.8, 126.6 ([2]5-Ph), 125.0 ([2]6-Ph), 124.7 ([2]4-Ph), 124.2 ([1]5-Ph), 122.0 ([1]2-Ph), 118.6 ([1]6-Ph), 83.5 (**C**, Bpin), 25.1 (**C**H_3, Bpin), 25.1 (**C**H_3, Bpin), 24.6 (**C**H_3, Bpin), 24.4 (Bpin), 23.6 (**Me**C(O)NH), 19.8 ([2]2-Ph**Me**), 18.1 ([1]3-Ph**Me**). ^{11}B{^{1}H} (128.2 MHz, C_6D_6): δ = 25.22. Elem. Anal. Calcd for $C_{28}H_{41}O_5N_3B_2$: C, 64.52%; H, 7.93%; N, 8.06%. Found: C, 64.66%; H, 8.07%; N, 8.21%.

([1] = ring 1 and [2] = ring 2, see Spectroscopic Data)

Synthesis of 1,2-bis(5,5-dimethyl-1,3,2-dioxaborinan-2-yl)-1,2-diphenylhydrazine (7)

Azobenzene (0.025 g, 0.14 mmol), 5,5,5’,5’-tetramethyl-2,2’-bi(1,3,2-dioxaborinane) (0.035 g, 0.15 mmol) and **1** (0.001 g, 2.63 µmol) were dissolved in C_6D_6 (0.7 ml). The resulting reaction mixture was heated to 80 °C under an N_2 atmosphere for 48 h. At this stage, the reaction mixture was cooled to room temperature, the volatiles were removed in vacuo. The resulting brown oily solid was recrystallized in toluene/hexane (2:3, 2 ml) and then hexane (2 x 2 ml) at −30 °C. A colourless crystalline solid was obtained as a result. Yield: 0.043 g, 77%. ^{1}H NMR (399.5 MHz, C_6D_6): δ = 7.83 (d, $^3J_{HH}$ = 8.1 Hz, 4 H, Ph), 7.23 (m, 4H, Ph), 6.86 (pseudo t, $^3J_{HH}$ = 7.3 Hz, 2H, *p*-Ph), 3.37 (m, 8H, CH_2), 0.63 (s, 12H, CH_3). ^{13}C{^{1}H} NMR (100.46 MHz, C_6D_6): δ = 147.1 (*i*-Ph), 128.5 (Ph), 120.8 (*p*-Ph), 118.0 (Ph), 72.2 (**C**H_2), 31.4 (**C**{CH_3}$_2$), 21.1 (CH_3). ^{11}B{^{1}H} NMR (128.2 MHz, C_6D_6): δ = 21.32. Elem. Anal. Calcd for $C_{22}H_{30}O_4N_2B_2$: C, 64.75%; H, 7.41%; N, 6.86%. Found: C, 64.66%; H, 7.44%; N, 6.90%.

Synthesis of 1,2-bis(benzo[d][1,3,2]dioxaborol-2-yl)-1,2-diphenylhydrazine (8)

In C_6D_6

Azobenzene (0.025 g, 0.14 mmol), 2,2'-bibenzo[d][1,3,2]dioxaborole (0.035 g, 0.15 mmol) and **1** (0.001 g, 2.63 µmol) were dissolved in C_6D_6 (0.7 ml). The resulting reaction mixture was heated at 80 °C under a N_2 atmosphere for 24 h. At this stage the volatiles were removed in vacuo, the crude brown oily solid was then recrystallized in toluene/hexane (1:3, 2 ml) and then toluene (1 x 5 ml) at –30 °C. This resulted in the isolation of an off-white powdered solid.

In toluene

Azobenzene (0.060 g, 0.32 mmol), 2,2'-bibenzo[d][1,3,2]dioxaborole (0.078 g, 0.33 mmol) and 1 (0.003 g, 6.38 µmol) were dissolved in toluene (1.5 ml). The resulting reaction mixture was heated to 80 °C and stirred at this temperature under a N_2 atmosphere for 24 h. The volatiles were removed in vacuo and the crude oily solid was recrystallized in toluene (3 x 3 ml) at –30 °C. The resulting off-white powder was washed with hexane (3 x 3 ml). Yield: 0.089 g, 66%. ^{1}H NMR (399.5 MHz, C_6D_6): δ = 7.70 (d, $^3J_{HH}$ = 8.3 Hz, 4H, N{*o*-Ph}), 7.13 (m, 4H, N{*m*-Ph}), 6.87 (dd, *J* = 8.3 Hz, 6.6 Hz, 2H, N{*p*-Ph}), 6.81 (m, 4H, cat-3-Ph), 6.64 (m, 4H, cat-2-Ph). ^{13}C{^{1}H} NMR (100.46 MHz, C_6D_6): δ = 148.8 (cat-1-Ph), 144.3 (N{*i*-Ph}), 129.7 (N{*m*-Ph}), 123.6 (N{*p*-Ph}), 122.7 (cat-2-Ph), 117.7 (N{*o*-Ph}), 112.5 (cat-3-Ph). ^{11}B{^{1}H} (128.2 MHz, C_6D_6): δ = 26.65. Elem. Anal. Calcd for $C_{24}H_{18}O_4N_2B_2$: C, 68.63%; H, 4.32%; N, 6.67%. Found: C, 68.63%; H, 4.38%; N, 6.59%.

Synthesis of 1-(dimethyl(phenyl)silyl)-1,2-diphenyl-2-(4,4,5,5-tetramethyl-1,3,2-dioxaborolan-2-yl)hydrazine (9)

In C_6D_6

Azobenzene (0.025 g, 0.14 mmol), $PhMe_2SiBpin$ (46.0 µl, 0.17 mmol) and **1** (0.36 mg, 0.68 µmol) were dissolved in C_6D_6 (0.7 ml). The resulting reaction mixture was stirred at room

temperature for 2 h. At this point the volatiles were removed in vacuo and the resulting oily solid was washed with cold hexane (4 x 1 ml). Upon drying the resulting white powder was obtained without further purification.

In toluene

Azobenzene (0.070 g, 0.38 mmol), $PhMe_2SiBpin$ (0.120 g, 0.46 mmol) and **1** (0.001 g, 1.31 μmol) were dissolved in toluene (1.5 ml). The reaction mixture was stirred at room temperature under a N_2 atmosphere for 2 h. At this stage the volatiles were removed in vacuo, the resulting oily solid was stirred in deionized H_2O overnight and a white powder was obtained upon filtering. Yield: 0.148 g, 87%. ^1H NMR (399.5 MHz, C_6D_6): δ = 7.85 (m, 2H, $SiMe_2$**Ph**), 7.68 (m, 2H, *o*-Ph{1}), 7.23 (m, 3H, $SiMe_2$**Ph**), 7.16 (m, 2H, *m*-Ph{1}), 7.03 (m, 4H, *o*- and *m*-Ph{2}), 6.85 (m, 1H, *p*-Ph{1}), 6.68 (m, 1H, *p*-Ph{2}), 1.05 (s, 6H, Bpin), 1.01 (s, 6H, Bpin), 0.61 (s, 3H, Si**Me_2**Ph), 0.58 (s, 3H, Si**Me_2**Ph). $^{13}C\{^1H\}$ NMR (100.46 MHz, C_6D_6): δ = 149.8 (*i*-Ph{2}), 147.5 (*i*-Ph{1}), 138.3 ($SiMe_2$***i*-Ph**), 134.8 ($SiMe_2$**Ph**), 129.7 ($SiMe_2$*p*-**Ph**), 129.3 (Ph{2}), 129.1 (*m*-Ph{1}), 128.1 ($SiMe_2$**Ph**), 121.9 (*p*-Ph{1}), 119.1 (p-Ph{2}), 118.0 (*o*-Ph{1}), 114.1 (Ph{2}), 83.6 (**C**, Bpin), 24.8 (**C**H_3, Bpin), 24.4 (**C**H_3, Bpin), –0.7 (Si**Me_2**Ph), –0.8 (Si**Me_2**Ph). $^{11}B\{^1H\}$ NMR (128.2 MHz, C_6D_6): δ = 25.78. $^{29}Si\{^1H\}$ NMR (79.4 MHz, C_6D_6): δ = 4.59. Elem. Anal. Calcd for $C_{26}H_{33}O_2N_2SiB$: C, 70.26%; H, 7.48%; N, 6.30%. Found: C, 70.18%; H, 7.50%; N, 6.30%.

Synthesis of 1-(dimethyl(phenyl)silyl)-2-(4,4,5,5-tetramethyl-1,3,2-dioxaborolan-2-yl)-1,2-di-m-tolylhydrazine (10)

1,2-di-*m*-Tolyldiazene (0.024 g, 0.11 mmol), dimethyl(phenyl)(4,4,5,5-tetramethyl-1,3,2-dioxaborolan-2-yl)silane (0.040 g, 0.15 mmol) and **1** (0.30 mg, 0.56 μmol) were dissolved in C_6D_6 (0.7 ml). The resulting reaction mixture was stirred at room temperature under a N_2 atmosphere. After 2.5 h, the volatiles were removed in vacuo, deionized H_2O (100 ml) was

added and the crude reaction mixture was stirred at room temperature for 15 h. The product was obtained cleanly as a white powder on filtering the precipitate. Yield: 0.052 g, 98%. ^{1}H NMR (399.5 MHz, C_6D_6): δ = 7.88 (m, 2H, $SiMe_2$*o*-Ph), 7.62 (dd, *J* = 8.3, 2.2 Hz, 1H, [2]Ph(6)), 7.55 (s, 1H, [2]Ph(2)), 7.24 (m, 3H, $SiMe_2$*m*/*p*-Ph), 7.15 (m, 1H, [2]Ph(5)), 6.96 (m, 3H, [1]Ph(2/5/6)), 6.71 (m, 1H, [2]Ph(4)), 6.53 (m, 1H, [1]Ph(4)), 2.13 (s, 3H, [2]Me), 2.01 (s, 3H, [1]Me), 1.08 (s, 6H, Bpin), 1.04 (s, 6H, Bpin), 0.67 (s, 3H, $SiMe_2$Ph), 0.62 (s, 3H, $SiMe_2$Ph). ^{13}C{^{1}H} NMR (100.46 MHz, C_6D_6): δ = 149.9 ([1]Ph(1)), 147.6 ([2]Ph(1)), 138.7 ([1]Ph(3)), 138.6 ([2]Ph(3)), 138.5 ($SiMe_2$*i*-Ph), 134.9 ($SiMe_2$*o*-Ph), 129.7 ($SiMe_2$*p*-Ph), 129.3, 129.1 ([2]Ph(5)), 128.1 ($SiMe_2$*m*-Ph), 122.7 ([2]Ph(4)), 120.1 ([1]Ph(4)), 118.4 ([2]Ph(2)), 115.3 ([2]Ph(6)), 114.6, 111.5, 83.5 (**C**, Bpin), 24.8 (**C**H_3, Bpin), 24.4 (**C**H_3, Bpin), 21.8 ([1]Me), 21.8 ([2]Me), –0.6 ($SiMe_2$Ph), –0.9 ($SiMe_2$Ph). ^{11}B{^{1}H} NMR (128.2 MHz, C_6D_6): δ = 25.84. ^{29}Si{^{1}H} NMR (79.4 MHz, C_6D_6): δ = 4.40. Elem. Anal. Calcd for $C_{28}H_{37}N_2O_2SiB$: C, 71.17%; H, 7.89%; N, 5.93%. Found: C, 71.06%; H, 7.74%; N, 5.75%.

Synthesis of 1-(dimethyl(phenyl)silyl)-1,2-bis(4-fluorophenyl)-2-(4,4,5,5-tetramethyl-1,3,2-dioxaborolan-2-yl)hydrazine (11)

1,2-bis(4-Fluorophenyl)diazene (0.027 g, 0.12 mmol), $PhMe_2SiBpin$ (0.038 g, 0.14 mmol) and **1** (0.33 mg, 0.62 µmol) were dissolved in C_6D_6 (0.7 ml). The resulting reaction mixture was stirred at room temperature for 1.5 h under a N_2 atmosphere. At this stage the volatiles were removed in vacuo, deionized H_2O (100 ml) was added and the reaction mixture was stirred for a further 24 h at room temperature. The H_2O was then decanted and the off white solid was dried under a high vacuum. Yield: 0.050 g, 84%. ^{1}H NMR (399.5 MHz, C_6D_6): δ = 7.68 (m, 2H, $SiMe_2$*o*-Ph), 7.37 (m, 2H, [2]*o*-PhF), 7.17 (m, 3H, $SiMe_2$*m*,*p*-Ph), 6.74 (m, 2H, [1]*o*-PhF), 6.74 (m, 2H, [2]*m*-PhF), 6.60 (m, 2H, [1]*m*-PhF), 0.97 (s, 6H, Bpin), 0.92 (s, 6H, Bpin), 0.48 (s, 3H, Si**Me$_2$**Ph), 0.43 (s, 3H, Si**Me$_2$**Ph). ^{13}C{^{1}H} NMR (100.46 MHz, C_6D_6): δ = 159.8 (d,

$^{1}J_{CF}$ = 239.7 Hz, [2]*p*-PhF), 158.3 (d, $^{1}J_{CF}$ = 236.7 Hz, [1]*p*-PhF), 145.6 ([1]*i*-PhF), 143.3 ([2]*i*-PhF), 137.8 (SiMe$_2$***i*-Ph**), 134.6 (SiMe$_2$*o*-Ph), 130.0 (SiMe$_2$*p*-Ph), 128.2 (SiMe$_2$*m*-Ph), 119.1 (d, $^{3}J_{CF}$ = 7.6 Hz, [2]*o*-PhF), 115.8 (d, $^{2}J_{CF}$ = 21.9 Hz, [2]*m*-PhF), 115.6 (d, $^{2}J_{CF}$ = 21.9 Hz, [1]m-PhF), 114.7 (d, $^{3}J_{CF}$ = 7.6 Hz, [1]*o*-PhF), 83.7 (**C**, Bpin), 24.8 (**C**H_3, Bpin), 24.3 (**C**H_3, Bpin), –0.8 (Si**Me$_2$**Ph), –1.1 (Si**Me$_2$**Ph). ^{11}B{^{1}H} NMR (128.2 MHz, C_6D_6): δ = 25.35. ^{19}F{^{1}H} NMR (375.9 MHz, C_6D_6): δ = -122.60 (m), -126.70 (m). ^{29}Si{^{1}H} NMR (79.4 MHz, C_6D_6): δ = 4.85. Elem. Anal. Calcd for $C_{26}H_{31}O_2N_2F_2SiB$: C, 65.00%; H, 6.50%; N, 5.83%. Found: C, 64.81%; H, 6.39%; N, 6.00%.

Synthesis of 1-(dimethyl(phenyl)silyl)-2-(4,4,5,5-tetramethyl-1,3,2-dioxaborolan-2-yl)-1,2-di-p-tolylhydrazine (12)

1,2-di-*p*-Tolyldiazene (0.031 g, 0.15 mmol), dimethyl(phenyl)(4,4,5,5-tetramethyl-1,3,2-dioxaborolan-2-yl)silane (45.0 µl, 0.17 mmol) and **1** (0.40 mg, 0.74 µmmol) were dissolved in C_6D_6 (0.7 ml). The resulting reaction was stirred at room temperature under a N_2 atmosphere for 8 h. At this stage the volatiles were removed in vacuo, deionized H_2O (80 ml) was added and the precipitated mixture was stirred at room temperature for 16h. The H_2O was decanted resulting in isolation of a white powder. Yield: 0.069 g, 91%. ^{1}H NMR (399.5 MHz, C_6D_6): δ = 7.90 (m, 2H, SiMe$_2$*o*-Ph), 7.64 (m, 2H, [2]*o*-PhMe), 7.26 (m, 3H, SiMe$_2$*m,p*-Ph), 7.00 (m, 4H, [1]*o*-PhMe and [2]*m*-PhMe), 6.82 (m, 2H, [1]*m*-PhMe), 2.10 (s, 3H, [2]Ph**Me**), 2.04 (s, 3H, [1]Ph**Me**), 1.07 (s, 6H, Bpin), 1.03 (s, 6H, Bpin), 0.65 (s, 3H, Si**Me$_2$**Ph), 0.62 (s, 3H, Si**Me$_2$**Ph). ^{13}C{^{1}H} NMR (100.46 MHz, C_6D_6): δ = 147.5 ([1]*i*-PhMe), 145.2 ([2]*i*-PhMe), 138.7 (SiMe$_2$*i*-Ph), 134.8 (SiMe$_2$*o*-Ph), 130.8 ([2]*p*-PhMe), 129.9 ([1]*m*-PhMe), 129.7 ([2]*m*-PhMe), 129.7 ([1]SiMe$_2$*p*-Ph), 127.9 (SiMe$_2$*m*-Ph), 127.7 ([1]*p*-PhMe), 118.1 ([2]*o*-PhMe), 114.1 ([1]*o*-PhMe), 83.4 (**C**, Bpin), 24.8 (**C**H_3, Bpin), 24.4 (**C**H_3, Bpin), 20.6 ([1]Ph**Me**), 20.4 ([2]Ph**Me**), –0.6 (Si**Me$_2$**Ph), –0.8 (Si**Me$_2$**Ph). ^{11}B{^{1}H} NMR (128.2 MHz, C_6D_6): δ = 25.81.

$^{29}Si\{^1H\}$ NMR (79.4 MHz, C_6D_6): δ = 4.10. Elem. Anal. Calcd for $C_{28}H_{37}O_2N_2SiB$: C, 71.17%; H, 7.89%; N, 5.93%. Found: C, 70.99%; H, 8.02%; N, 5.90%.

Reaction of 1-(4-methoxyphenyl)-2-phenyldiazene and $PhMe_2SiBpin$

1-(4-Methoxyphenyl)-2-phenyldiazene (0.025 g, 0.12 mmol), dimethyl(phenyl)(4,4,5,5-tetramethyl-1,3,2-dioxaborolan-2-yl)silane (35.0 µl, 0.13 mmol) and **1** (0.31 mg, 0.59 µmol) were dissolved in C_6D_6 (0.7 ml). The resulting reaction mixture was stirred at room temperature for 24 h under a N_2 atmosphere. Crude NMR analysis at this stage showed a mixture of inseparable regioisomers (see ^1H NMR spectrum below).

Hydrolysis of 2 to form 1,2-diphenylhydrazine

To **2** (0.014 g, 31.49 µmol) degassed deionized H_2O (10 ml) was added. The resulting reaction mixture was stirred for 48 h at room temperature under an argon atmosphere. At this stage the H_2O was filtered off and the resulting white powder was dried in vacuo. Yield: 0.005 g, 90%. ^1H NMR (399.5 MHz, C_6D_6): δ = 7.08 (m, 4H), 6.76 (m, 2H), 6.62 (m, 4H), 4.71 (s, 2H). $^{13}C\{^1H\}$ NMR (100.46 MHz, C_6D_6): δ = 149.4, 129.5, 120.0, 112.7.

NMR resonances agree with that found for 1,2-diphenylhydrazine[3]

Based driven alcoholysis of 9 to form 1,2-diphenylhydrazine

9 (0.025 g, 0.06 mmol) and KO*t*Bu (0.013 g, 0.12 mmol) was dissolved in *i*PrOH/toluene (1:1, 2 ml). The resulting reaction mixture was stirred at room temperature for 22 h under an N_2 atmosphere. The volatiles were removed in vacuo and the product extracted with hexane (2 x 1 ml). Colourless crystals were obtained on recrystallizing the hexane extracts at –30 °C. Yield: 0.009 g, 85%. [See above for NMR assignment]

Spectroscopic Data

1,2-diphenyl-1,2-bis(4,4,5,5-tetramethyl-1,3,2-dioxaborolan-2-yl)hydrazine (2)

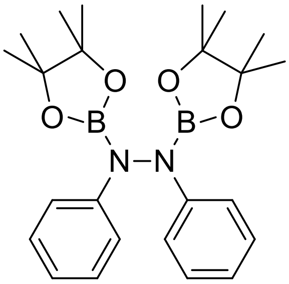

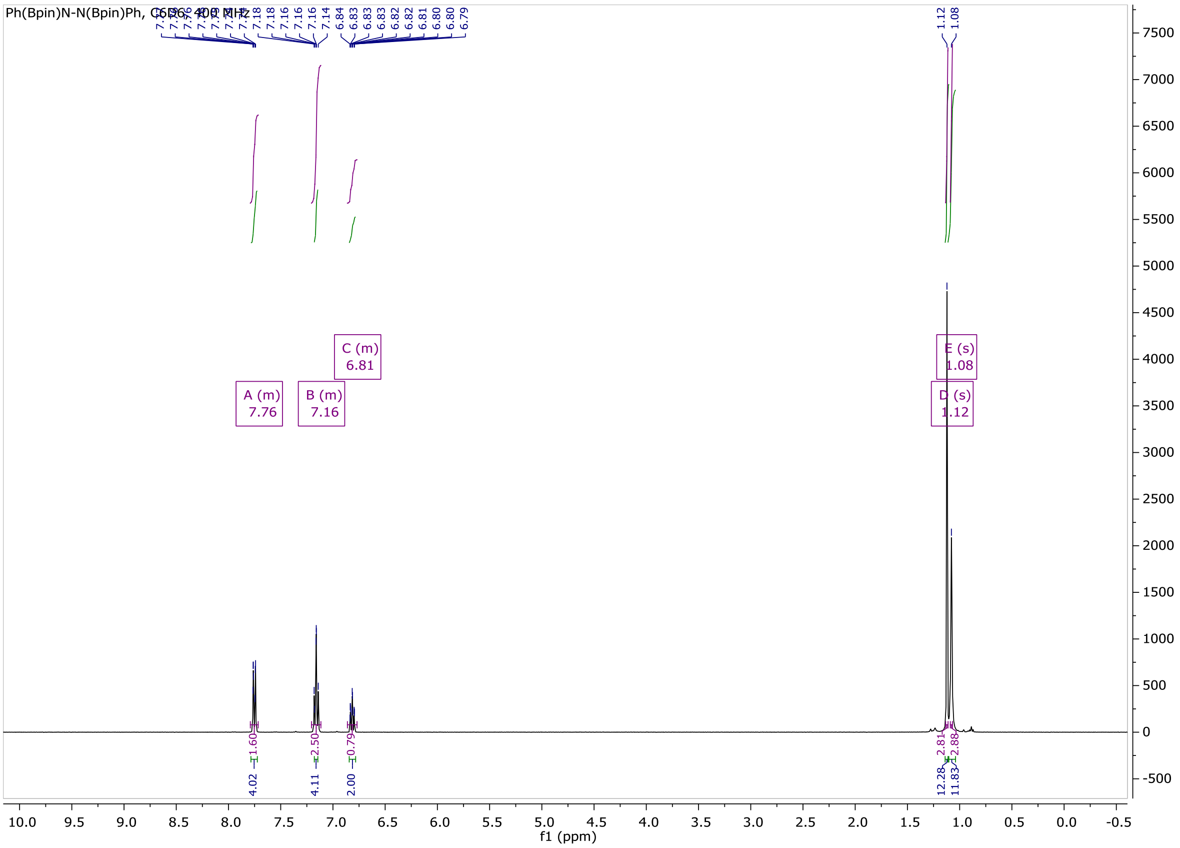

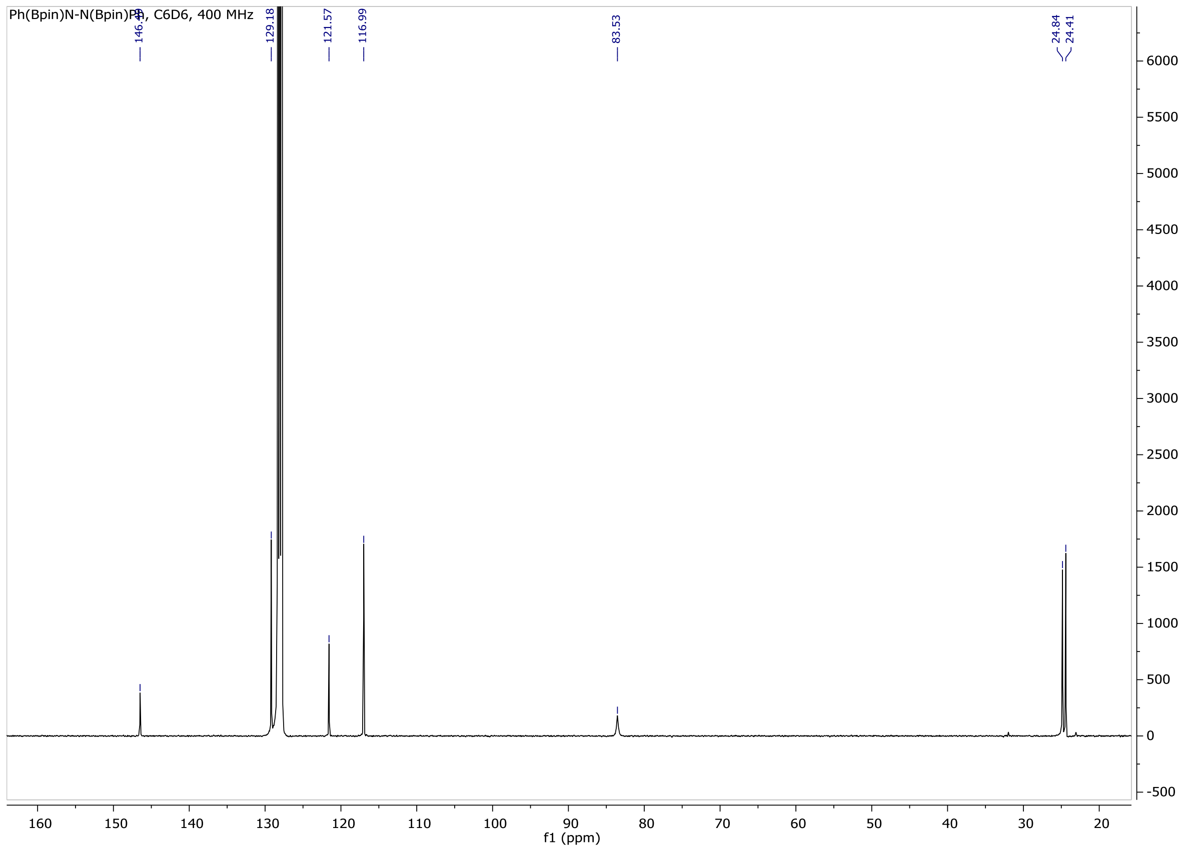
Ph(Bpin)N-N(Bpin)Ph, C6D6, 400 MHz
129.18
121.57
116.99
83.53
24.84
24.41
f1 (ppm)

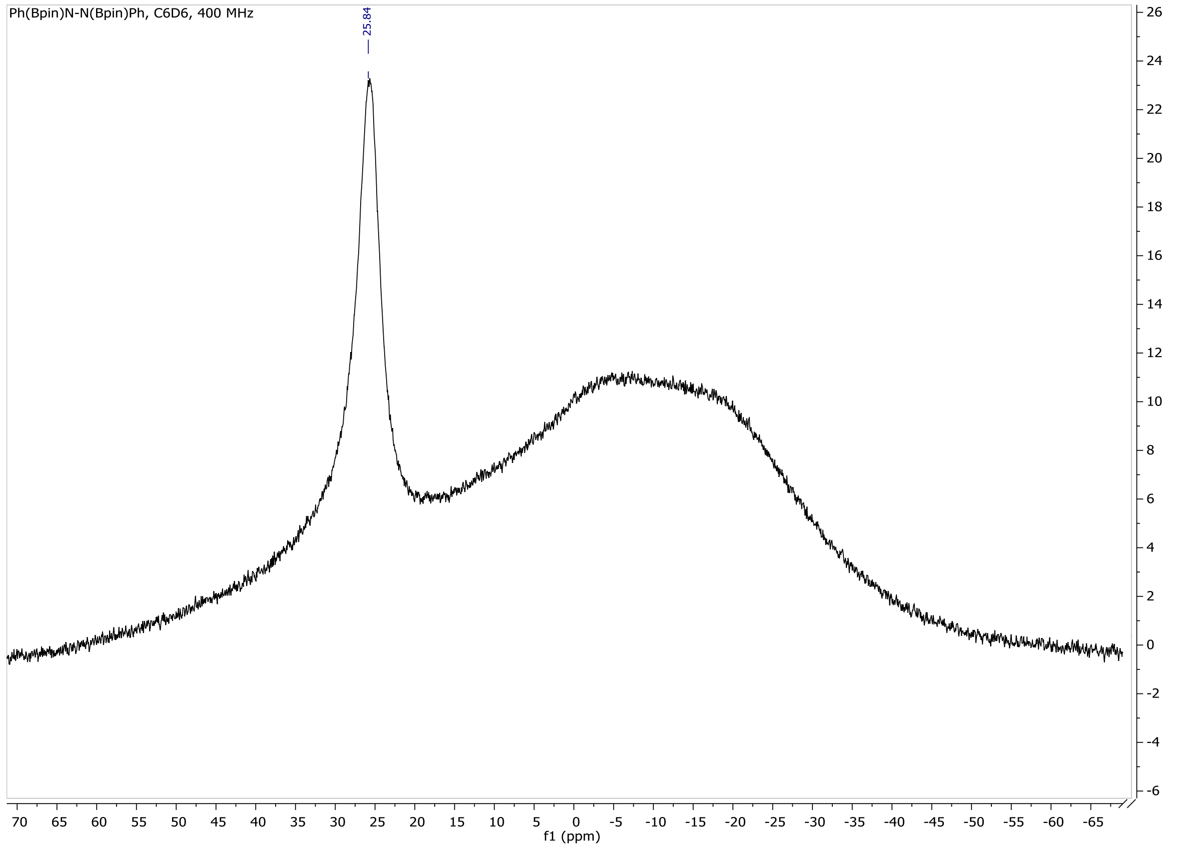
Ph(Bpin)N-N(Bpin)Ph, C6D6, 400 MHz
25.84
f1 (ppm)

1,2-bis(4,4,5,5-tetramethyl-1,3,2-dioxaborolan-2-yl)-1,2-di-m-tolylhydrazine (3)

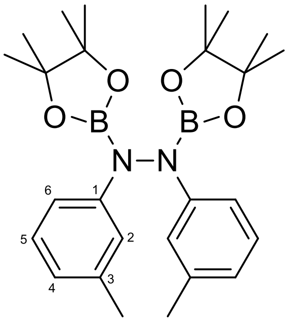

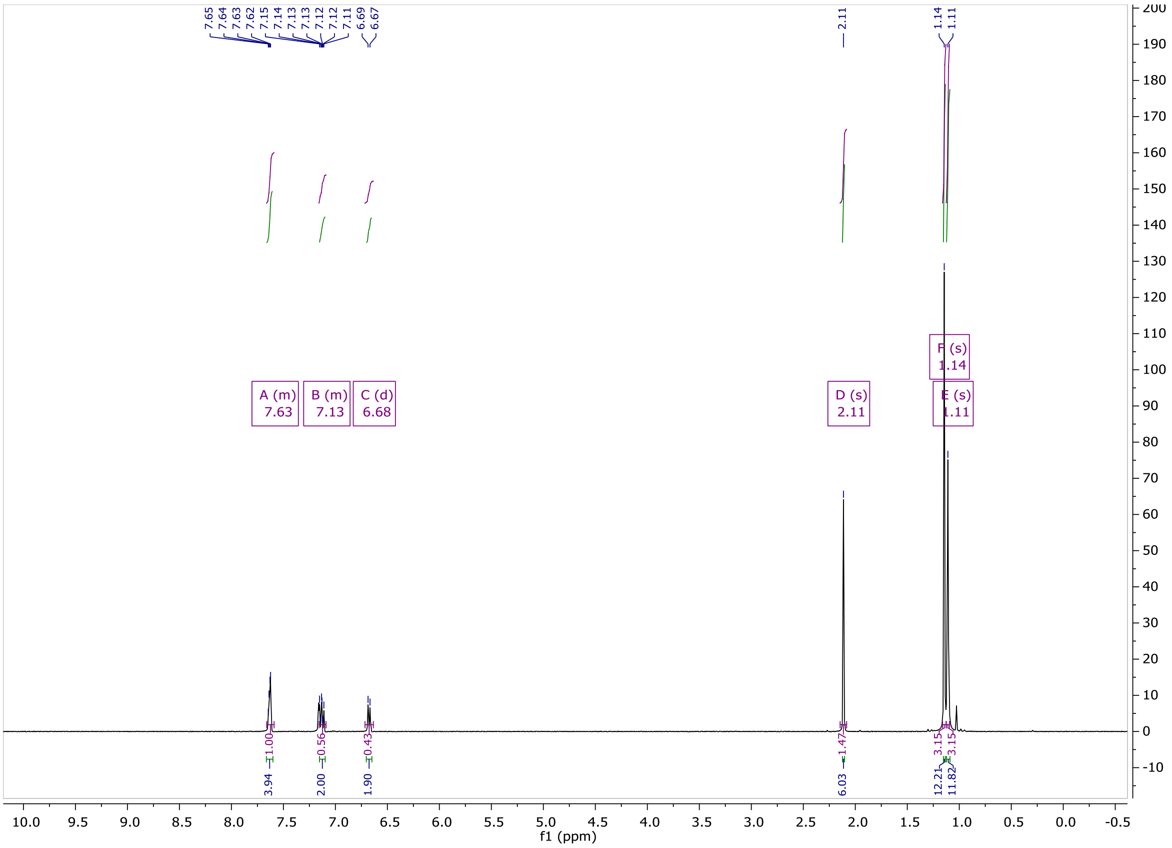

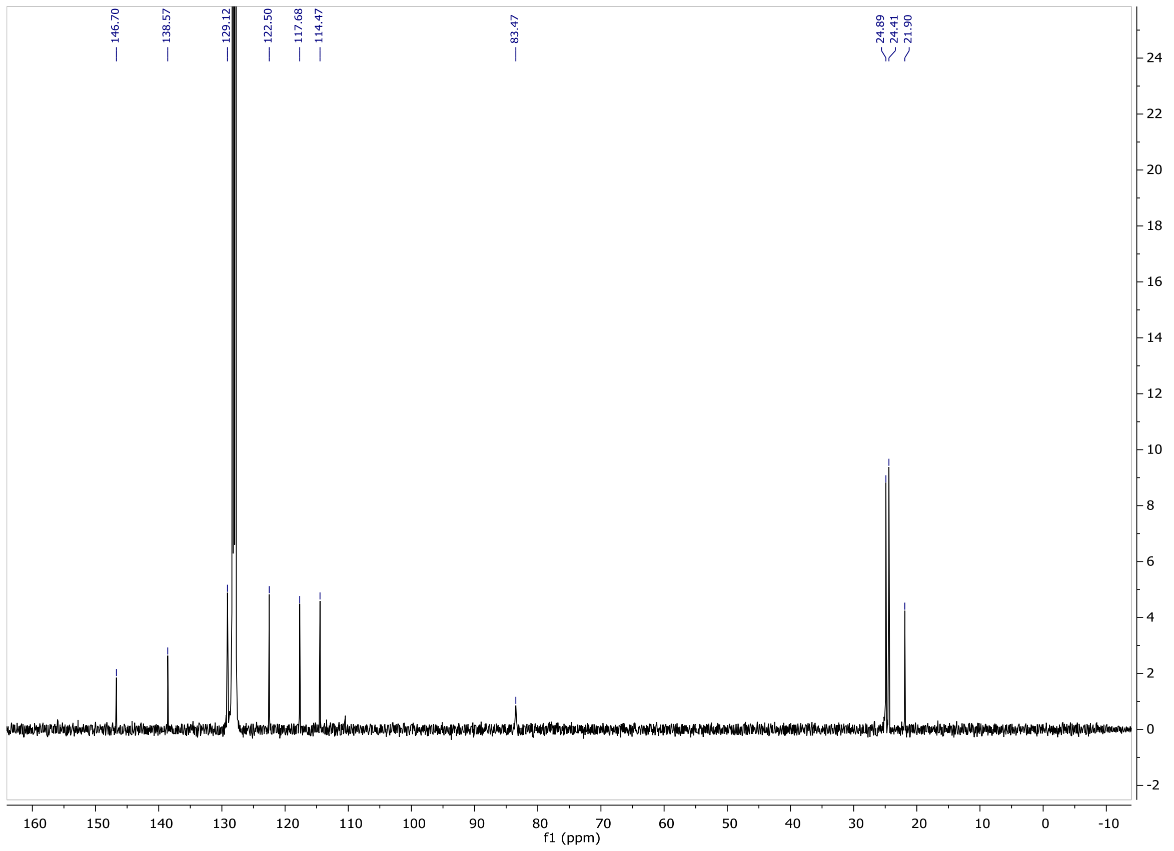
146.70
138.57
129.12
122.50
117.68
114.47
83.47
24.89
24.41
21.90
f1 (ppm)

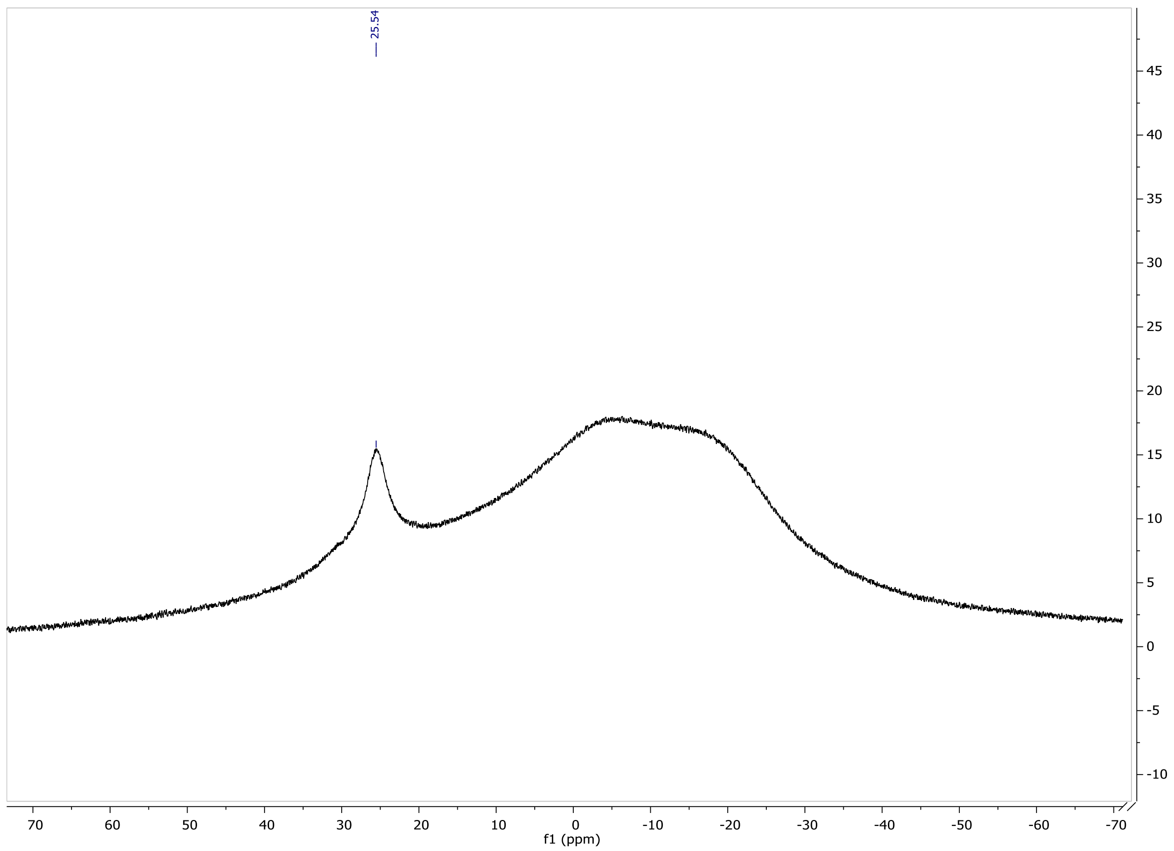
25.54
f1 (ppm)

1,2-bis(4,4,5,5-tetramethyl-1,3,2-dioxaborolan-2-yl)-1,2-di-p-tolylhydrazine (4)

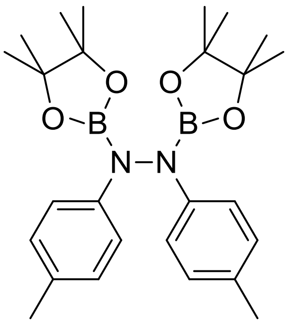

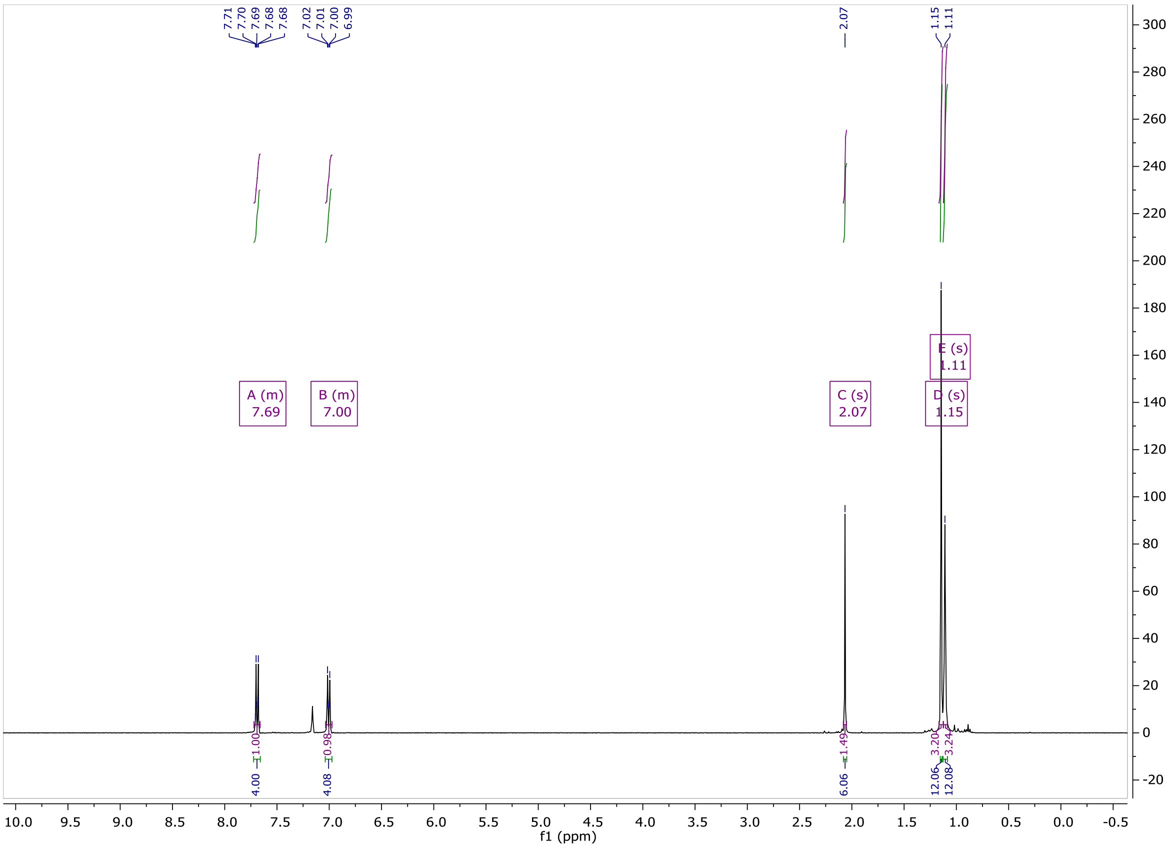

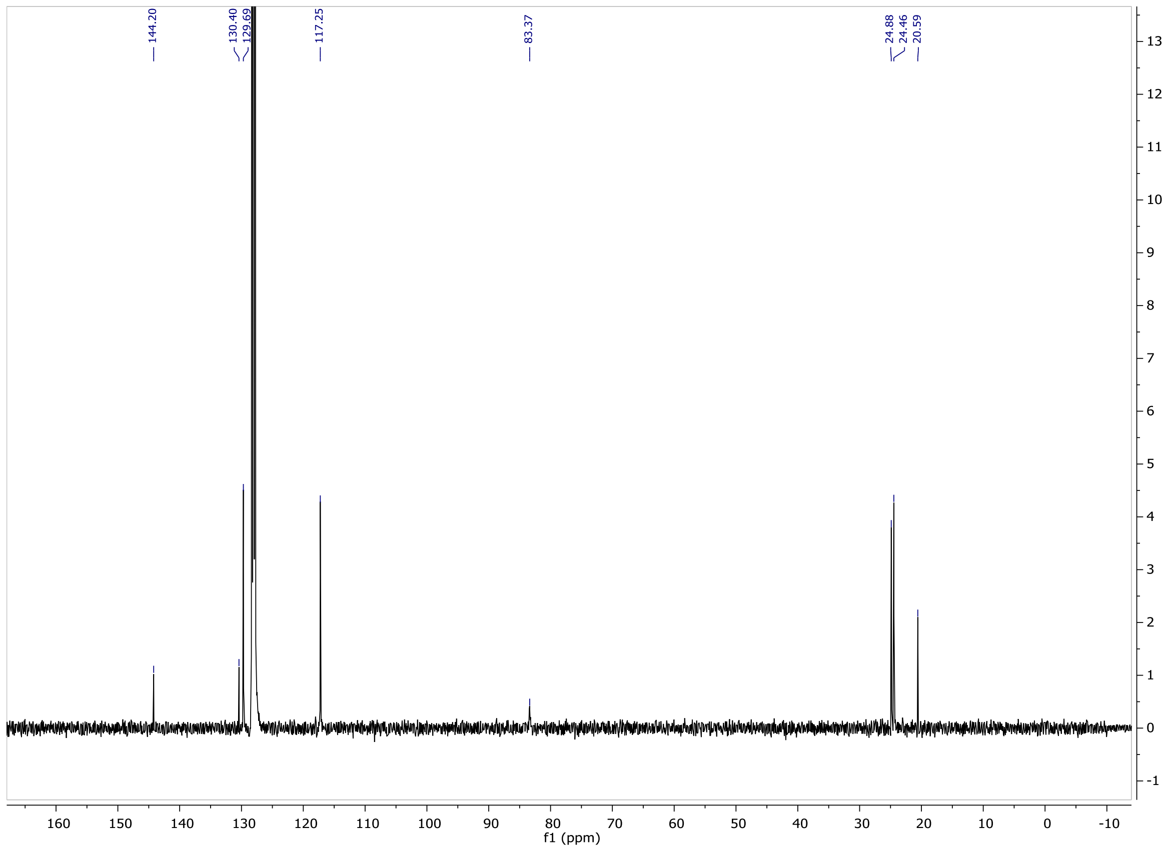
144.20
130.40
129.69
117.25
83.37
24.88
24.46
20.59
f1 (ppm)

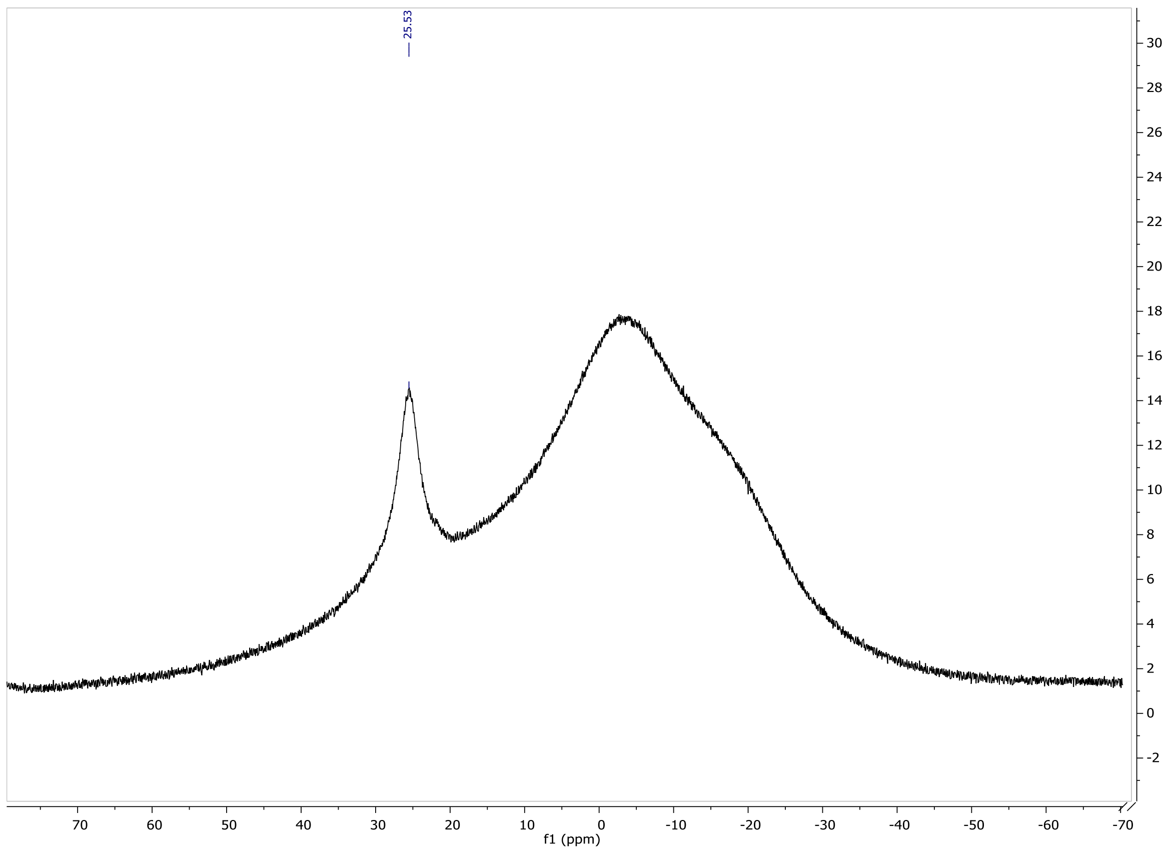
25.53
f1 (ppm)

1-(4-methoxyphenyl)-2-phenyl-1,2-bis(4,4,5,5-tetramethyl-1,3,2-dioxaborolan-2-yl)hydrazine (5)

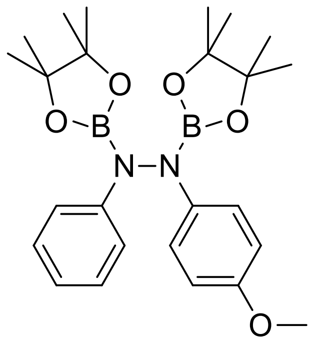

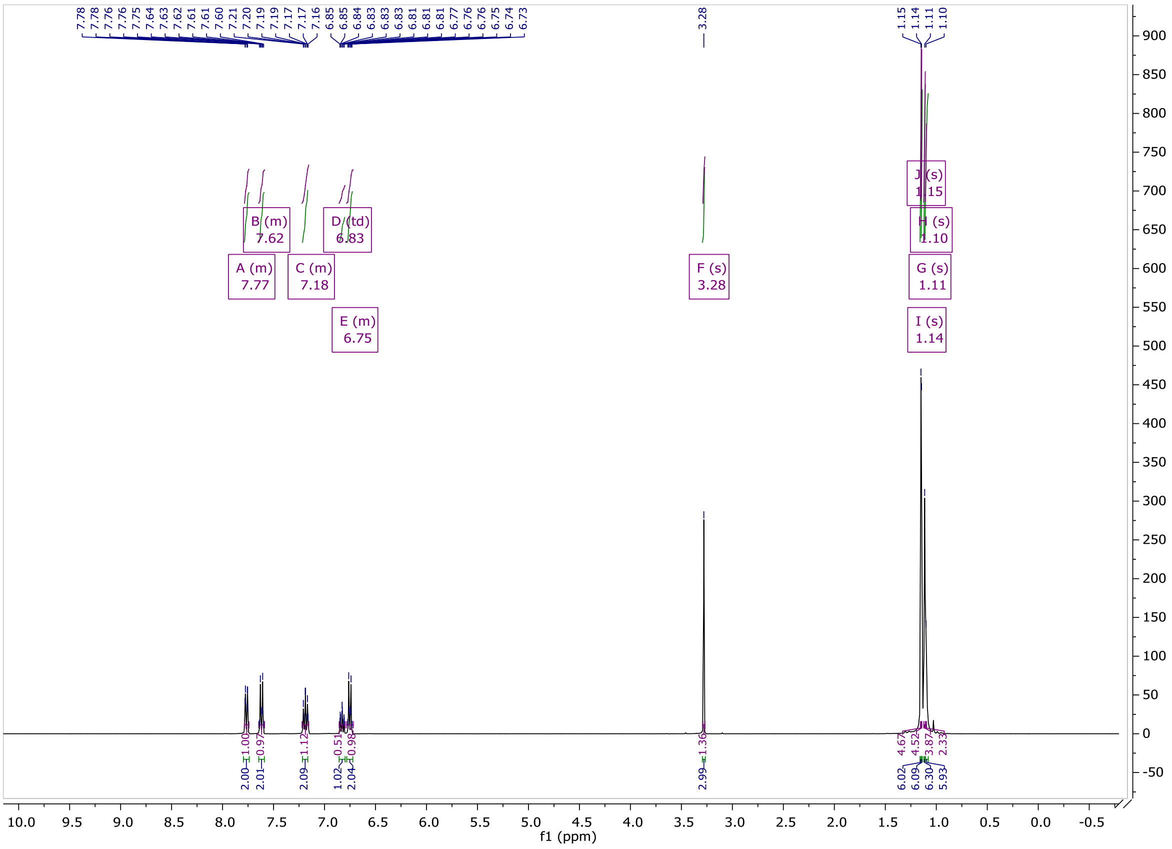

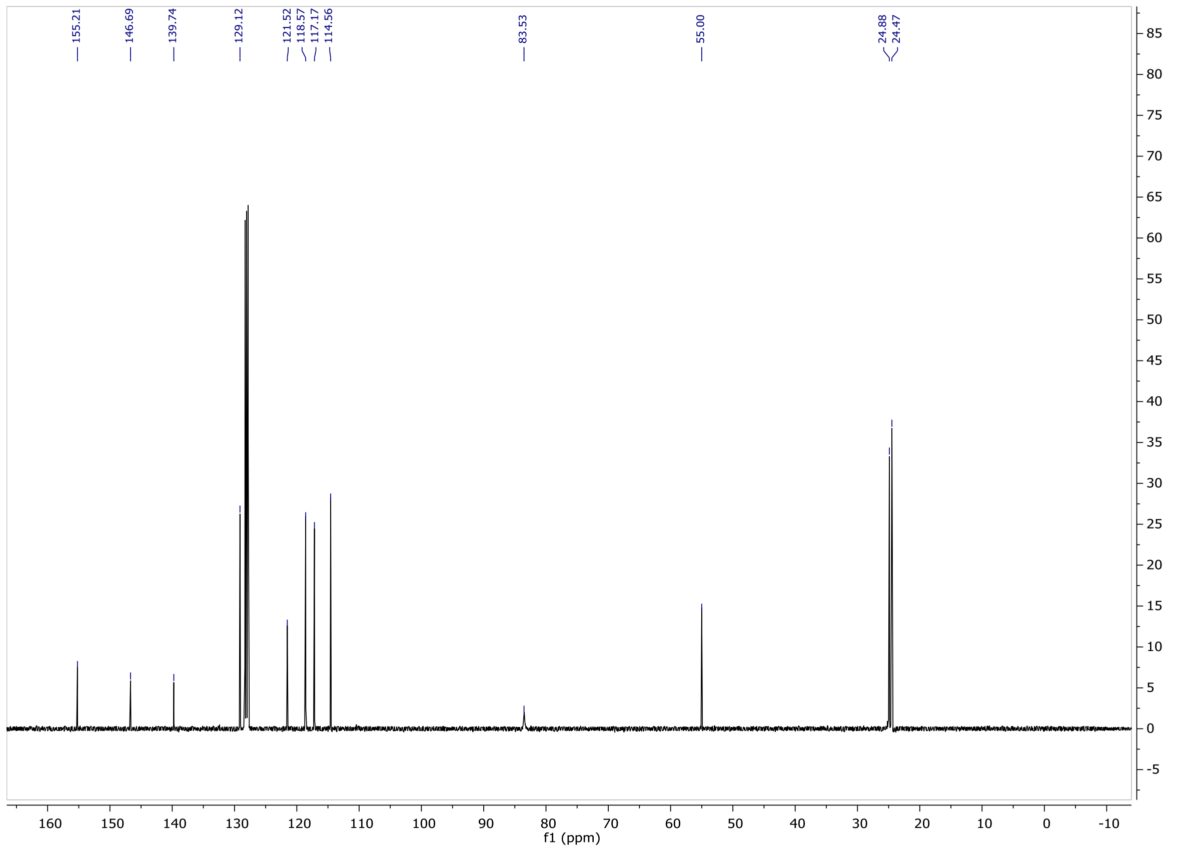
155.21
146.69
139.74
129.12
121.52
118.57
117.17
114.56
83.53
55.00
24.88
24.47
f1 (ppm)

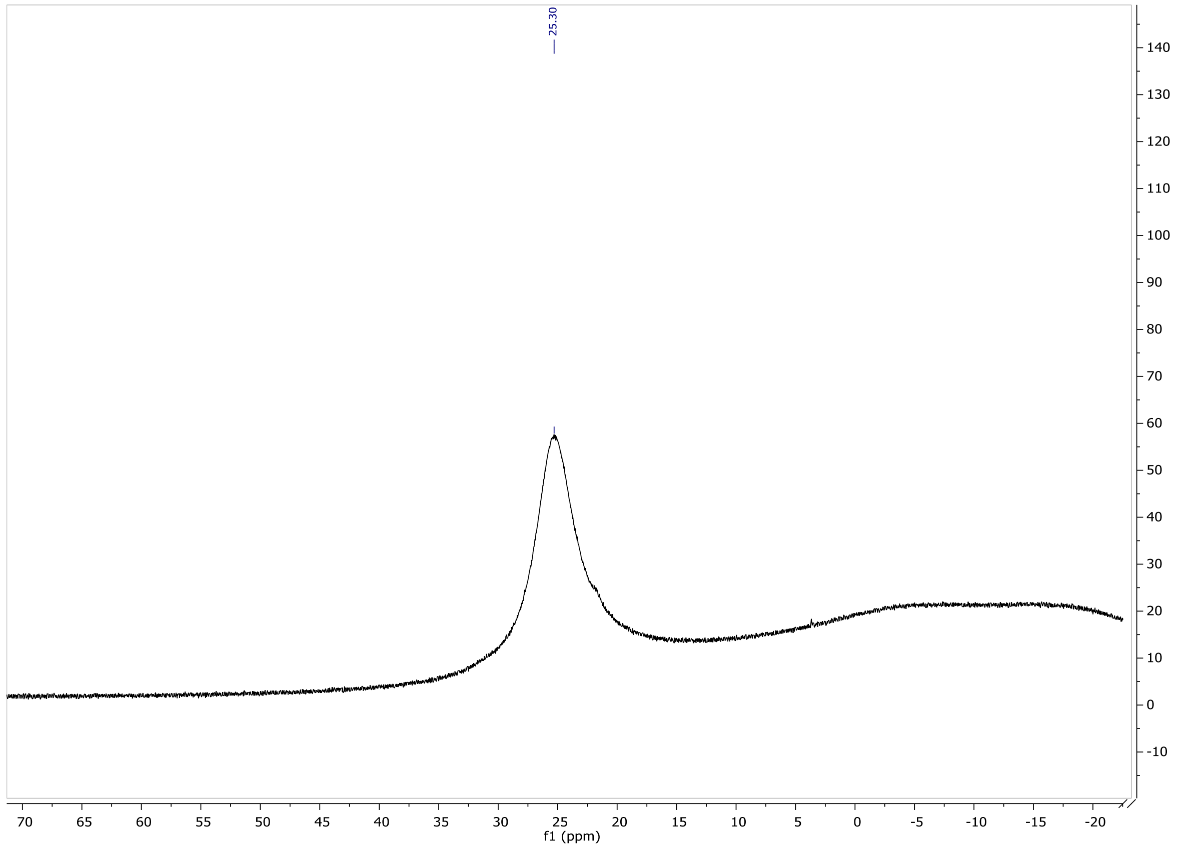
25.30
f1 (ppm)

N-(4-(1,2-bis(4,4,5,5-tetramethyl-1,3,2-dioxaborolan-2-yl)-2-(o-tolyl)hydrazinyl)-2-methylphenyl)acetamide (6)

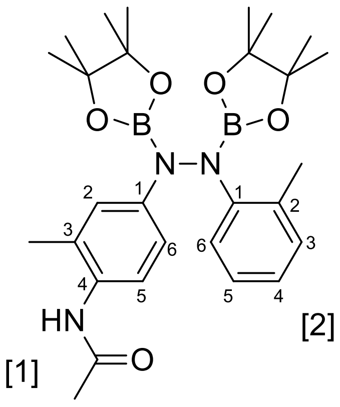

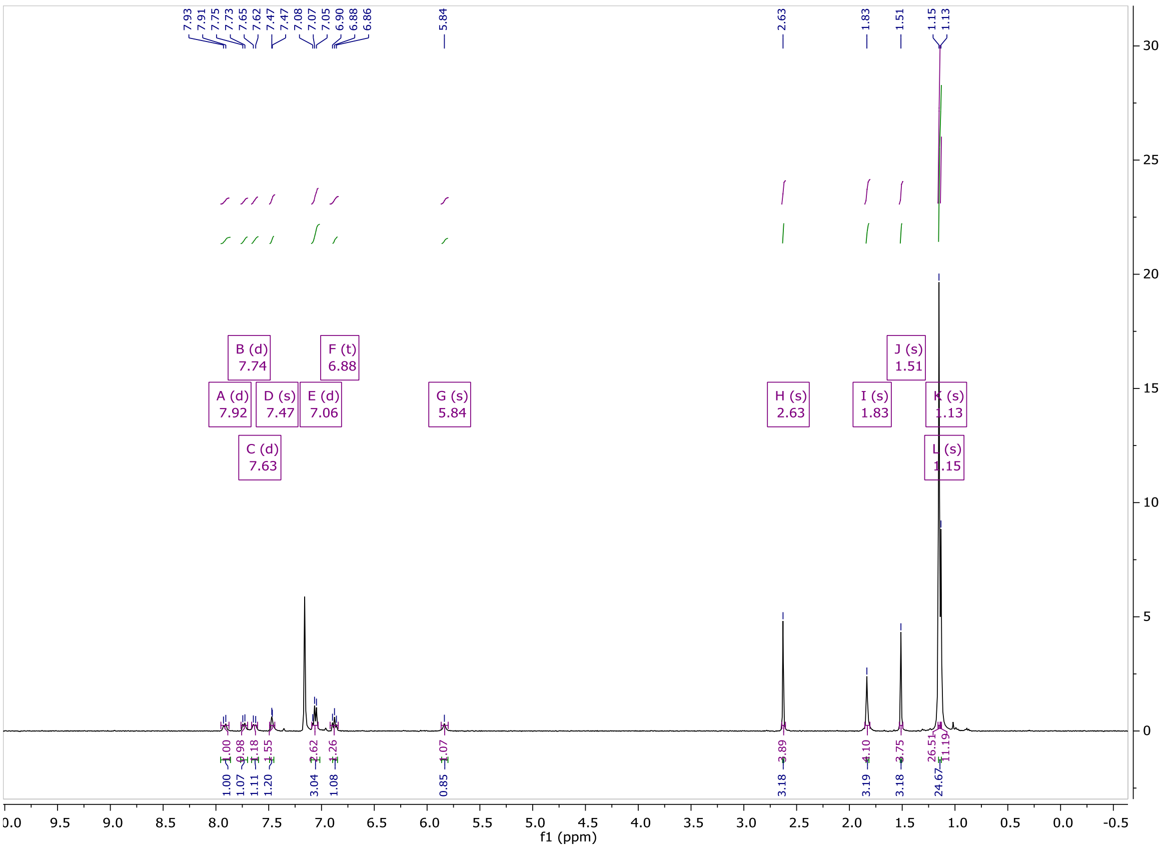

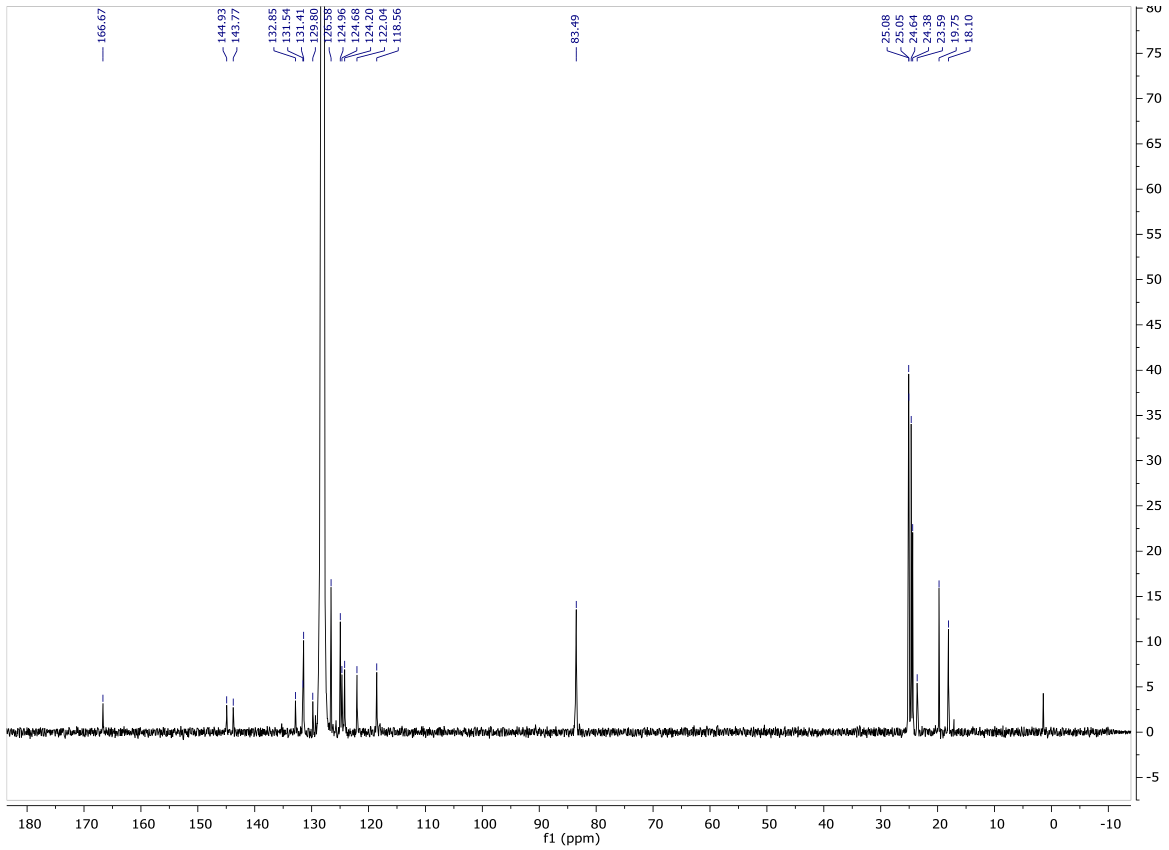
166.67
144.93
143.77
132.85
131.54
131.41
129.80
126.58
124.96
124.68
124.20
122.04
118.56
83.49
25.08
25.05
24.64
24.38
23.59
19.75
18.10
f1 (ppm)

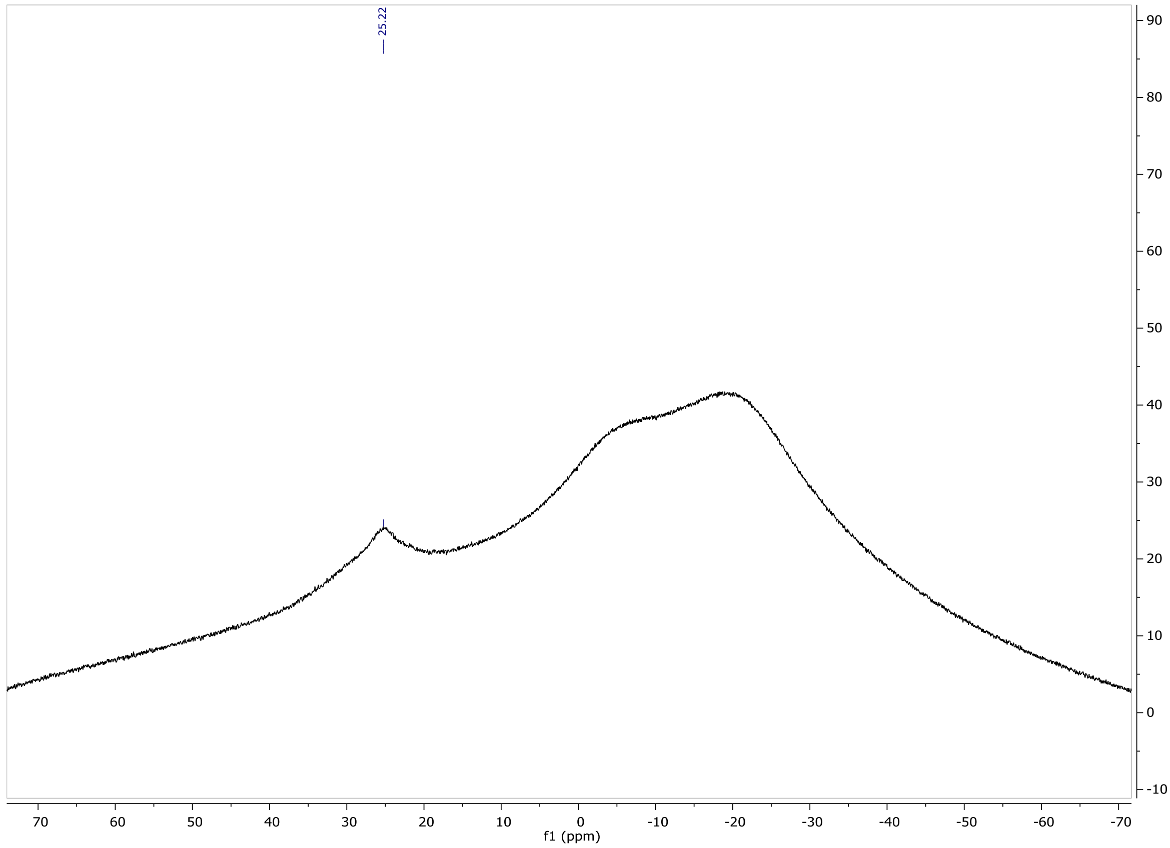
25.22
f1 (ppm)

1,2-bis(5,5-dimethyl-1,3,2-dioxaborinan-2-yl)-1,2-diphenylhydrazine (7)

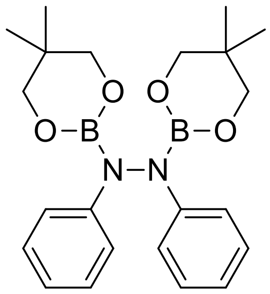

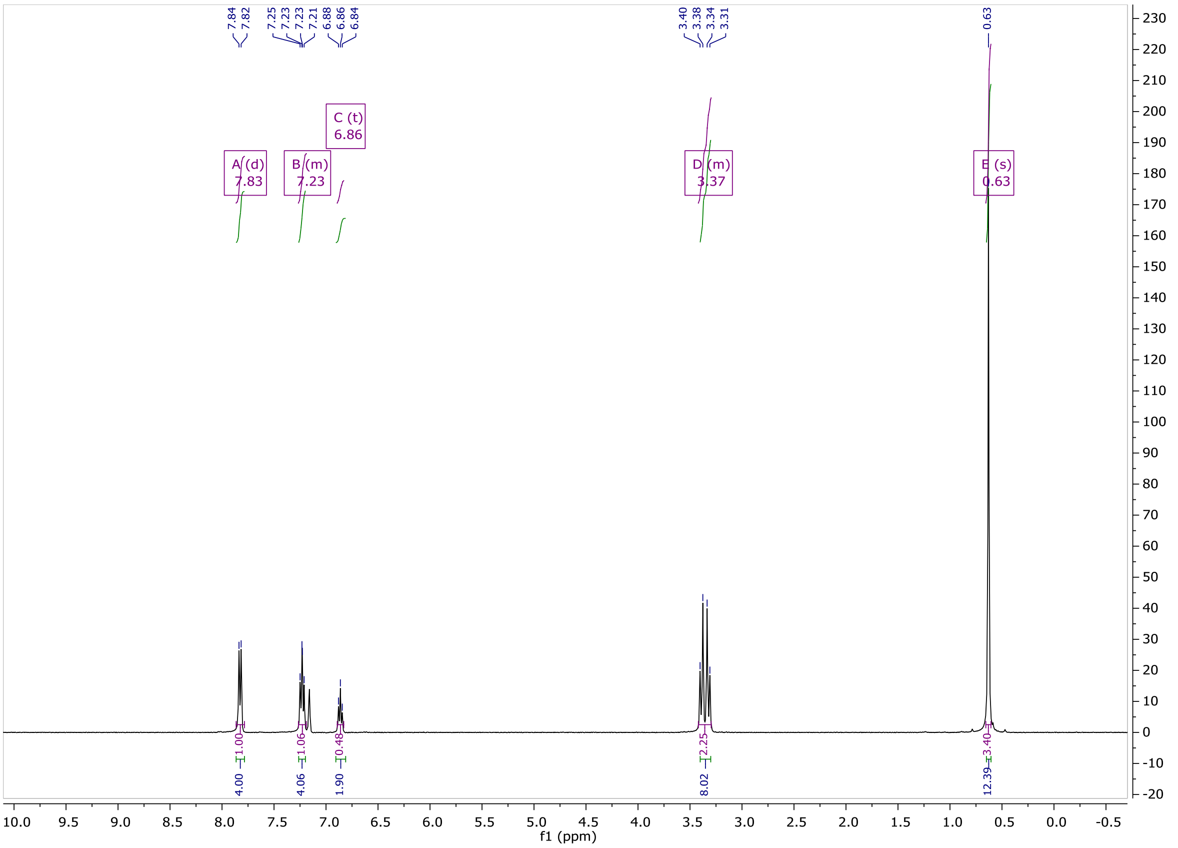

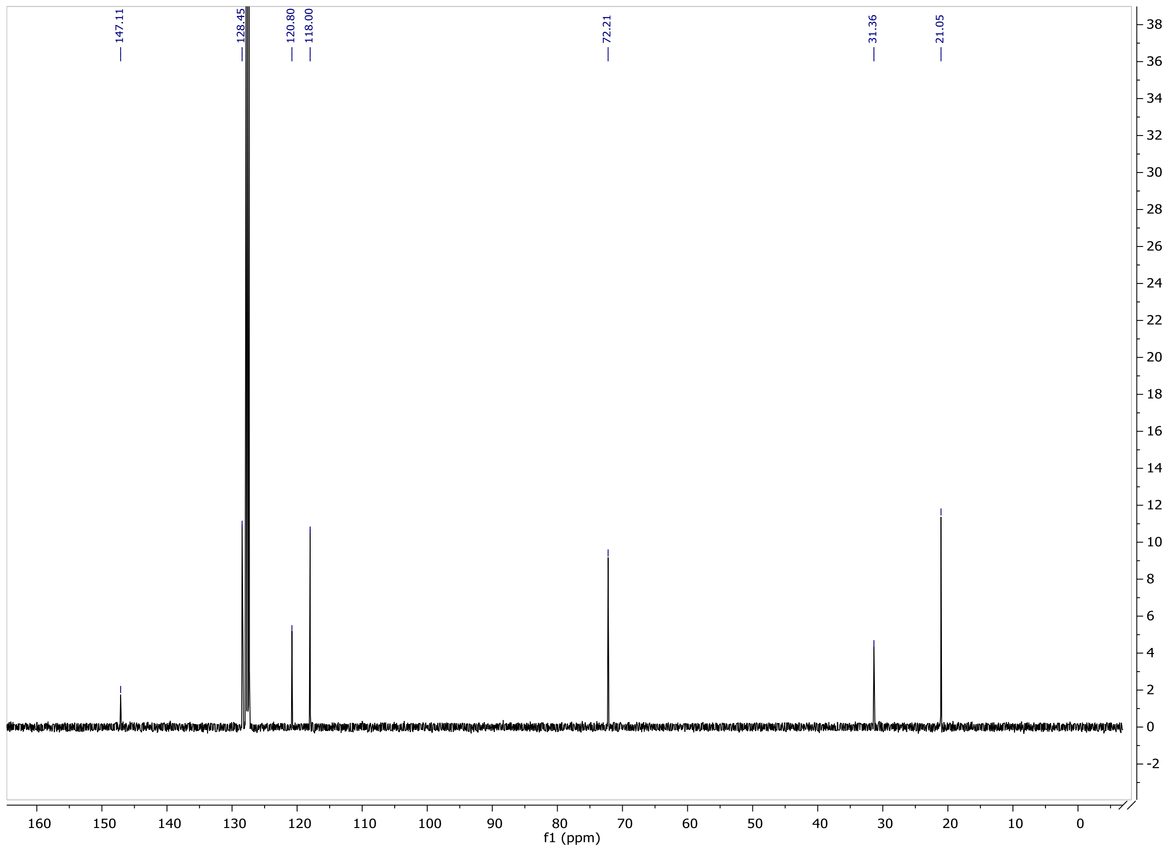
147.11
128.45
120.80
118.00
72.21
31.36
21.05
f1 (ppm)

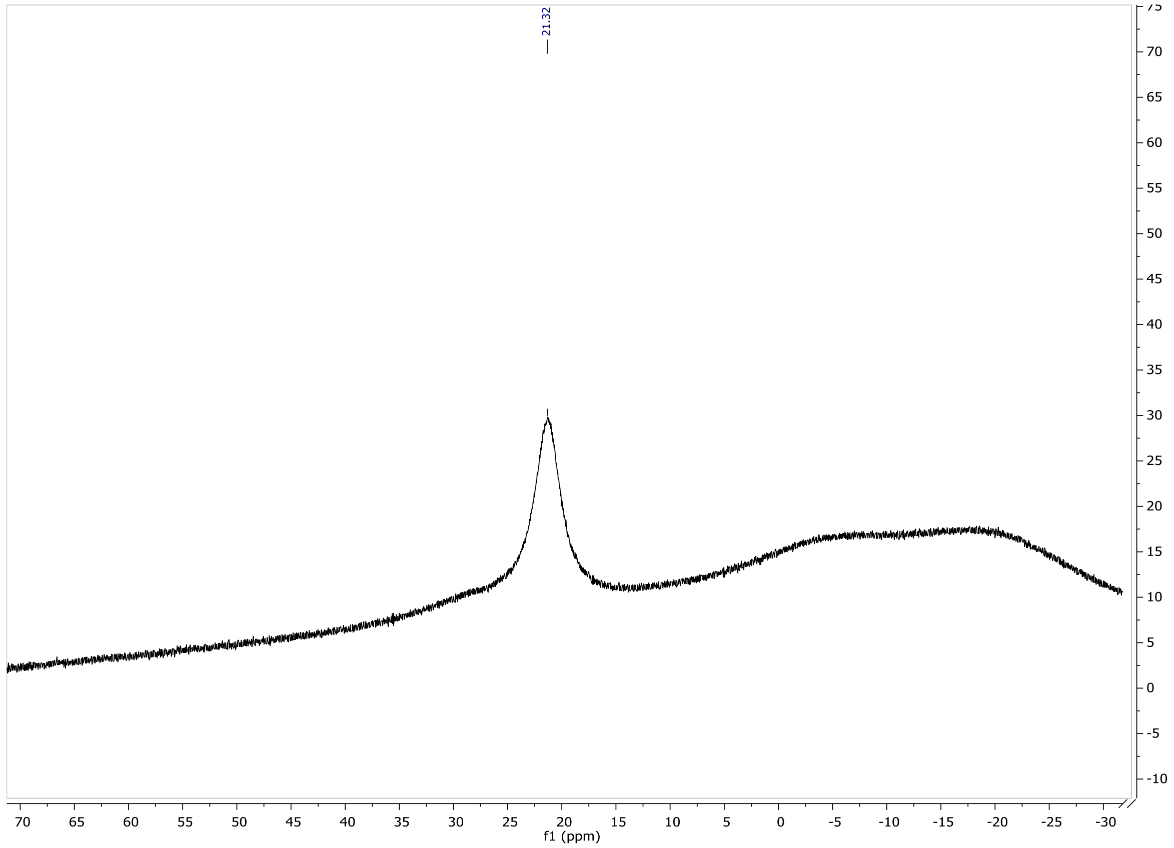
21.32
f1 (ppm)

1,2-bis(benzo[d][1,3,2]dioxaborol-2-yl)-1,2-diphenylhydrazine (8)

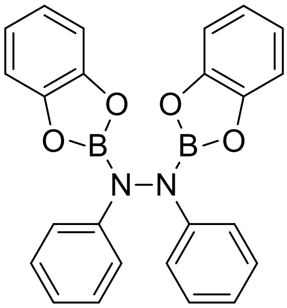

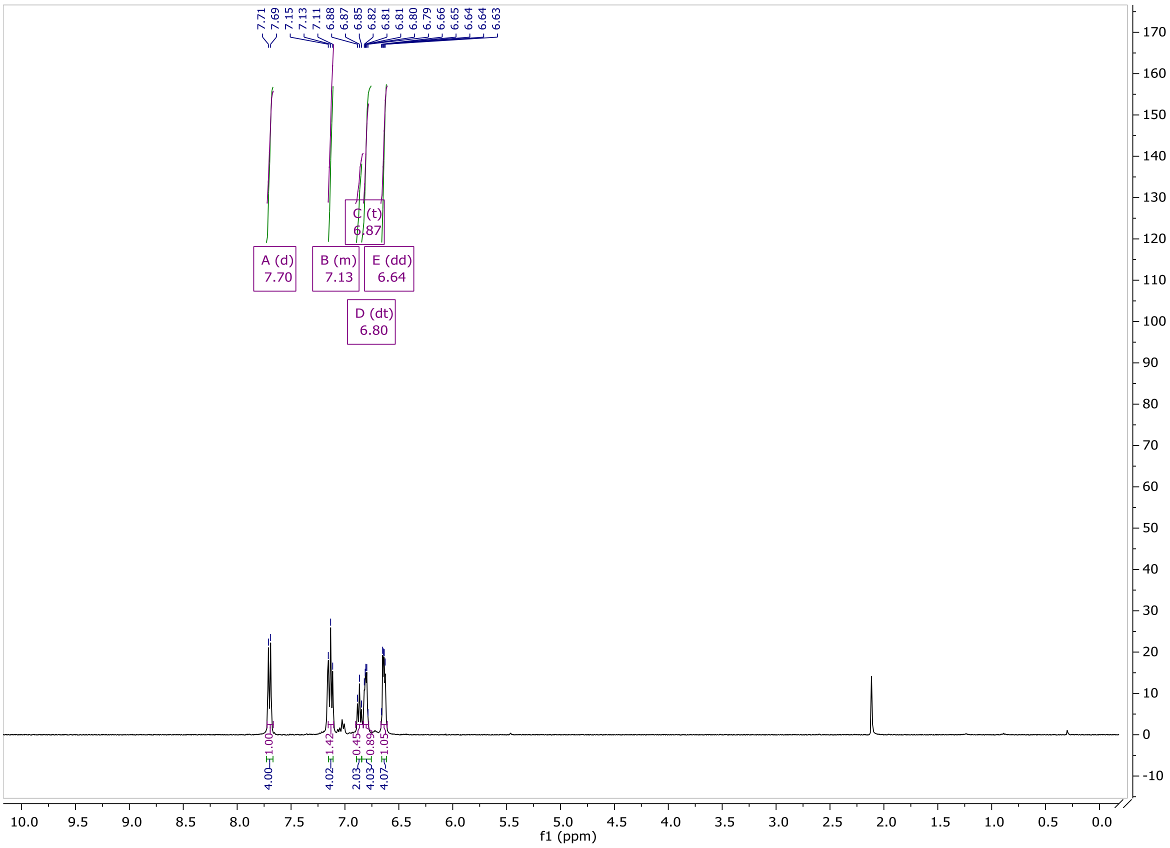

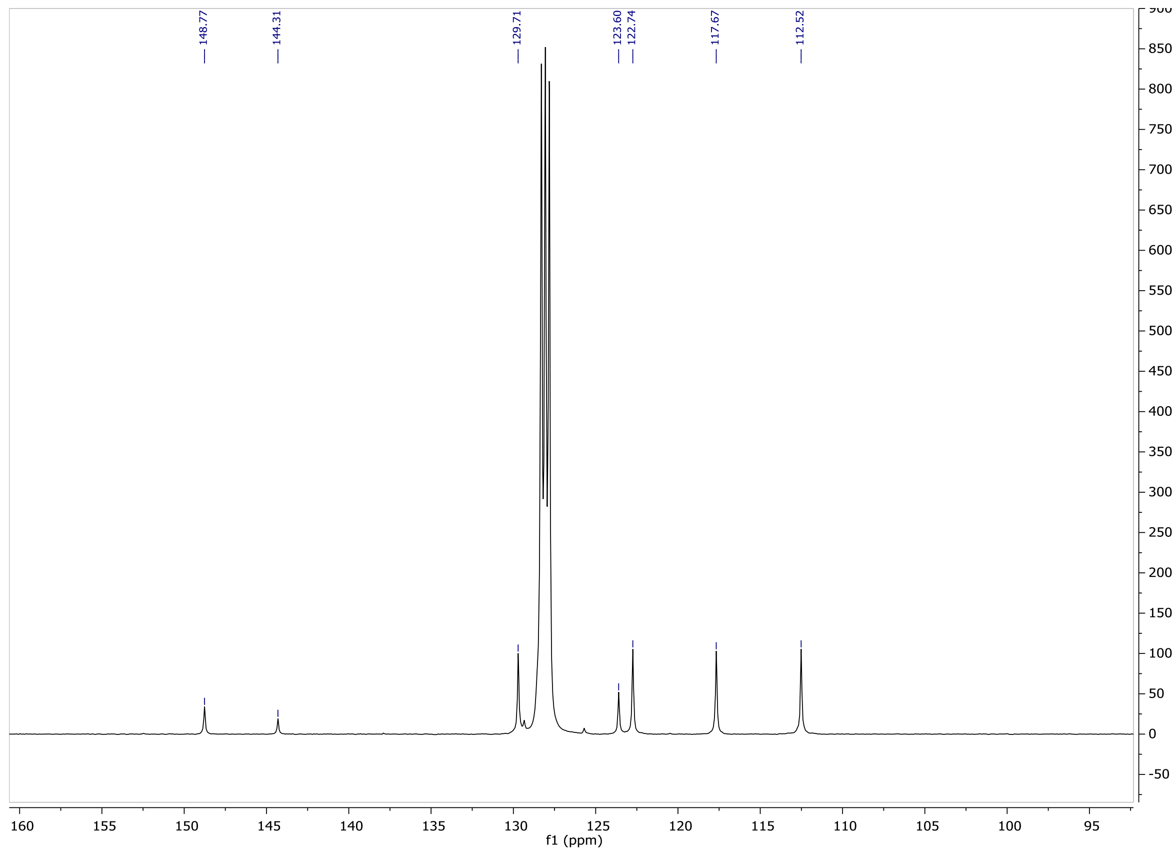
148.77
144.31
129.71
123.60
122.74
117.67
112.52
f1 (ppm)

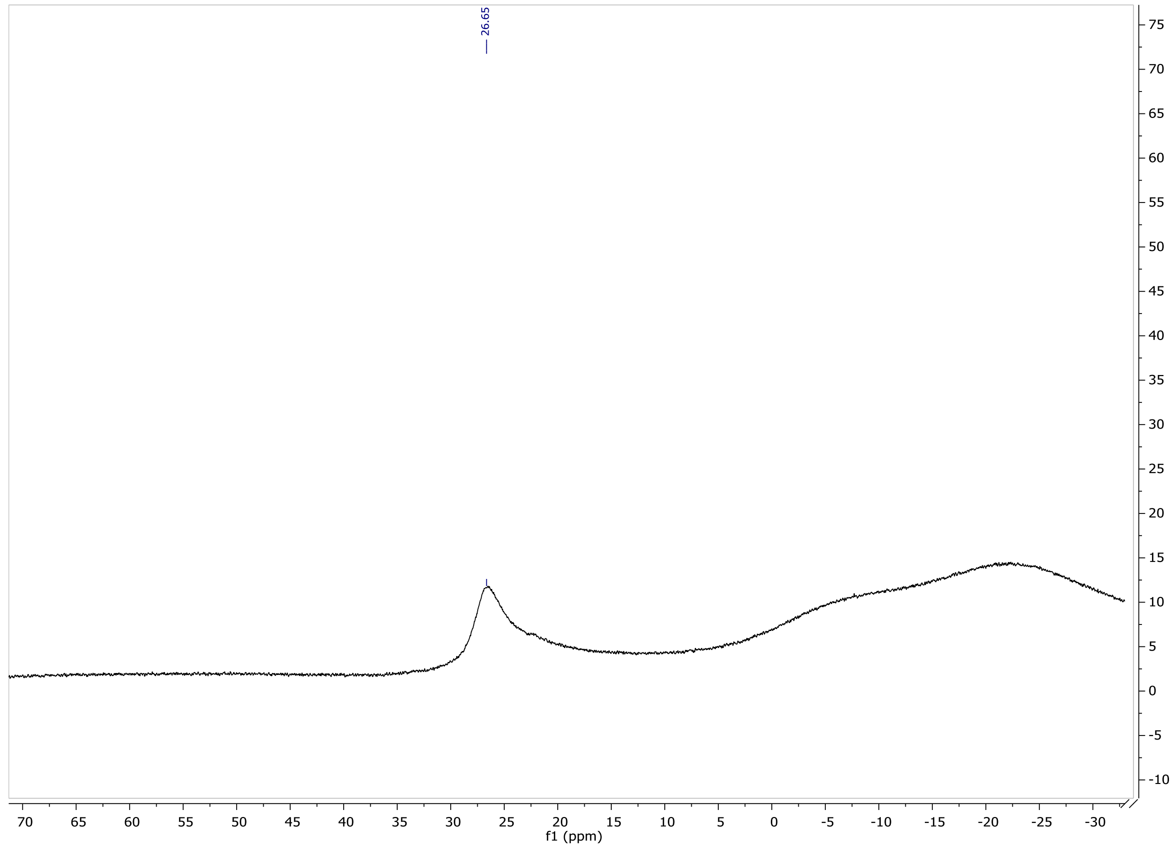
26.65
f1 (ppm)

1-(dimethyl(phenyl)silyl)-1,2-diphenyl-2-(4,4,5,5-tetramethyl-1,3,2-dioxaborolan-2-yl)hydrazine (9)

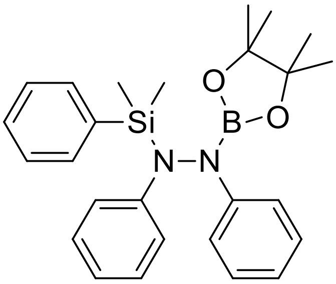

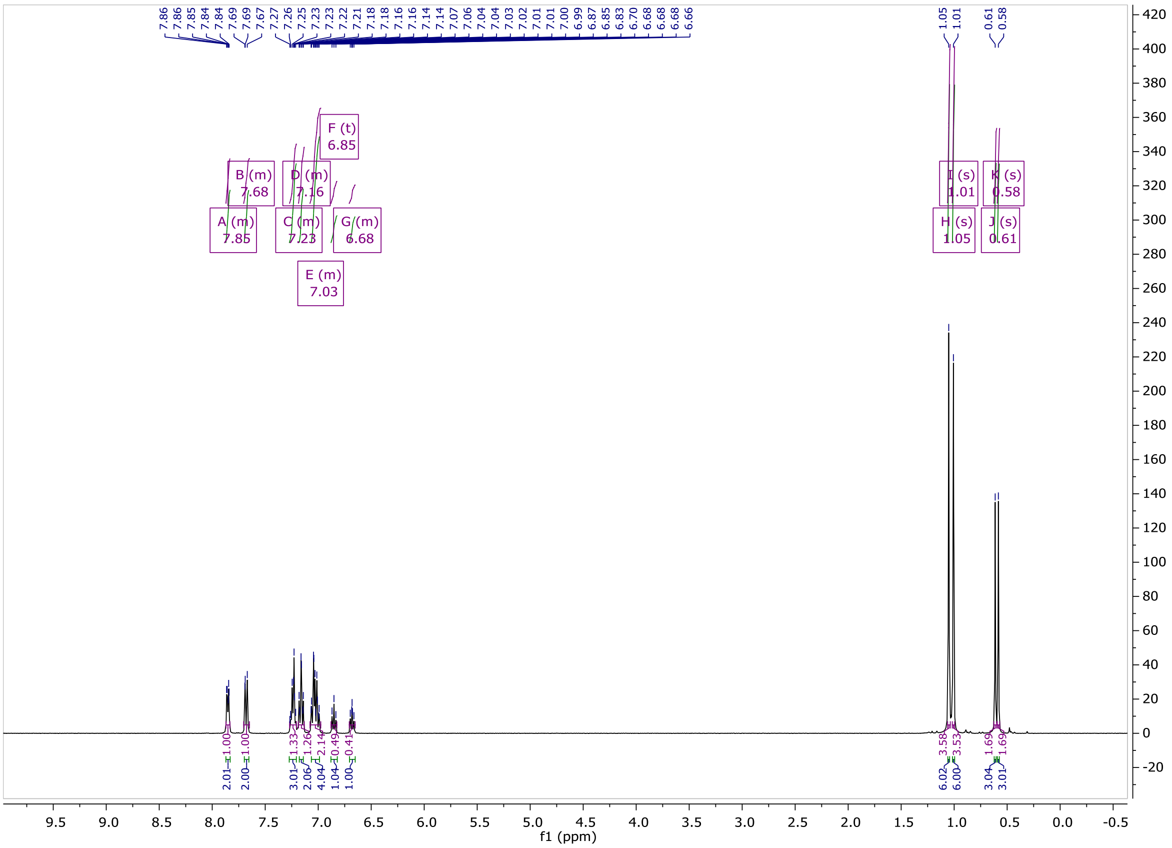

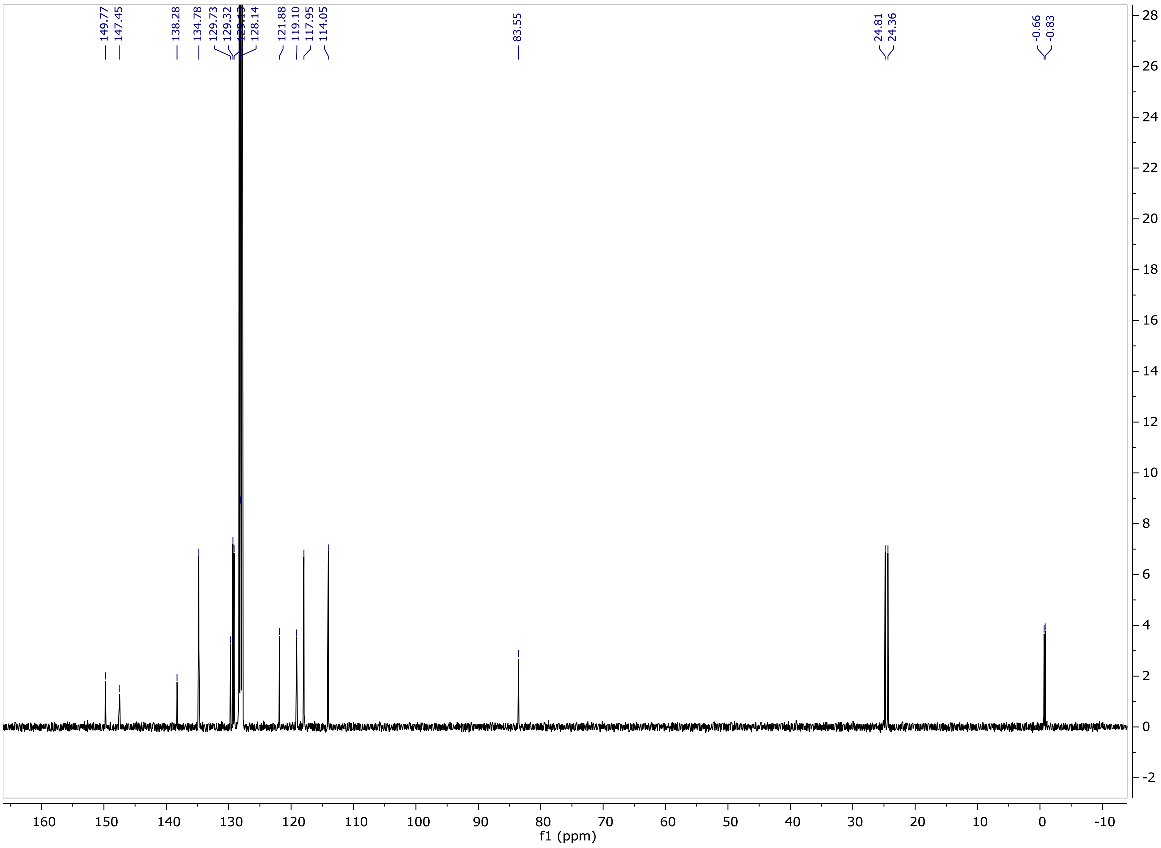
149.77
147.45
138.28
134.78
129.73
129.32
128.14
121.88
119.10
117.95
114.05
83.55
24.81
24.36
-0.66
-0.83
f1 (ppm)

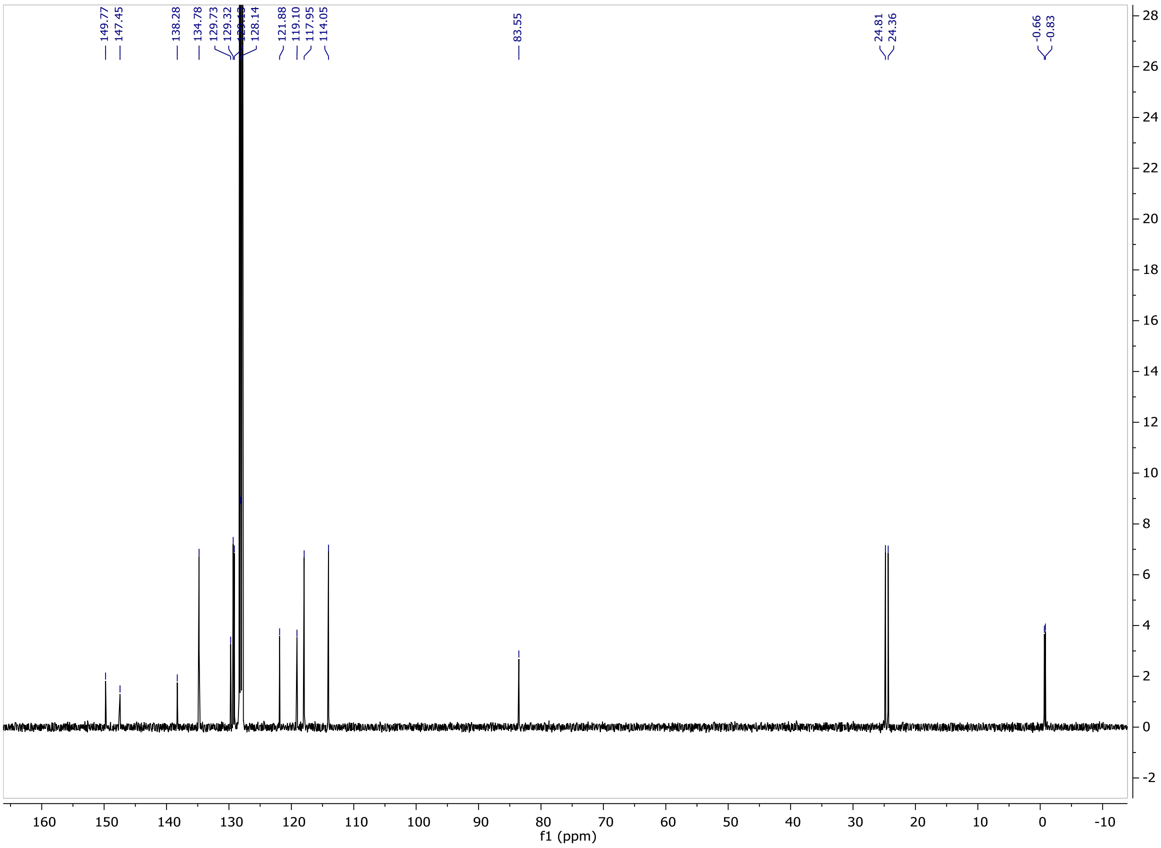
149.77
147.45
138.28
134.78
129.73
129.32
128.14
121.88
119.10
117.95
114.05
83.55
24.81
24.36
-0.66
-0.83
f1 (ppm)

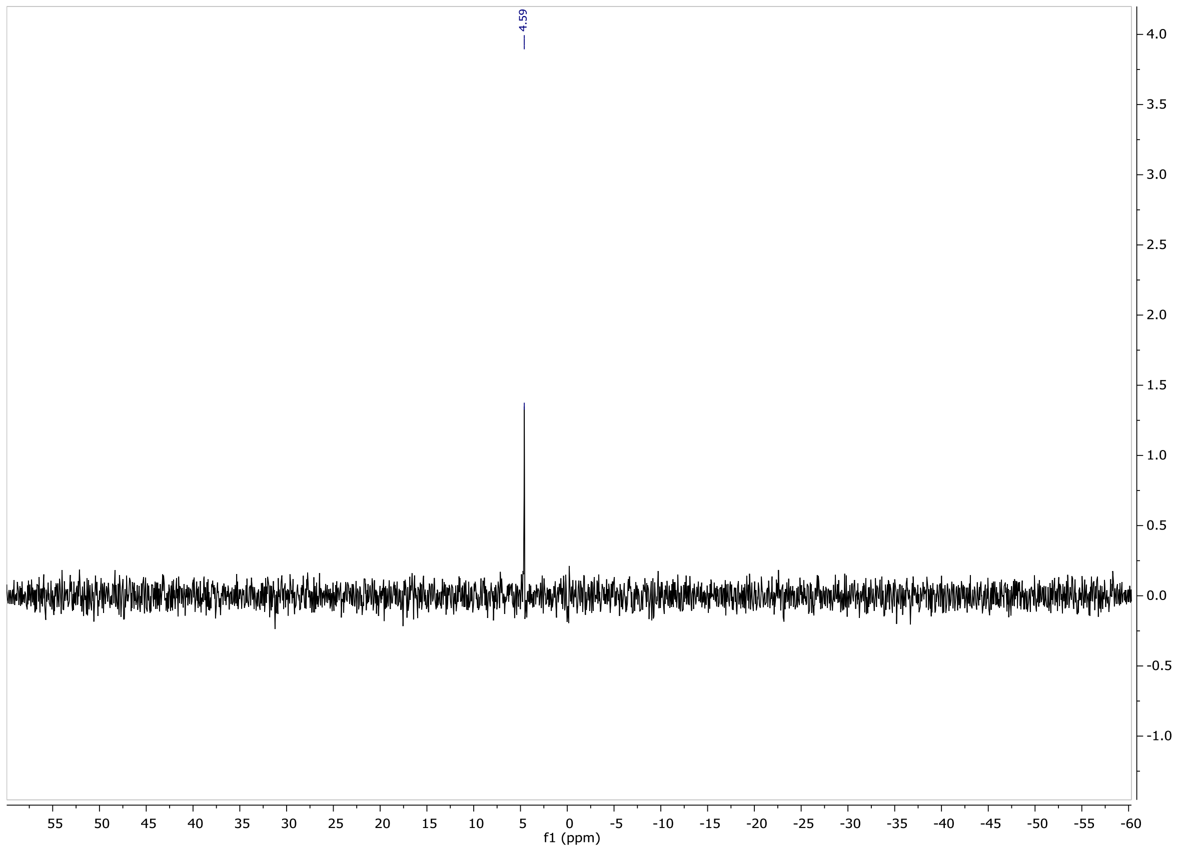
4.59
4.0
3.5
3.0
2.5
2.0
1.5
1.0
0.5
0.0
-0.5
-1.0
55
50
45
40
35
30
25
20
15
10
5
0
-5
-10
-15
-20
-25
-30
-35
-40
-45
-50
-55
-60
f1 (ppm)

1-(dimethyl(phenyl)silyl)-2-(4,4,5,5-tetramethyl-1,3,2-dioxaborolan-2-yl)-1,2-di-m-tolylhydrazine (10)

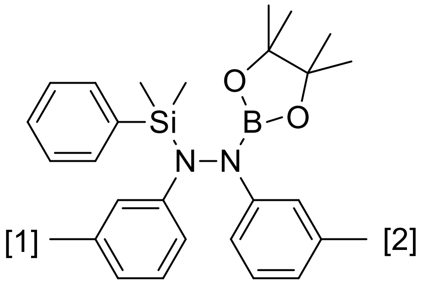

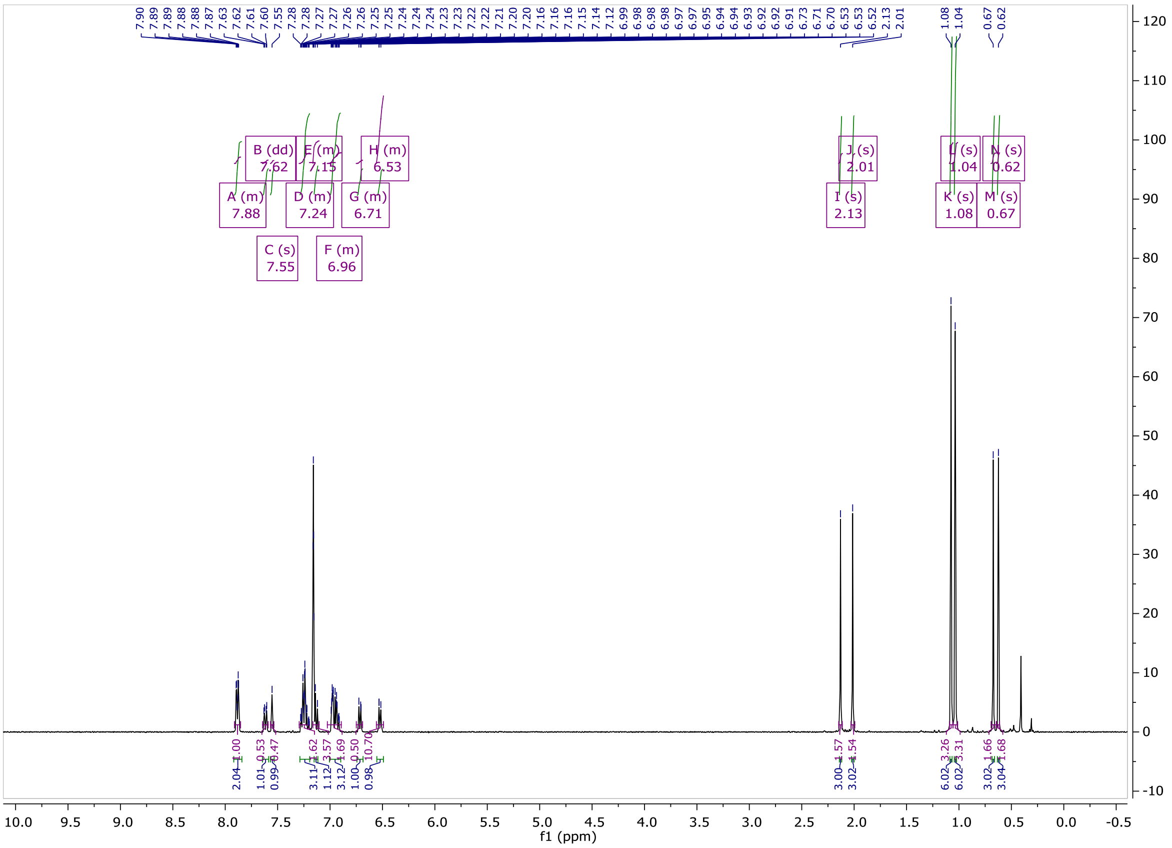

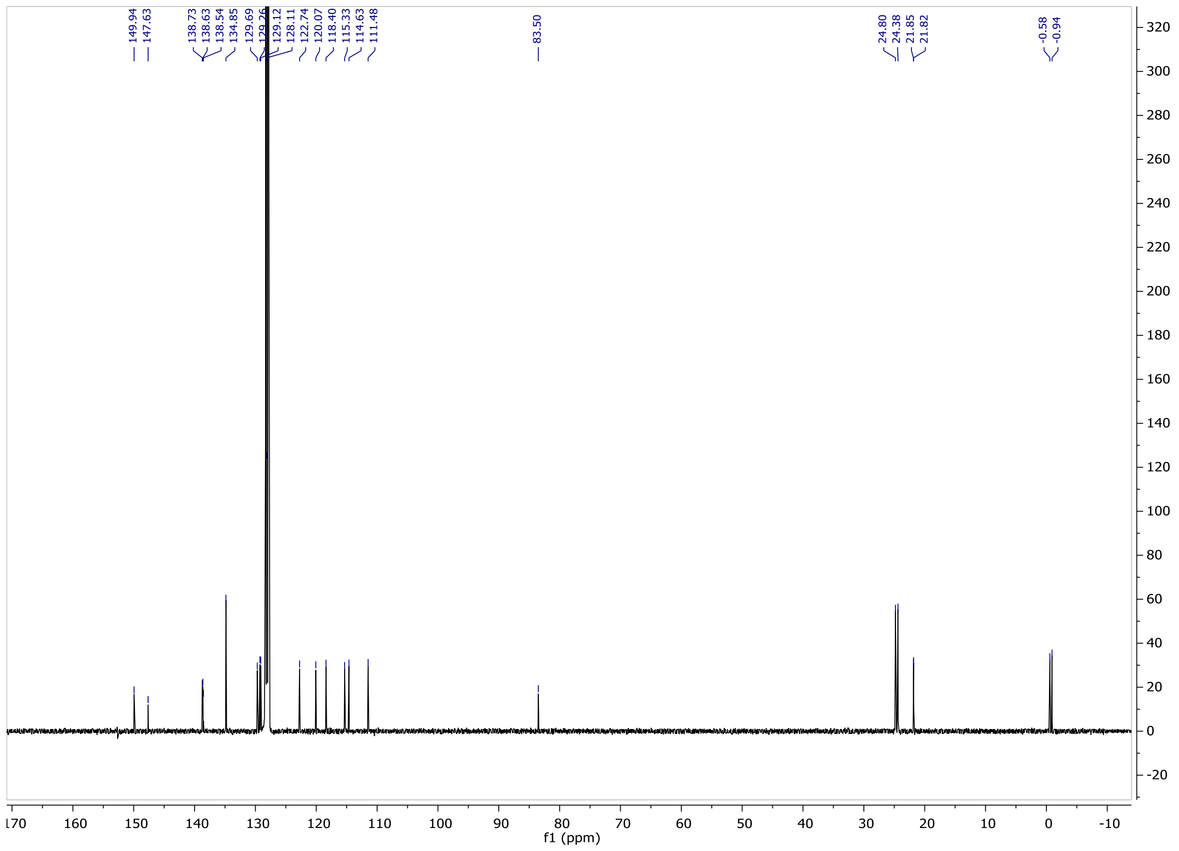
149.94
147.63
138.73
138.63
138.54
134.85
129.69
129.26
129.12
128.11
122.74
120.07
118.40
115.33
114.63
111.48
83.50
24.80
24.38
21.85
21.82
-0.58
-0.94
f1 (ppm)

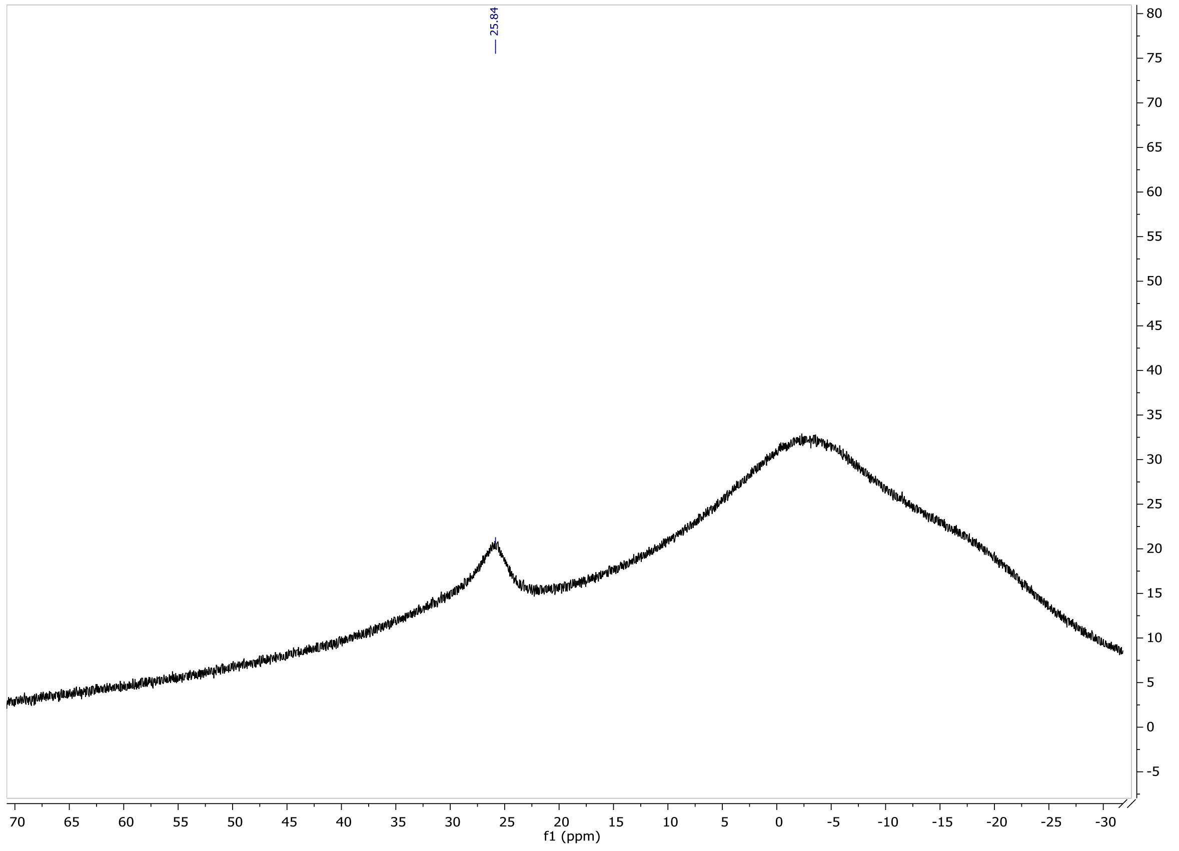
25.84
f1 (ppm)

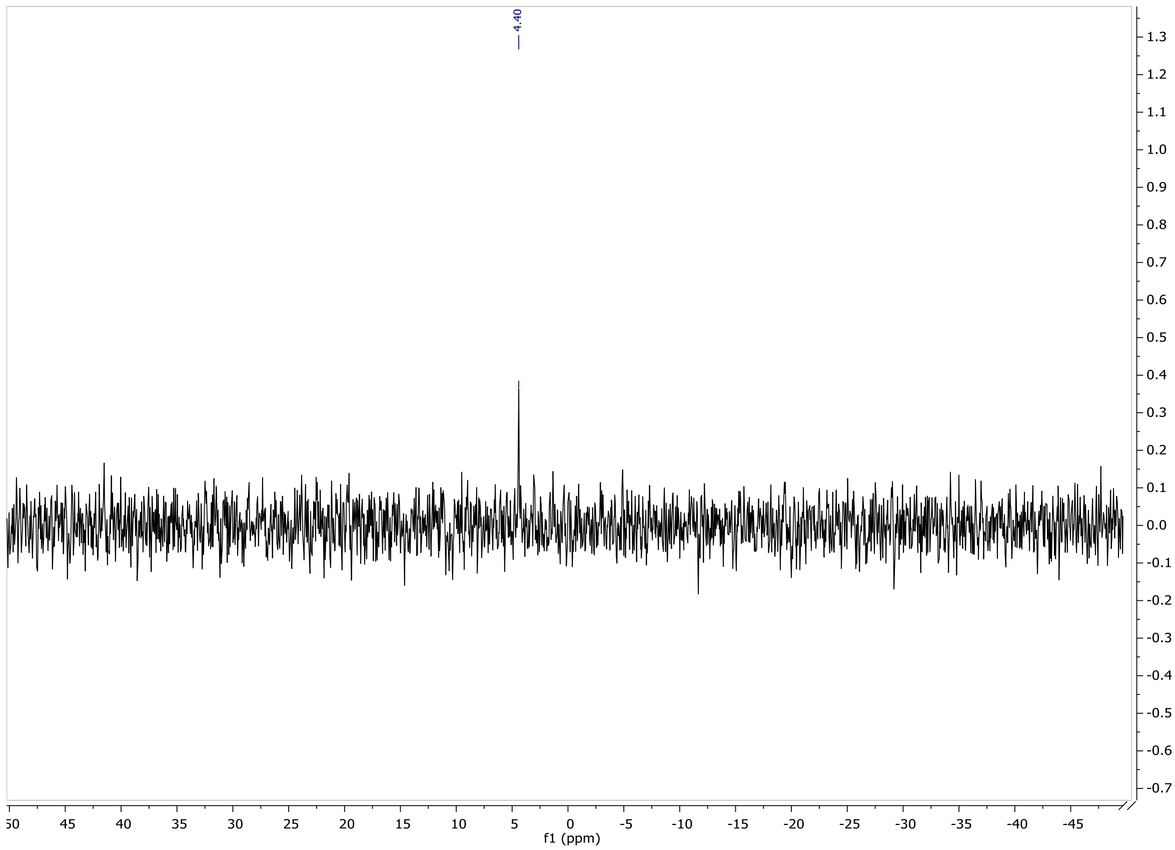

4.40
f1 (ppm)

1-(dimethyl(phenyl)silyl)-1,2-bis(4-fluorophenyl)-2-(4,4,5,5-tetramethyl-1,3,2-dioxaborolan-2-yl)hydrazine (11)

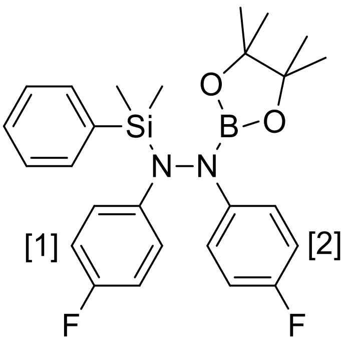

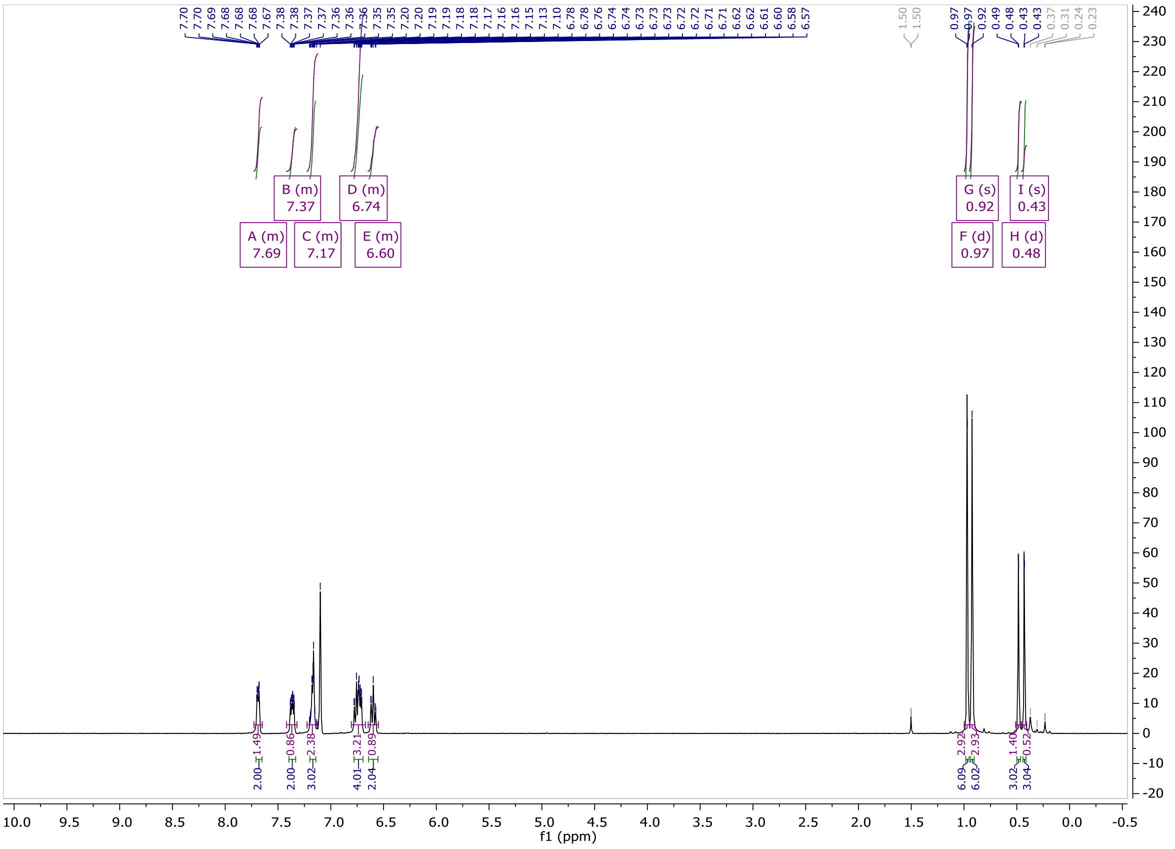

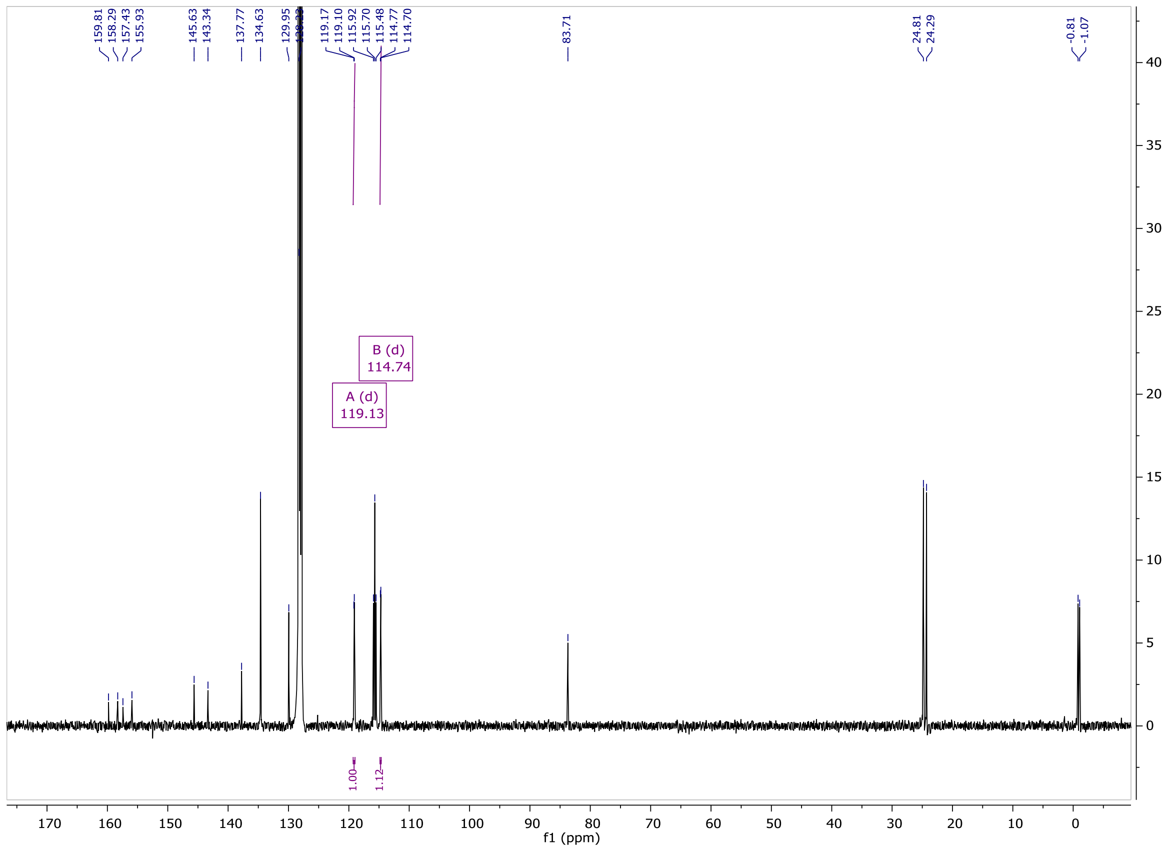
159.81
158.29
157.43
155.93
145.63
143.34
137.77
134.63
129.95
119.17
119.10
115.92
115.70
115.48
114.77
114.70
83.71
24.81
24.29
-0.81
-1.07
B (d)
114.74
A (d)
119.13
1.00
1.12
f1 (ppm)

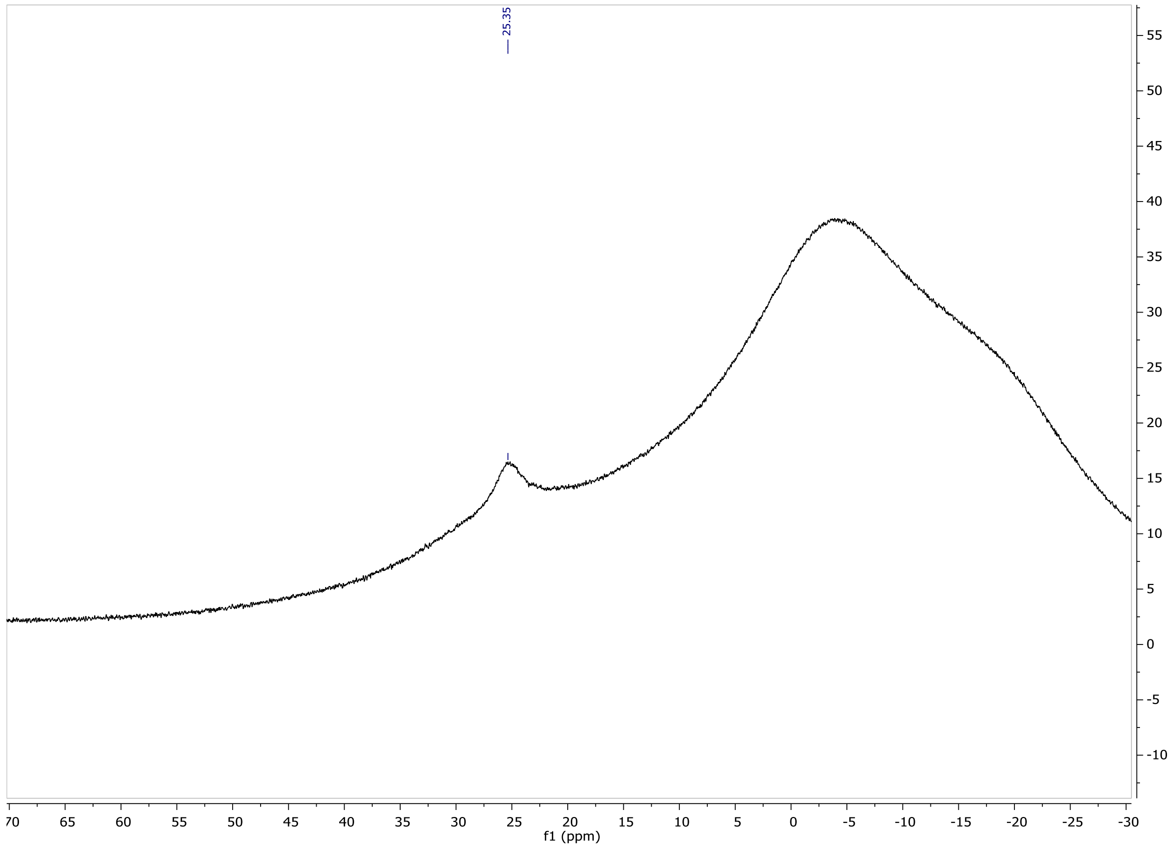
25.35
f1 (ppm)

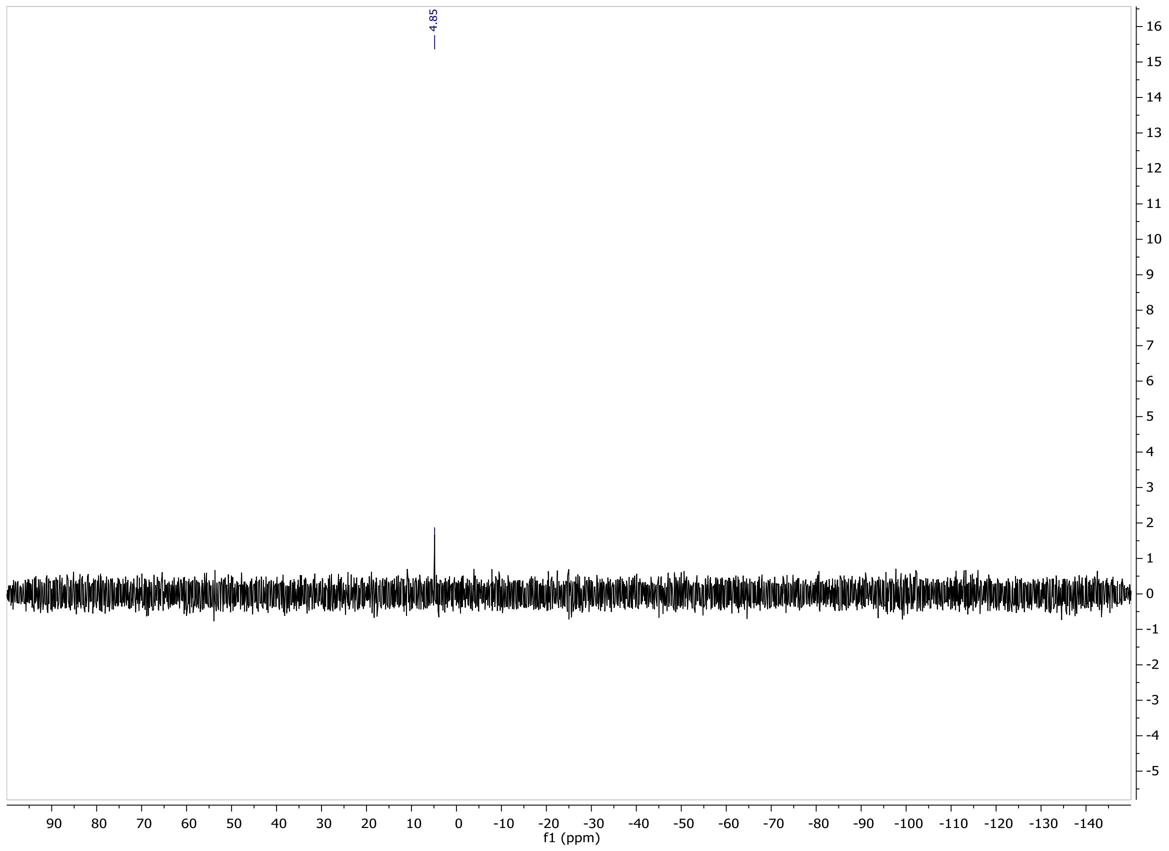

4.85
f1 (ppm)

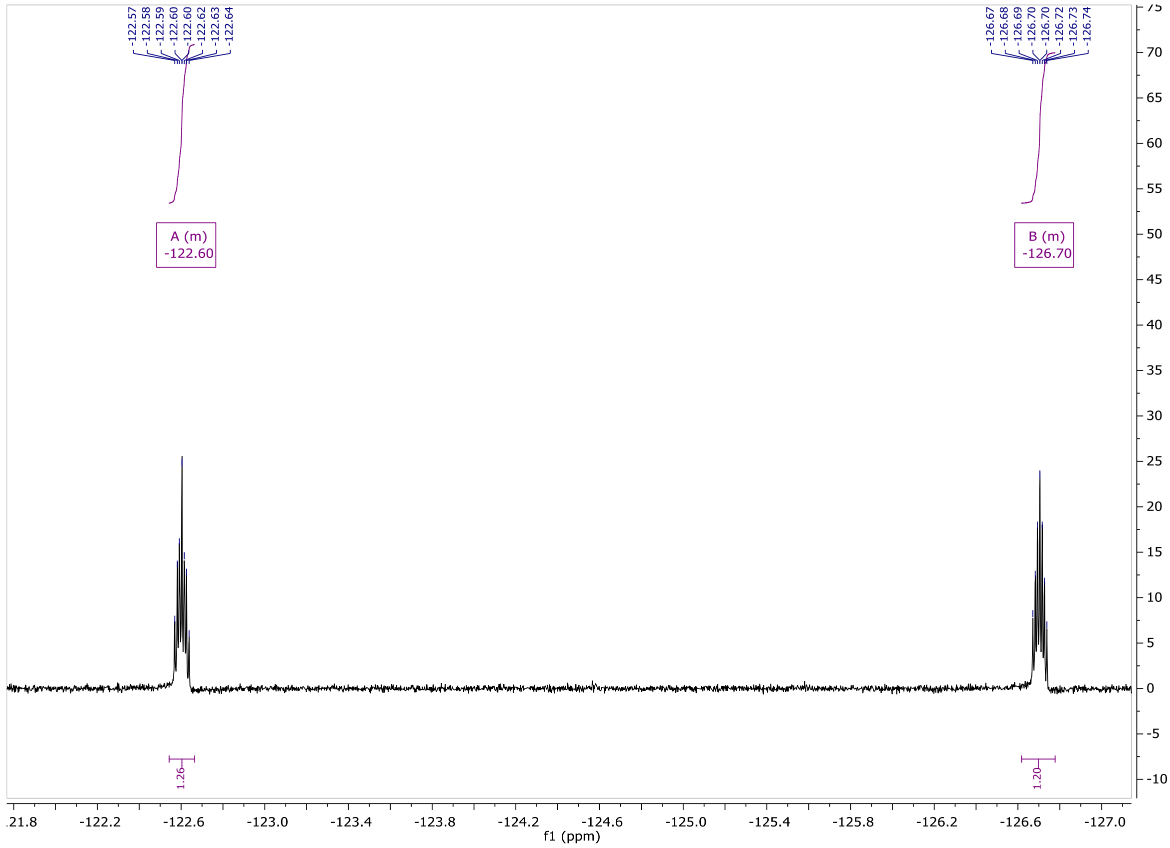

-122.57
-122.58
-122.59
-122.60
-122.60
-122.62
-122.63
-122.64
-126.67
-126.68
-126.69
-126.70
-126.70
-126.72
-126.73
-126.74
A (m)
-122.60
B (m)
-126.70
1.26
1.20
f1 (ppm)

1-(dimethyl(phenyl)silyl)-2-(4,4,5,5-tetramethyl-1,3,2-dioxaborolan-2-yl)-1,2-di-p-tolylhydrazine (12)

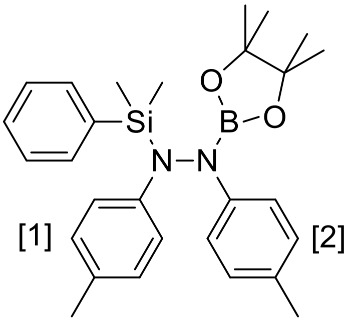

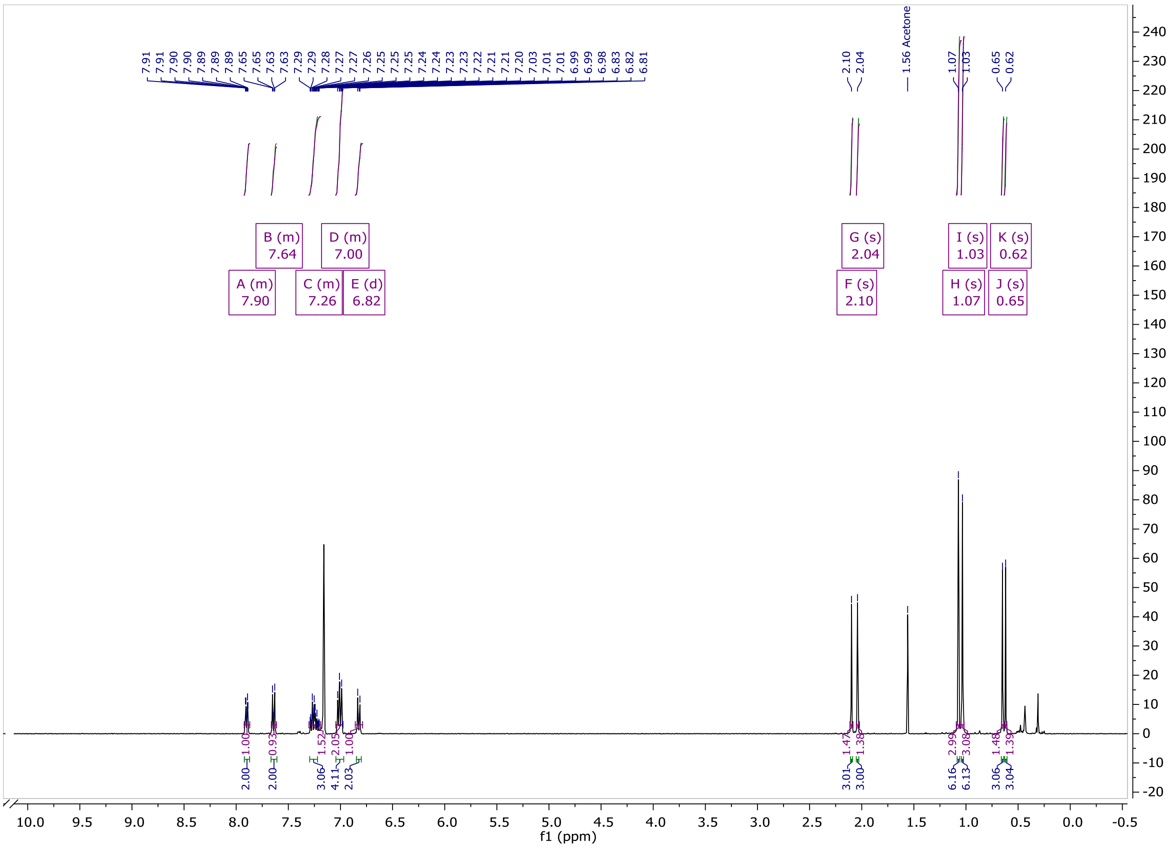

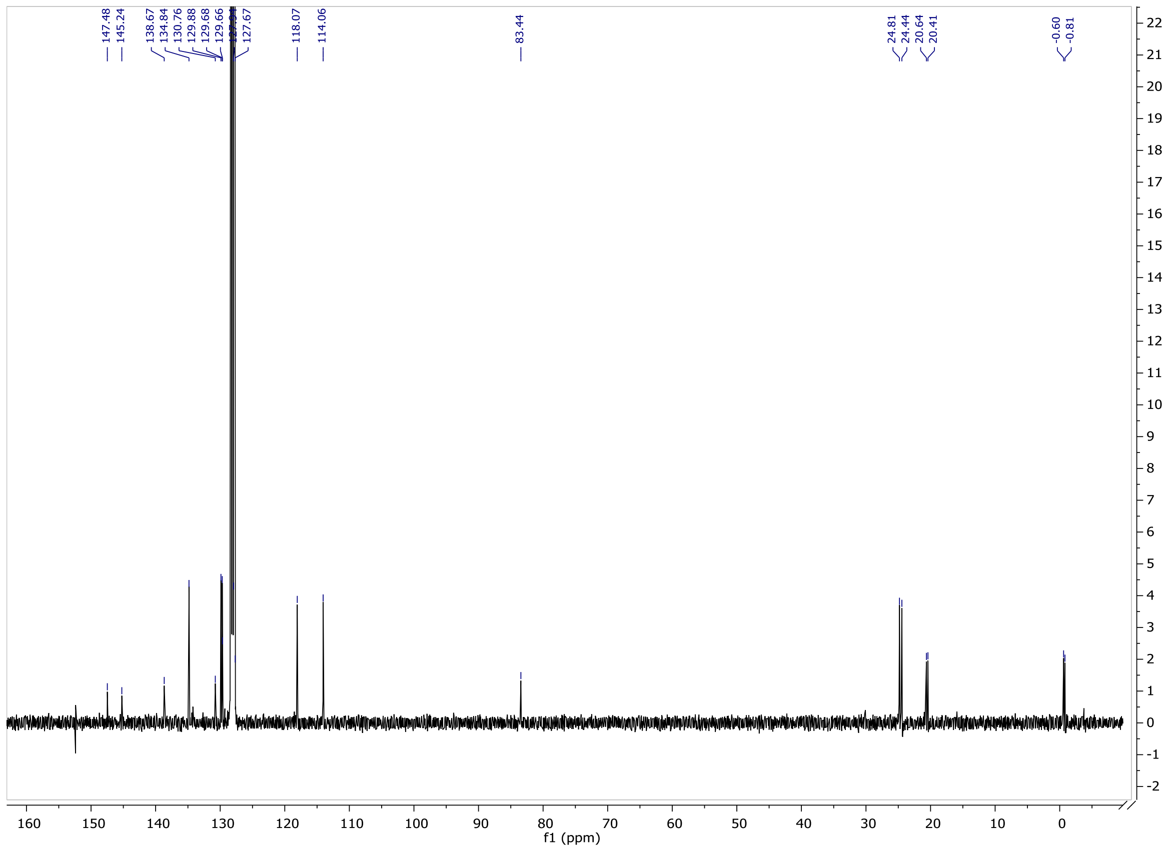
147.48
145.24
138.67
134.84
130.76
129.88
129.68
129.66
127.67
118.07
114.06
83.44
24.81
24.44
20.64
20.41
-0.60
-0.81
f1 (ppm)

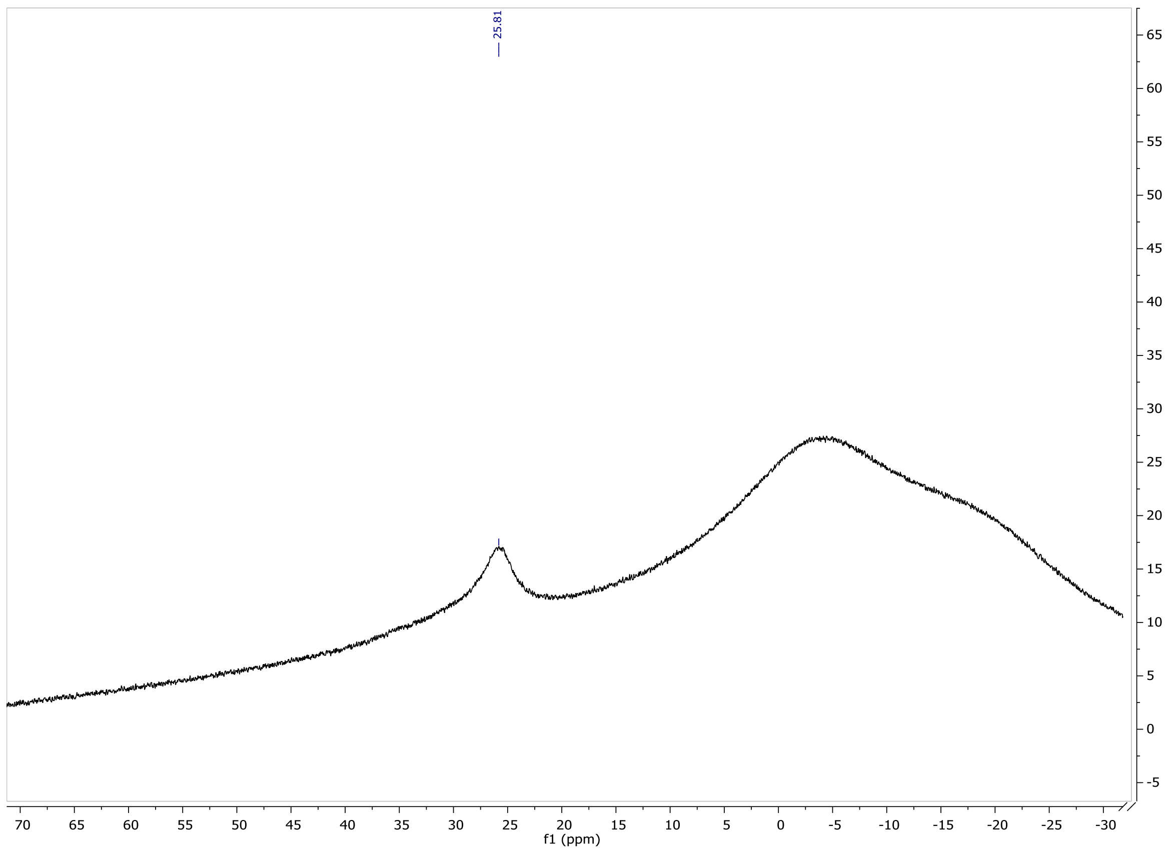
25.81
f1 (ppm)

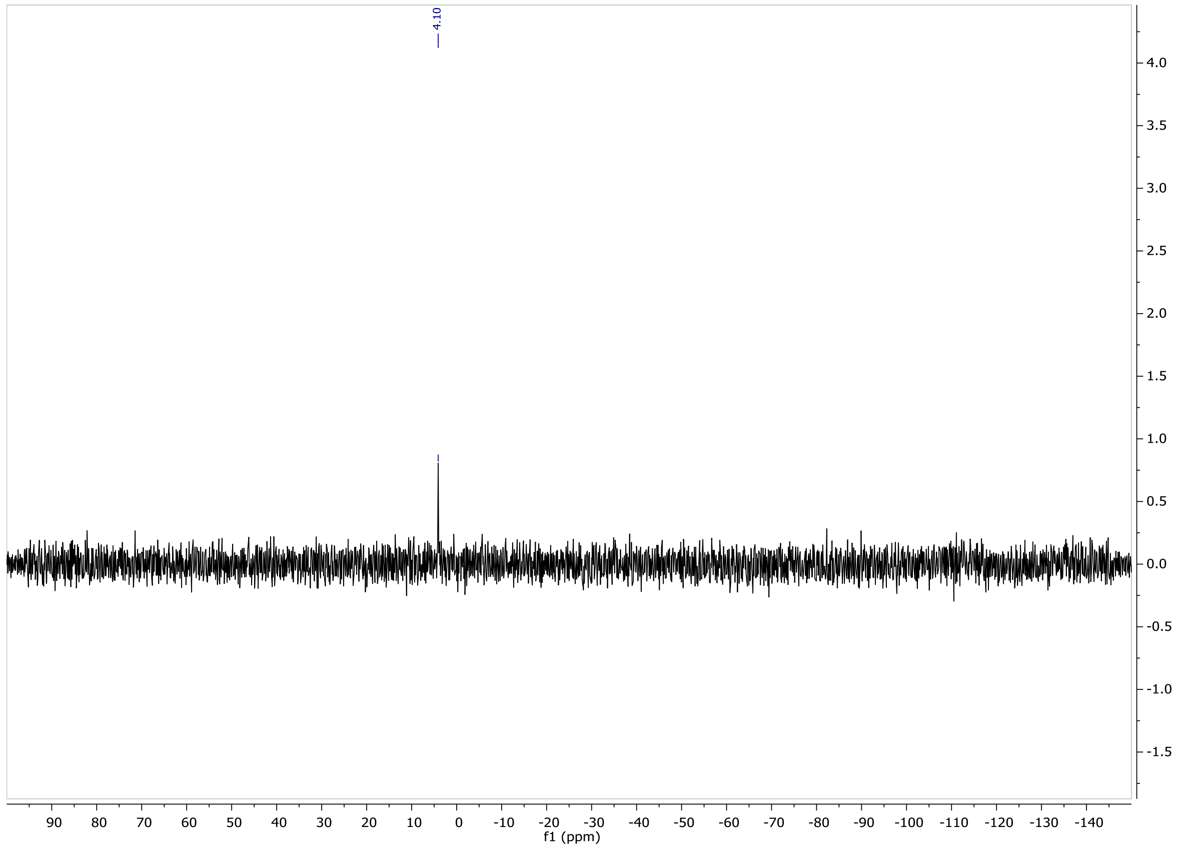
4.10
f1 (ppm)

Reaction of 1-(4-methoxyphenyl)-2-phenyldiazene and PhMe2SiBpin (Crude)

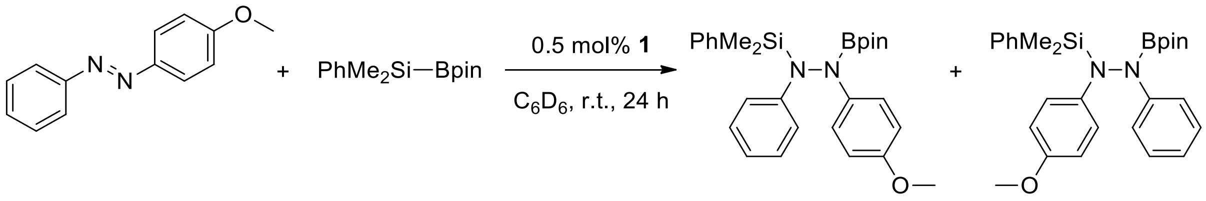

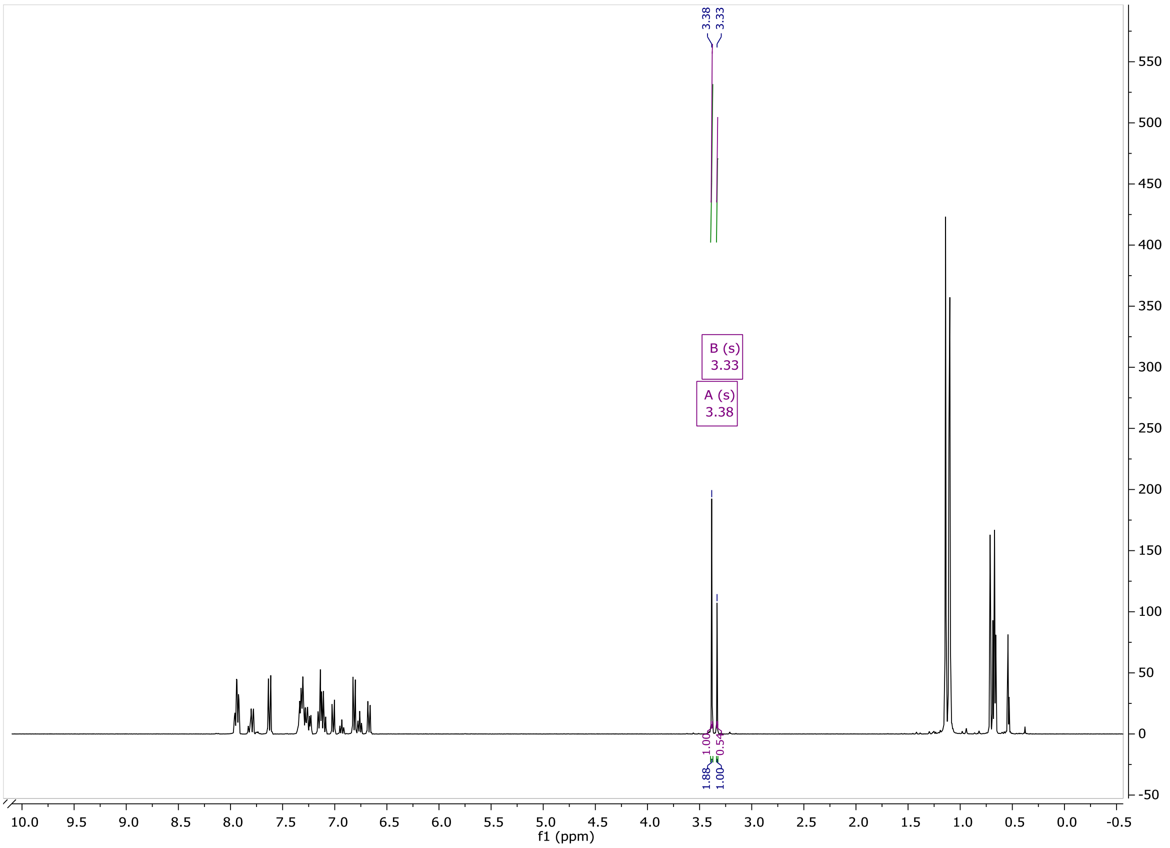

1,2-diphenylhydrazine

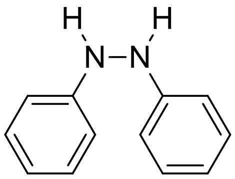

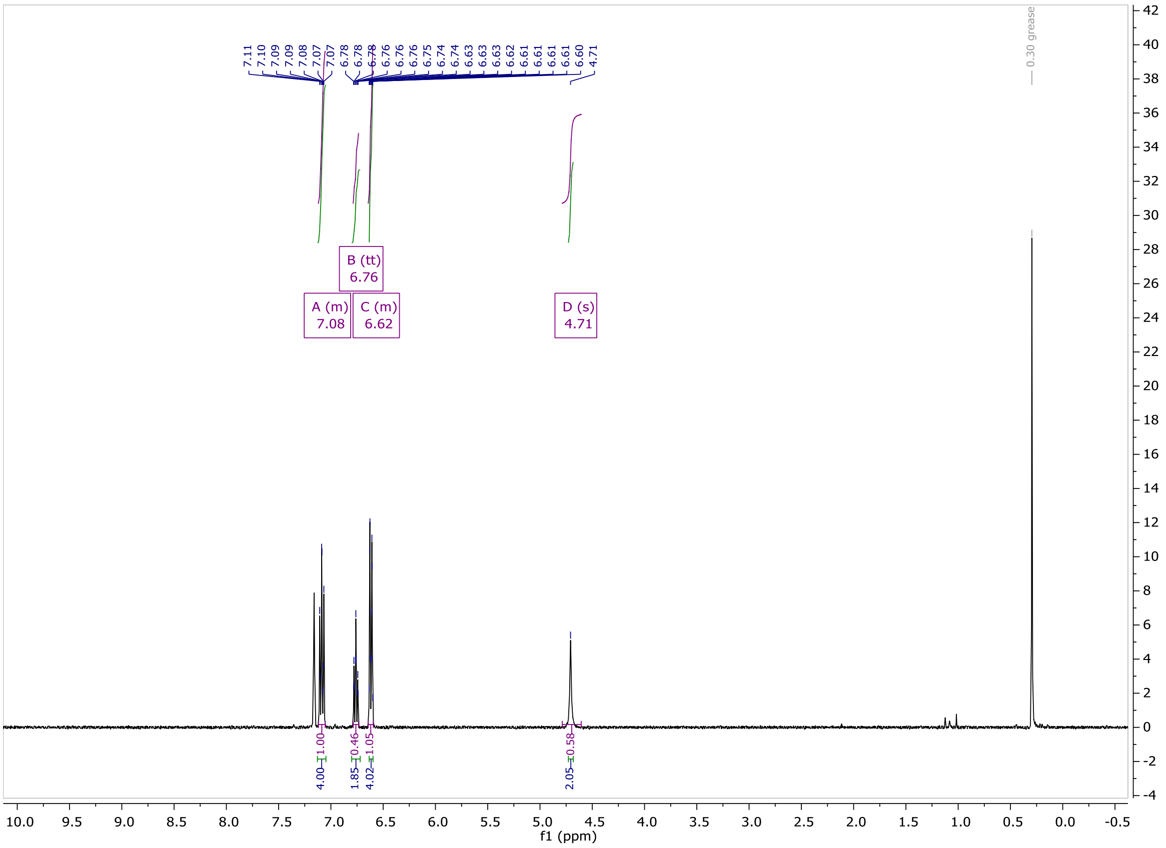

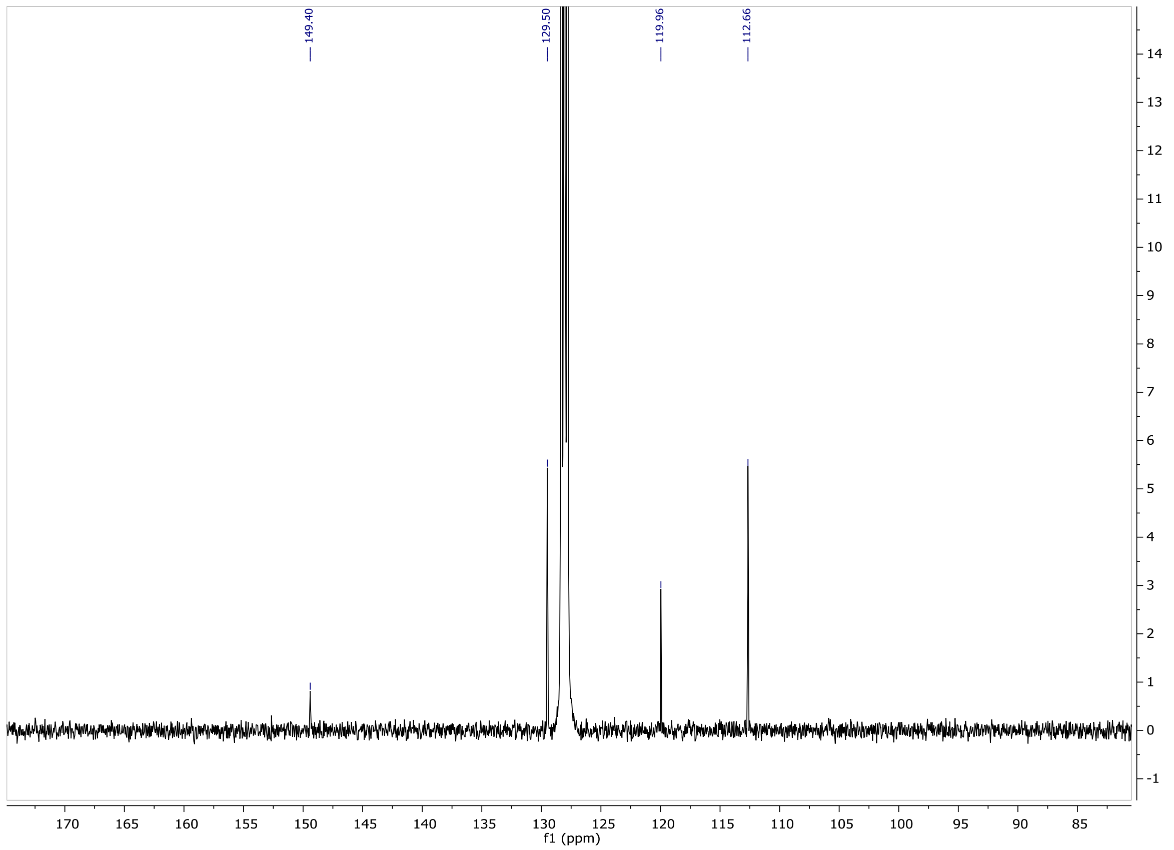
149.40
129.50
119.96
112.66
f1 (ppm)

X-ray Crystallography Data

The crystal data of **2** was collected on a BRUKER X8-APEX II diffractometer with a CCD area detector and multi-layer mirror monochromated Mo_{Ka} radiation. The structure was solved using intrinsic phasing method (SHELXT[4]), refined with the SHELXL program,[5] and expanded using Fourier techniques. All non-hydrogen atoms were refined anisotropically. Hydrogen atoms were included in structure factors calculations. All hydrogen atoms were assigned to idealised geometric positions. Single crystal X-ray diffraction data for **4**, **9** and **11** were collected at the University of Sussex on an Agilent Technologies Xcalibur Gemini Ultra diffractometer (λCuKα = 1.54184) equipped with a Eos CCD area detector. The data were collected at 173 K using an Oxford Cryosystems Cobra low temperature device. Data were processed using CrysAlisPro, and the unit cell parameters were refined against all data. Semi empirical absorption corrections were carried out using the MULTI-SCAN program.[6] The structures were solved by using an intrinsic phasing method (SHELXT[4]), and refined F0 by full matrix least squares refinement using SHELXL-2013,[7] within OLEX2.[8] All non-hydrogen atoms were refined with anisotropic displacement parameters. Hydrogen atoms were added at calculated positions and refined using riding models with isotropic displacement parameters based on the equivalent isotropic displacement parameter (Ueq) of the parent atoms. The cif files for **2**, **4**, **9** and **11** have been deposited with the CCDC and have been given the deposition numbers 1501645, 1501646, 1501647 and 1501648 respectively.

Crystal Structure Information for **2**

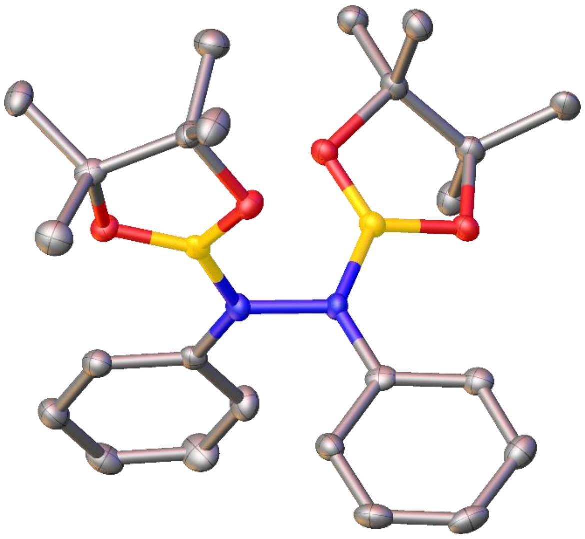

Table S1 Crystal data and structure refinement for 2.

Empirical formula	$C_{24}H_{34}B_2N_2O_4$
Formula weight	436.15
Temperature/K	103.15
Crystal system	orthorhombic
Space group	$P2_12_12_1$
a/Å	11.248(3)
b/Å	12.019(6)
c/Å	17.861(4)
α/°	90
β/°	90
γ/°	90
Volume/Å^3	2414.6(15)
Z	4

ρ_{calc}g/cm^3	1.200
μ/mm^{-1}	0.079
F(000)	936.0
Crystal size/mm^3	0.129 × 0.116 × 0.105
Radiation	MoKα (λ = 0.71073)
2Θ range for data collection/°	4.084 to 52.744
Index ranges	-14 ≤ h ≤ 9, -15 ≤ k ≤ 10, -14 ≤ l ≤ 22
Reflections collected	10281
Independent reflections	4865 [R_{int} = 0.0530, R_{sigma} = 0.0865]
Data/restraints/parameters	4865/522/383
Goodness-of-fit on F^2	1.017
Final R indexes [I>=2σ (I)]	R_1 = 0.0483, wR_2 = 0.0890
Final R indexes [all data]	R_1 = 0.0756, wR_2 = 0.0982
Largest diff. peak/hole / e Å^{-3}	0.16/-0.21
Flack parameter	-0.2(10)

Table S2 Fractional Atomic Coordinates (×10^4) and Equivalent Isotropic Displacement Parameters (Å^2×10^3) for 2. U_{eq} is defined as 1/3 of of the trace of the orthogonalised U_{IJ} tensor.

**Atom**	***x***	***y***	***z***	**U(eq)**
O1	1114.0(17)	7254.9(18)	7147(1)	16.7(5)
N1	2296(2)	6083(2)	6285.0(12)	16.3(6)
C1	2720(3)	6894(3)	5769.0(15)	15.2(7)
B1	1542(3)	6249(3)	6921.5(17)	15.1(8)

O2	1185.4(17)	5370.2(18)	7358.4(10)	18.3(5)
N2	2790(2)	5001(2)	6218.1(12)	15.4(6)
C2	3577(3)	6625(3)	5235.9(16)	23.1(8)
C3	3974(3)	7422(3)	4736.0(16)	30.7(9)
C4	3528(3)	8491(3)	4748.4(17)	24.1(8)
C5	2680(3)	8758(3)	5276.1(15)	23.7(8)
C6	2281(3)	7976(3)	5782.7(16)	19.2(8)
C7	2178(3)	4256(3)	5737.4(15)	16.7(7)
C8	2568(3)	3158(3)	5657.3(15)	19.4(8)
C9	1964(3)	2444(3)	5183.3(16)	23.2(8)
C10	963(3)	2790(3)	4790.9(15)	24.3(8)
C11	589(3)	3870(3)	4870.3(15)	24.6(8)
C12	1192(3)	4605(3)	5331.1(14)	19.4(8)
C13	296(3)	7045(3)	7766.4(15)	18.2(8)
C14	669(3)	5864(3)	8035.9(15)	19.2(8)
C15	-954(3)	7096(3)	7438.6(16)	26.2(9)
C16	447(3)	7954(3)	8344.6(16)	23.0(8)
C17	-351(3)	5138(3)	8294.8(17)	27.4(8)
C18	1651(3)	5865(3)	8619.8(15)	26.6(9)
B3	3900(50)	4890(60)	6710(40)	15.8(18)
O3	4130(13)	5519(14)	7265(8)	16.0(17)
C19	5129(14)	4933(16)	7651(9)	18.4(16)
C20	5680(15)	4239(16)	7020(9)	18.7(16)

O4	4585(15)	3948(15)	6571(8)	16.5(17)
C21	5890(50)	5700(40)	8060(20)	21(4)
C22	6460(18)	4836(19)	6470(11)	24(3)
C23	4518(19)	4314(18)	8283(11)	23(3)
C24	6130(50)	3140(50)	7330(30)	20(4)
B2	3799(12)	4793(13)	6665(9)	15.1(14)
O13	4367(3)	5679(3)	7020.2(17)	16.4(7)
C119	5518(3)	5237(4)	7266(2)	18.6(9)
C120	5237(3)	3997(4)	7381(2)	19.1(9)
O14	4347(3)	3793(3)	6789.4(18)	17.1(8)
C121	5917(11)	5875(9)	7947(5)	19.4(15)
C122	6396(4)	5437(4)	6626(2)	24.5(11)
C123	4633(4)	3759(4)	8129(2)	24.8(11)
C124	6296(10)	3229(11)	7254(6)	23.1(19)

Table S3 Anisotropic Displacement Parameters ($Å^2×10^3$) for 2. The Anisotropic displacement factor exponent takes the form: $-2\pi^2[h^2a^{*2}U_{11}+2hka^*b^*U_{12}+…]$.

**Atom**	**U_{11}**	**U_{22}**	**U_{33}**	**U_{23}**	**U_{13}**	**U_{12}**
O1	15.2(11)	19.4(14)	15.5(9)	-0.7(9)	3.7(9)	0.1(10)
N1	14.2(13)	17.2(17)	17.6(11)	0.1(12)	0.8(10)	3.9(13)
C1	11.7(15)	19(2)	14.7(14)	0.4(13)	-2.1(12)	-0.3(15)
B1	11.1(17)	18(2)	16.5(16)	-0.3(15)	-4.6(13)	0.1(17)
O2	20.6(11)	16.8(14)	17.4(10)	-1.6(9)	4.0(9)	-1.3(10)
N2	14.7(13)	15.3(17)	16.2(11)	-1.8(11)	0.3(10)	4.3(12)

C2	19.9(17)	24(2)	25.9(16)	1.0(15)	6.7(13)	4.6(16)
C3	25.2(19)	40(3)	26.8(16)	4.1(17)	12.0(15)	5.0(18)
C4	19.5(17)	30(2)	23.3(16)	10.1(15)	-0.6(14)	-4.7(16)
C5	25.2(18)	23(2)	22.8(15)	3.5(15)	-0.9(14)	1.6(17)
C6	17.1(16)	23(2)	17.1(14)	0.0(14)	1.2(13)	1.7(16)
C7	13.1(15)	25(2)	12.3(13)	0.2(13)	5.1(12)	-1.5(15)
C8	15.5(17)	26(2)	16.3(15)	0.6(14)	2.9(12)	1.2(16)
C9	22.9(18)	23(2)	23.3(15)	-4.1(16)	6.3(14)	-3.4(15)
C10	23.1(18)	33(2)	16.9(14)	-7.3(15)	2.4(14)	-10.3(17)
C11	19.9(17)	38(3)	15.5(14)	2.2(16)	-2.7(13)	-1.0(17)
C12	19.2(16)	22(2)	16.9(14)	1.2(13)	0.5(13)	4.3(15)
C13	13.8(16)	25(2)	16.0(14)	-0.7(14)	4.1(12)	-3.4(15)
C14	22.2(17)	20(2)	15.4(14)	-1.2(13)	3.2(12)	1.1(15)
C15	17.3(18)	34(3)	27.7(16)	2.7(16)	3.4(14)	-2.6(17)
C16	26.6(18)	19(2)	23.3(15)	-1.5(15)	10.0(14)	0.7(17)
C17	32(2)	23(2)	27.7(16)	-0.3(16)	12.0(15)	-5.5(18)
C18	27.8(19)	31(3)	20.9(15)	1.3(15)	-1.6(14)	2.6(18)
B3	13(3)	18(3)	16(3)	1(3)	-1(3)	1(3)
O3	12(3)	18(3)	17(3)	1(3)	-2(3)	2(3)
C19	15(3)	20(3)	20(3)	0(3)	-3(2)	2(3)
C20	15(3)	21(3)	20(3)	0(3)	-3(2)	0(3)
O4	13(3)	19(3)	17(3)	1(3)	-2(3)	2(3)
C21	20(6)	23(7)	21(7)	-1(6)	-3(6)	0(6)

C22	21(6)	24(6)	27(6)	-3(5)	5(5)	-1(6)
C23	23(6)	24(6)	24(5)	2(5)	-1(5)	4(6)
C24	14(7)	23(6)	24(7)	-2(5)	-7(6)	0(5)
B2	13(3)	17(3)	15(2)	-1(2)	3(2)	0(2)
O13	14.1(15)	17.7(16)	17.5(15)	-0.4(13)	-2.6(12)	2.9(13)
C119	15.0(16)	21.1(19)	19.8(17)	-0.7(15)	-3.6(14)	1.4(16)
C120	14.6(17)	22(2)	20.7(17)	0.7(16)	-4.6(14)	0.2(16)
O14	14.1(16)	18.2(16)	19.0(16)	-0.5(13)	-3.4(12)	1.0(13)
C121	19(2)	22(4)	17(3)	5(2)	-3(2)	-2(3)
C122	21(2)	26(3)	26(2)	0(2)	1.6(17)	-3(2)
C123	21(2)	31(3)	23(2)	7(2)	0.2(17)	2(2)
C124	18(4)	24(4)	27(3)	0(3)	-1(2)	2(3)

Table S4 Bond Lengths for 2.

Atom	Atom	Length/Å	Atom	Atom	Length/Å
O1	B1	1.362(4)	C13	C15	1.525(4)
O1	C13	1.461(3)	C13	C16	1.513(4)
N1	C1	1.423(4)	C14	C17	1.513(4)
N1	B1	1.433(4)	C14	C18	1.519(4)
N1	N2	1.419(4)	B3	O3	1.28(6)
C1	C2	1.393(4)	B3	O4	1.39(7)
C1	C6	1.391(4)	O3	C19	1.49(2)
B1	O2	1.373(4)	C19	C20	1.53(2)
O2	C14	1.468(3)	C19	C21	1.45(5)

N2	C7	1.419(4)	C19	C23	1.52(3)
N2	B3	1.54(6)	C20	O4	1.51(2)
N2	B2	1.410(14)	C20	C22	1.50(3)
C2	C3	1.384(4)	C20	C24	1.52(6)
C3	C4	1.379(5)	B2	O13	1.394(13)
C4	C5	1.379(4)	B2	O14	1.369(16)
C5	C6	1.380(4)	O13	C119	1.467(5)
C7	C8	1.398(5)	C119	C120	1.537(6)
C7	C12	1.390(4)	C119	C121	1.506(11)
C8	C9	1.384(4)	C119	C122	1.531(6)
C9	C10	1.390(4)	C120	O14	1.476(5)
C10	C11	1.371(5)	C120	C123	1.527(6)
C11	C12	1.385(4)	C120	C124	1.523(13)
C13	C14	1.556(5)			

Table S5 Bond Angles for 2.

Atom	Atom	Atom	Angle/°	Atom	Atom	Atom	Angle/°
B1	O1	C13	107.0(2)	C17	C14	C13	114.6(3)
C1	N1	B1	128.1(3)	C17	C14	C18	110.0(3)
N2	N1	C1	116.2(2)	C18	C14	C13	114.1(3)
N2	N1	B1	115.2(2)	O3	B3	N2	124(5)
C2	C1	N1	121.0(3)	O3	B3	O4	121(5)
C6	C1	N1	120.6(3)	O4	B3	N2	115(4)
C6	C1	C2	118.4(3)	B3	O3	C19	103(3)

O1	B1	N1	124.6(3)	O3	C19	C20	102.8(12)
O1	B1	O2	114.3(3)	O3	C19	C23	103.5(14)
O2	B1	N1	121.1(3)	C21	C19	O3	112(3)
B1	O2	C14	105.8(2)	C21	C19	C20	118(3)
N1	N2	B3	111(3)	C21	C19	C23	102(2)
C7	N2	N1	116.1(2)	C23	C19	C20	117.6(18)
C7	N2	B3	133(3)	O4	C20	C19	100.8(13)
B2	N2	N1	115.5(6)	O4	C20	C24	105(3)
B2	N2	C7	128.4(6)	C22	C20	C19	117.2(18)
C3	C2	C1	120.2(3)	C22	C20	O4	103.8(14)
C4	C3	C2	121.1(3)	C22	C20	C24	117(3)
C5	C4	C3	118.6(3)	C24	C20	C19	110(2)
C4	C5	C6	121.0(3)	B3	O4	C20	100(3)
C5	C6	C1	120.6(3)	O13	B2	N2	119.4(12)
C8	C7	N2	120.4(3)	O14	B2	N2	127.6(9)
C12	C7	N2	120.8(3)	O14	B2	O13	113.0(9)
C12	C7	C8	118.8(3)	B2	O13	C119	105.3(7)
C9	C8	C7	119.6(3)	O13	C119	C120	102.1(3)
C8	C9	C10	121.3(3)	O13	C119	C121	108.7(6)
C11	C10	C9	118.7(3)	O13	C119	C122	106.8(3)
C10	C11	C12	121.0(3)	C121	C119	C120	116.6(4)
C11	C12	C7	120.6(3)	C121	C119	C122	109.3(5)
O1	C13	C14	102.9(2)	C122	C119	C120	112.6(4)

O1	C13	C15	106.4(2)	O14	C120	C119	101.8(3)
O1	C13	C16	108.8(2)	O14	C120	C123	107.1(3)
C15	C13	C14	113.8(3)	O14	C120	C124	108.9(6)
C16	C13	C14	114.7(2)	C123	C120	C119	112.9(4)
C16	C13	C15	109.6(3)	C124	C120	C119	114.0(5)
O2	C14	C13	102.7(2)	C124	C120	C123	111.4(5)
O2	C14	C17	108.6(3)	B2	O14	C120	106.0(6)
O2	C14	C18	106.2(2)				

Table S6 Torsion Angles for 2.

A	B	C	D	Angle/°	A	B	C	D	Angle/°
O1	B1	O2	C14	-12.8(3)	C8	C9	C10	C11	1.2(4)
O1	C13	C14	O2	-26.3(3)	C9	C10	C11	C12	0.1(4)
O1	C13	C14	C17	-143.8(2)	C10	C11	C12	C7	-1.5(4)
O1	C13	C14	C18	88.1(3)	C12	C7	C8	C9	-0.5(4)
N1	C1	C2	C3	179.7(3)	C13	O1	B1	N1	174.6(3)
N1	C1	C6	C5	-179.2(3)	C13	O1	B1	O2	-5.2(3)
N1	B1	O2	C14	167.5(3)	C15	C13	C14	O2	88.4(3)
N1	N2	C7	C8	-177.5(2)	C15	C13	C14	C17	-29.1(3)
N1	N2	C7	C12	3.3(4)	C15	C13	C14	C18	-157.1(2)
N1	N2	B3	O3	21(7)	C16	C13	C14	O2	-144.2(2)
N1	N2	B3	O4	-168(4)	C16	C13	C14	C17	98.3(3)
N1	N2	B2	O13	-11.8(14)	C16	C13	C14	C18	-29.8(3)
N1	N2	B2	O14	170.1(11)	B3	N2	C7	C8	2(4)

C1	N1	B1	O1	-1.3(5)	B3	N2	C7	C12	-177(4)
C1	N1	B1	O2	178.4(3)	B3	O3	C19	C20	24(4)
C1	N1	N2	C7	-90.6(3)	B3	O3	C19	C21	152(4)
C1	N1	N2	B3	90(3)	B3	O3	C19	C23	-99(4)
C1	N1	N2	B2	92.0(8)	O3	B3	O4	C20	-19(6)
C1	C2	C3	C4	-0.6(5)	O3	C19	C20	O4	-34.4(17)
B1	O1	C13	C14	19.6(3)	O3	C19	C20	C22	77.4(18)
B1	O1	C13	C15	-100.4(3)	O3	C19	C20	C24	-145(3)
B1	O1	C13	C16	141.6(3)	C19	C20	O4	B3	31(3)
B1	N1	C1	C2	171.3(3)	O4	B3	O3	C19	-3(6)
B1	N1	C1	C6	-9.1(4)	C21	C19	C20	O4	-158(2)
B1	N1	N2	C7	96.6(3)	C21	C19	C20	C22	-46(3)
B1	N1	N2	B3	-83(3)	C21	C19	C20	C24	91(4)
B1	N1	N2	B2	-80.8(8)	C22	C20	O4	B3	-91(3)
B1	O2	C14	C13	23.7(3)	C23	C19	C20	O4	78.5(18)
B1	O2	C14	C17	145.5(3)	C23	C19	C20	C22	-169.7(16)
B1	O2	C14	C18	-96.3(3)	C23	C19	C20	C24	-32(3)
N2	N1	C1	C2	-0.4(4)	C24	C20	O4	B3	146(4)
N2	N1	C1	C6	179.2(2)	B2	N2	C7	C8	-0.5(9)
N2	N1	B1	O1	170.5(3)	B2	N2	C7	C12	-179.7(9)
N2	N1	B1	O2	-9.8(4)	B2	O13	C119	C120	-28.4(8)
N2	C7	C8	C9	-179.7(2)	B2	O13	C119	C121	-152.2(8)
N2	C7	C12	C11	-179.1(3)	B2	O13	C119	C122	89.9(8)

N2	B3	O3	C19	168(5)	O13	B2	O14	C120	10.2(12)
N2	B3	O4	C20	169(4)	O13	C119	C120	O14	33.7(4)
N2	B2	O13	C119	-165.8(10)	O13	C119	C120	C123	-80.8(4)
N2	B2	O14	C120	-171.7(12)	O13	C119	C120	C124	150.8(6)
C2	C1	C6	C5	0.5(4)	C119	C120	O14	B2	-27.1(7)
C2	C3	C4	C5	0.6(5)	O14	B2	O13	C119	12.5(12)
C3	C4	C5	C6	-0.1(5)	C121	C119	C120	O14	152.0(6)
C4	C5	C6	C1	-0.5(5)	C121	C119	C120	C123	37.5(7)
C6	C1	C2	C3	0.0(4)	C121	C119	C120	C124	-90.9(8)
C7	N2	B3	O3	-158(4)	C122	C119	C120	O14	-80.5(4)
C7	N2	B3	O4	13(7)	C122	C119	C120	C123	165.1(3)
C7	N2	B2	O13	171.2(7)	C122	C119	C120	C124	36.6(7)
C7	N2	B2	O14	-6.8(19)	C123	C120	O14	B2	91.6(8)
C7	C8	C9	C10	-0.9(4)	C124	C120	O14	B2	-147.9(8)
C8	C7	C12	C11	1.7(4)					

Table S7 Hydrogen Atom Coordinates (Å×10^4) and Isotropic Displacement Parameters (Å^2×10^3) for 2.

Atom	*x*	*y*	*z*	U(eq)
H2	3890	5891	5216	28
H3	4564	7230	4378	37
H4	3800	9032	4400	29
H5	2366	9491	5291	28
H6	1700	8178	6144	23

H8	3243	2903	5927	23
H9	2239	1701	5125	28
H10	547	2290	4474	29
H11	-94	4117	4605	30
H12	930	5354	5370	23
H15A	-1539	7014	7841	39
H15B	-1057	6493	7075	39
H15C	-1070	7814	7189	39
H16A	-23	7773	8790	35
H16B	174	8663	8136	35
H16C	1288	8015	8482	35
H17A	-774	5510	8704	41
H17B	-40	4423	8471	41
H17C	-899	5012	7877	41
H18A	1340	6154	9094	40
H18B	2307	6337	8448	40
H18C	1941	5103	8694	40
H21A	6553	5293	8281	32
H21B	5429	6070	8451	32
H21C	6200	6264	7710	32
H22A	6676	4329	6063	36
H22B	7183	5093	6723	36
H22C	6032	5477	6265	36

H23A	5116	3939	8591	35
H23B	3971	3761	8073	35
H23C	4072	4842	8592	35
H24A	6411	2670	6915	30
H24B	5490	2759	7596	30
H24C	6794	3279	7675	30
H12A	6628	5523	8158	29
H12B	5281	5875	8322	29
H12C	6102	6643	7804	29
H12D	7183	5159	6769	37
H12E	6446	6236	6521	37
H12F	6123	5045	6177	37
H12G	4307	3002	8125	37
H12H	3988	4294	8209	37
H12I	5216	3827	8535	37
H12J	6071	2463	7378	35
H12K	6957	3463	7575	35
H12L	6541	3269	6728	35

Crystal Structure data for 4

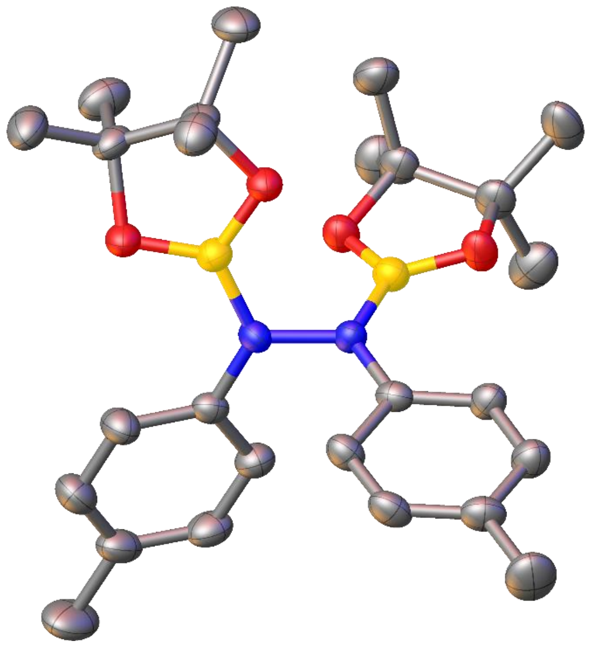

Table S8 Crystal data and structure refinement for 4.

Empirical formula	$C_{26}H_{38}B_2N_2O_4$
Formula weight	464.20
Temperature/K	173
Crystal system	orthorhombic
Space group	$P2_12_12_1$
a/Å	11.3842(5)
b/Å	12.2242(5)
c/Å	19.0048(8)
α/°	90
β/°	90
γ/°	90
Volume/Å^3	2644.75(19)
Z	4

ρ_{calc}g/cm^3	1.166
μ/mm^{-1}	0.606
F(000)	1000.0
Crystal size/mm^3	0.1 × 0.1 × 0.1
Radiation	CuKα (λ = 1.54184)
2Θ range for data collection/°	8.6 to 142.618
Index ranges	-10 ≤ h ≤ 13, -15 ≤ k ≤ 13, -23 ≤ l ≤ 19
Reflections collected	14413
Independent reflections	5038 [R_{int} = 0.0599, R_{sigma} = 0.0593]
Data/restraints/parameters	5038/0/317
Goodness-of-fit on F^2	0.970
Final R indexes [I>=2σ (I)]	R_1 = 0.0503, wR_2 = 0.1226
Final R indexes [all data]	R_1 = 0.0681, wR_2 = 0.1288
Largest diff. peak/hole / e Å^{-3}	0.21/-0.21
Flack parameter	0.1(2)

Table S9 Fractional Atomic Coordinates (×10^4) and Equivalent Isotropic Displacement Parameters (Å^2×10^3) for 4. U_{eq} is defined as 1/3 of of the trace of the orthogonalised U_{IJ} tensor.

Atom	*x*	*Y*	*z*	U(eq)
O001	-4065.2(18)	-4837.4(16)	-5013.9(11)	34.0(5)
O002	-1272.6(19)	-5008.8(15)	-5013.6(11)	35.5(5)
O003	-4323.5(19)	-6564.8(16)	-4566.2(11)	36.2(5)
O004	-1226(2)	-3151.7(16)	-4845.9(11)	38.5(5)

N005	-2384(2)	-4287.2(18)	-4017.1(13)	29.0(5)
N006	-2938(2)	-5321.0(19)	-3961.5(12)	28.9(5)
C007	-2441(3)	-6076(2)	-3472.4(15)	30.0(6)
C008	-2660(2)	-3514(2)	-3485.5(15)	28.2(6)
C009	-3422(3)	-3777(2)	-2933.3(16)	35.2(7)
C00A	-2172(3)	-2470(2)	-3493.5(16)	33.7(6)
C00B	-5024(3)	-5332(3)	-5414.6(17)	37.0(7)
C00C	-1322(3)	-5925(3)	-3203.7(16)	36.9(7)
C00D	-3221(3)	-1962(3)	-2434.5(16)	39.1(7)
C00E	-3696(3)	-3002(3)	-2425.1(16)	40.1(7)
C00F	-4857(3)	-6574(2)	-5270.2(16)	36.7(7)
C00G	-3077(3)	-6981(3)	-3256.1(18)	40.0(7)
C00H	-2457(3)	-1716(3)	-2977.9(17)	39.3(7)
C00I	-696(3)	-4553(3)	-5635.4(17)	38.0(7)
C00J	-340(3)	-3387(3)	-5386.5(17)	39.7(7)
C00K	-847(3)	-6680(3)	-2739.8(18)	44.1(8)
C00L	-1618(3)	-4536(3)	-6215.7(17)	49.2(9)
C00M	-1464(3)	-7601(3)	-2531.7(18)	45.7(8)
C00N	-2587(4)	-7728(3)	-2792.9(19)	47.2(8)
C00O	-6164(3)	-4876(3)	-5123(2)	44.8(8)
B00P	-3764(3)	-5579(3)	-4503.9(17)	28.8(6)
C00Q	-4899(3)	-5006(3)	-6180.8(18)	49.2(9)
B00R	-1638(3)	-4134(3)	-4620.8(18)	31.6(7)

C00S	-5991(3)	-7216(3)	-5241(2)	51.5(9)
C00T	840(3)	-3341(3)	-5023(2)	55.8(9)
C00U	-3966(4)	-7118(3)	-5754(2)	53.2(9)
C00V	321(4)	-5288(3)	-5832(2)	53.9(9)
C00W	-415(4)	-2523(3)	-5954.1(19)	53.1(10)
C00X	-3511(4)	-1127(3)	-1875(2)	60.4(11)
C00Y	-938(5)	-8425(4)	-2034(2)	71.6(13)

Table S10 Anisotropic Displacement Parameters (Å^2×10^3) for 4. The Anisotropic displacement factor exponent takes the form: -2π^2[h^{2}a*^{2}U$_{11}$+2hka*b*U$_{12}$+…].

Atom	U11	U22	U33	U23	U13	U12
O001	36.3(11)	34.2(10)	31.5(11)	4.6(8)	-6.5(9)	-4.0(8)
O002	43.5(12)	33.3(9)	29.8(10)	-1.7(8)	8.3(9)	-2.2(8)
O003	42.7(11)	33.7(10)	32.2(11)	5.1(8)	-10.4(10)	-5.1(9)
O004	49.9(13)	34.2(10)	31.6(11)	-3.2(8)	17.4(10)	-5.3(9)
N005	32.2(12)	30.6(11)	24.2(11)	-0.5(9)	1.8(10)	-5.7(9)
N006	32.1(12)	30.2(11)	24.3(11)	4.7(9)	-1.1(10)	-4.6(9)
C007	33.7(15)	32.9(13)	23.3(12)	-1(1)	0.2(12)	1.9(11)
C008	26.6(13)	36.1(14)	22.0(12)	2.1(10)	-0.1(11)	0.3(11)
C009	39.1(16)	35.5(14)	31.1(15)	5.9(12)	6.5(13)	0.6(12)
C00A	34.0(15)	39.1(14)	27.9(13)	-2.8(12)	5.3(12)	-6.6(12)
C00B	37.7(15)	41.6(15)	31.7(15)	3.7(13)	-12.7(13)	-3.2(13)
C00C	37.0(16)	41.2(15)	32.4(15)	-1.0(12)	-5.3(13)	-2.1(13)
C00D	42.8(17)	48.4(17)	26.0(15)	-2.5(13)	3.2(13)	4.8(14)

C00E	47.4(18)	44.3(16)	28.7(15)	6.2(13)	9.1(14)	4.9(14)
C00F	40.1(16)	37.8(15)	32.3(16)	-3.6(12)	-12.7(13)	-2.0(13)
C00G	40.4(16)	42.7(17)	36.9(16)	10.9(13)	-6.3(14)	-5.4(13)
C00H	42.3(17)	41.0(15)	34.7(16)	-5.6(13)	3.6(14)	-6.1(14)
C00I	41.0(16)	43.1(16)	29.9(15)	-3.7(13)	11.1(13)	-4.1(13)
C00J	44.5(17)	44.9(16)	29.8(15)	-4.7(13)	15.0(14)	-6.7(14)
C00K	45.2(18)	49.8(18)	37.5(17)	-4.4(14)	-13.7(15)	4.0(15)
C00L	57(2)	59(2)	31.2(17)	-6.3(15)	4.3(16)	-11.3(18)
C00M	62(2)	44.7(17)	30.2(15)	2.0(14)	-11.8(16)	6.5(16)
C00N	59(2)	42.7(17)	39.7(17)	11.0(13)	-3.6(16)	-2.9(16)
C00O	38.7(17)	45.0(16)	50.7(19)	0.5(15)	-9.0(15)	1.6(13)
B00P	27.9(14)	32.1(15)	26.4(15)	-0.3(12)	-1.4(13)	-0.6(12)
C00Q	51(2)	59(2)	36.6(18)	8.6(16)	-13.4(16)	-2.7(17)
B00R	31.8(16)	35.2(15)	28.0(16)	-0.4(13)	3.1(14)	-2.3(12)
C00S	53(2)	45.1(17)	56(2)	3.3(15)	-18.5(18)	-10.1(16)
C00T	48(2)	72(2)	47(2)	-10.8(19)	10.0(18)	-14.7(18)
C00U	60(2)	53.2(19)	46(2)	-12.1(16)	-7.9(18)	10.3(17)
C00V	56(2)	54(2)	52(2)	-9.1(17)	19.0(18)	1.9(17)
C00W	73(3)	45.7(18)	40.1(19)	1.2(15)	24.1(19)	-6.7(18)
C00X	76(3)	59(2)	46(2)	-16.3(18)	17(2)	-1(2)
C00Y	96(4)	65(3)	54(2)	15(2)	-29(2)	13(2)

Table S11 Bond Lengths for 4.

Atom	Atom	Length/Å	Atom	Atom	Length/Å
O001	C00B	1.462(4)	C00B	C00F	1.554(4)
O001	B00P	1.370(4)	C00B	C00O	1.517(5)
O002	C00I	1.462(4)	C00B	C00Q	1.517(4)
O002	B00R	1.369(4)	C00C	C00K	1.386(5)
O003	C00F	1.469(3)	C00D	C00E	1.381(5)
O003	B00P	1.369(4)	C00D	C00H	1.383(5)
O004	C00J	1.468(4)	C00D	C00X	1.510(5)
O004	B00R	1.358(4)	C00F	C00S	1.512(5)
N005	N006	1.416(3)	C00F	C00U	1.521(5)
N005	C008	1.419(4)	C00G	C00N	1.385(5)
N005	B00R	1.440(4)	C00I	C00J	1.555(4)
N006	C007	1.426(4)	C00I	C00L	1.523(5)
N006	B00P	1.431(4)	C00I	C00V	1.513(5)
C007	C00C	1.385(4)	C00J	C00T	1.511(5)
C007	C00G	1.385(4)	C00J	C00W	1.512(5)
C008	C009	1.399(4)	C00K	C00M	1.384(5)
C008	C00A	1.392(4)	C00M	C00N	1.381(5)
C009	C00E	1.389(4)	C00M	C00Y	1.506(5)
C00A	C00H	1.384(4)			

Table S12 Bond Angles for 4.

Atom	Atom	Atom	Angle/°	Atom	Atom	Atom	Angle/°
B00P	O001	C00B	106.4(2)	O003	C00F	C00S	108.9(3)
B00R	O002	C00I	106.2(2)	O003	C00F	C00U	106.1(3)
B00P	O003	C00F	106.1(2)	C00S	C00F	C00B	114.1(3)
B00R	O004	C00J	106.5(2)	C00S	C00F	C00U	111.3(3)
N006	N005	C008	116.3(2)	C00U	C00F	C00B	113.7(3)
N006	N005	B00R	116.0(2)	C00N	C00G	C007	120.3(3)
C008	N005	B00R	127.7(2)	C00D	C00H	C00A	122.1(3)
N005	N006	C007	116.7(2)	O002	C00I	C00J	102.7(2)
N005	N006	B00P	115.8(2)	O002	C00I	C00L	106.3(3)
C007	N006	B00P	126.0(2)	O002	C00I	C00V	108.4(3)
C00C	C007	N006	121.3(3)	C00L	C00I	C00J	112.8(3)
C00C	C007	C00G	118.5(3)	C00V	C00I	C00J	114.8(3)
C00G	C007	N006	120.2(3)	C00V	C00I	C00L	110.9(3)
C009	C008	N005	121.2(3)	O004	C00J	C00I	102.3(2)
C00A	C008	N005	121.0(3)	O004	C00J	C00T	106.5(3)
C00A	C008	C009	117.8(3)	O004	C00J	C00W	108.9(3)
C00E	C009	C008	120.3(3)	C00T	C00J	C00I	113.9(3)
C00H	C00A	C008	120.6(3)	C00T	C00J	C00W	110.6(3)
O001	C00B	C00F	102.7(2)	C00W	C00J	C00I	114.1(3)
O001	C00B	C00O	107.2(3)	C00M	C00K	C00C	121.7(3)
O001	C00B	C00Q	108.7(3)	C00K	C00M	C00Y	121.5(4)

C00O	C00B	C00F	113.6(3)	C00N	C00M	C00K	117.3(3)
C00Q	C00B	C00F	114.5(3)	C00N	C00M	C00Y	121.3(4)
C00Q	C00B	C00O	109.6(3)	C00M	C00N	C00G	121.8(3)
C007	C00C	C00K	120.3(3)	O001	B00P	N006	121.9(3)
C00E	C00D	C00H	117.1(3)	O003	B00P	O001	113.9(3)
C00E	C00D	C00X	121.8(3)	O003	B00P	N006	124.2(3)
C00H	C00D	C00X	121.0(3)	O002	B00R	N005	120.8(3)
C00D	C00E	C009	122.1(3)	O004	B00R	O002	114.5(3)
O003	C00F	C00B	101.8(2)	O004	B00R	N005	124.7(3)

Table 13 Torsion Angles for 4.

A	B	C	D	Angle/°	A	B	C	D	Angle/°
O001	C00B	C00F	O003	29.5(3)	C00H	C00D	C00E	C009	0.4(5)
O001	C00B	C00F	C00S	146.6(3)	C00I	O002	B00R	O004	-9.5(4)
O001	C00B	C00F	C00U	-84.2(3)	C00I	O002	B00R	N005	171.4(3)
O002	C00I	C00J	O004	-27.4(3)	C00J	O004	B00R	O002	-9.3(4)
O002	C00I	C00J	C00T	87.0(3)	C00J	O004	B00R	N005	169.8(3)
O002	C00I	C00J	C00W	-144.8(3)	C00K	C00M	C00N	C00G	1.4(6)
N005	N006	C007	C00C	-16.1(4)	C00L	C00I	C00J	O004	86.7(3)
N005	N006	C007	C00G	163.9(3)	C00L	C00I	C00J	C00T	-158.9(3)
N005	N006	B00P	O001	-4.8(4)	C00L	C00I	C00J	C00W	-30.7(4)
N005	N006	B00P	O003	175.3(3)	C00O	C00B	C00F	O003	-85.9(3)
N005	C008	C009	C00E	-178.9(3)	C00O	C00B	C00F	C00S	31.2(4)
N005	C008	C00A	C00H	179.2(3)	C00O	C00B	C00F	C00U	160.4(3)

N006 N005 C008 C009	1.1(4)	B00P O001 C00B C00F	-22.7(3)
N006 N005 C008 C00A	-179.4(3)	B00P O001 C00B C00O	97.2(3)
N006 N005 B00R O002	-15.1(4)	B00P O001 C00B C00Q	-144.4(3)
N006 N005 B00R O004	165.9(3)	B00P O003 C00F C00B	-26.1(3)
N006 C007 C00C C00K	-178.7(3)	B00P O003 C00F C00S	-146.9(3)
N006 C007 C00G C00N	178.6(3)	B00P O003 C00F C00U	93.1(3)
C007 N006 B00P O001	-170.3(3)	B00P N006 C007 C00C	149.3(3)
C007 N006 B00P O003	9.7(5)	B00P N006 C007 C00G	-30.6(4)
C007 C00C C00K C00M	0.2(5)	C00Q C00B C00F O003	147.2(3)
C007 C00G C00N C00M	0.0(6)	C00Q C00B C00F C00S	-95.7(4)
C008 N005 N006 C007	-79.6(3)	C00Q C00B C00F C00U	33.5(4)
C008 N005 N006 B00P	113.4(3)	B00R O002 C00I C00J	22.7(3)
C008 N005 B00R O002	167.3(3)	B00R O002 C00I C00L	-96.0(3)
C008 N005 B00R O004	-11.8(5)	B00R O002 C00I C00V	144.7(3)
C008 C009 C00E C00D	-1.2(5)	B00R O004 C00J C00I	22.6(3)
C008 C00A C00H C00D	0.6(5)	B00R O004 C00J C00T	-97.2(3)
C009 C008 C00A C00H	-1.4(5)	B00R O004 C00J C00W	143.6(3)
C00A C008 C009 C00E	1.6(5)	B00R N005 N006 C007	102.5(3)
C00B O001 B00P O003	6.9(3)	B00R N005 N006 B00P	-64.5(3)
C00B O001 B00P N006	-173.0(3)	B00R N005 C008 C009	178.8(3)
C00C C007 C00G C00N	-1.4(5)	B00R N005 C008 C00A	-1.8(5)
C00C C00K C00M C00N	-1.5(5)	C00V C00I C00J O004	-144.9(3)
C00C C00K C00M C00Y	179.3(4)	C00V C00I C00J C00T	-30.5(4)

C00E	C00D	C00H	C00A	-0.1(5)	C00V	C00I	C00J	C00W	97.7(4)
C00F	O003	B00P	O001	13.3(3)	C00X	C00D	C00E	C009	-179.3(3)
C00F	O003	B00P	N006	-166.8(3)	C00X	C00D	C00H	C00A	179.6(4)
C00G	C007	C00C	C00K	1.3(5)	C00Y	C00M	C00N	C00G	-179.4(4)

Table S14 Hydrogen Atom Coordinates (Å×10^4) and Isotropic Displacement Parameters (Å^2×10^3) for 4.

**Atom**	***x***	***y***	***z***	**U(eq)**
H009	-3753	-4489	-2906	42
H00A	-1638	-2273	-3856	40
H00C	-877	-5302	-3338	44
H00E	-4227	-3192	-2059	48
H00G	-3853	-7091	-3426	48
H00H	-2118	-1006	-2998	47
H00K	-79	-6562	-2560	53
H00B	-1904	-5281	-6299	74
H00D	-1269	-4246	-6649	74
H00F	-2276	-4069	-6072	74
H00N	-3037	-8344	-2651	57
H00I	-6229	-5063	-4623	67
H00J	-6829	-5191	-5380	67
H00L	-6169	-4078	-5177	67
H00M	-4989	-4211	-6225	74
H00O	-5505	-5373	-6460	74

H00P	-4121	-5222	-6352	74
H00Q	-5814	-7994	-5177	77
H00R	-6425	-7114	-5681	77
H00S	-6468	-6956	-4846	77
H00T	933	-2630	-4791	84
H00U	1466	-3440	-5371	84
H00V	886	-3925	-4670	84
H00W	-3243	-6683	-5763	80
H00X	-4291	-7167	-6230	80
H00Y	-3791	-7855	-5579	80
H00Z	844	-5372	-5426	81
H	757	-4961	-6224	81
HA	21	-6006	-5973	81
H00	-1237	-2428	-6096	80
HB	51	-2753	-6362	80
HC	-109	-1829	-5773	80
H0AA	-3994	-545	-2081	91
HD	-2782	-812	-1690	91
HE	-3945	-1481	-1493	91
H1AA	-82	-8427	-2086	107
HF	-1247	-9154	-2143	107
HG	-1144	-8230	-1549	107

Crystal structure data for **9**

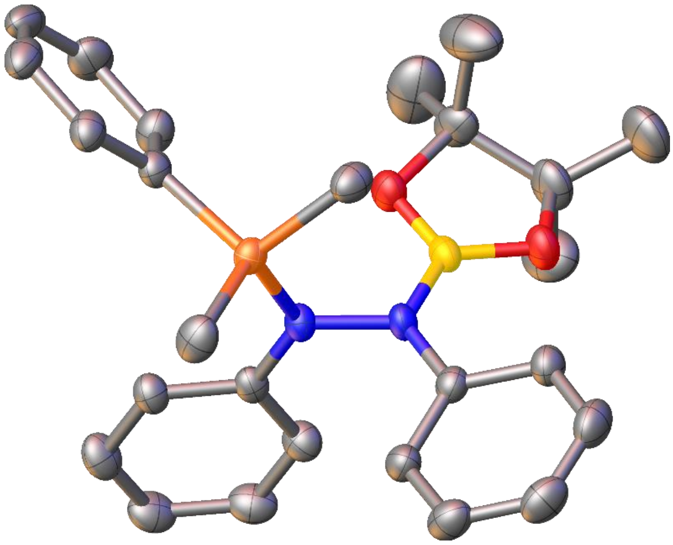

Table S15 Crystal data and structure refinement for 9.

Empirical formula	$C_{26}H_{33}BN_2O_2Si$
Formula weight	444.44
Temperature/K	173
Crystal system	monoclinic
Space group	$P2_1$
a/Å	8.51365(19)
b/Å	12.4441(3)
c/Å	11.7153(3)
α/°	90
β/°	91.067(2)
γ/°	90
Volume/Å^3	1240.96(5)

Z	2
ρ_{calc}g/cm^3	1.189
μ/mm^{-1}	1.019
F(000)	476.0
Crystal size/mm^3	0.15 × 0.15 × 0.075
Radiation	CuKα (λ = 1.54184)
2Θ range for data collection/°	7.548 to 142.434
Index ranges	-8 ≤ h ≤ 10, -9 ≤ k ≤ 15, -14 ≤ l ≤ 14
Reflections collected	7442
Independent reflections	3705 [R_{int} = 0.0367, R_{sigma} = 0.0437]
Data/restraints/parameters	3705/1/295
Goodness-of-fit on F^2	1.043
Final R indexes [I>=2σ (I)]	R_1 = 0.0574, wR_2 = 0.1581
Final R indexes [all data]	R_1 = 0.0634, wR_2 = 0.1604
Largest diff. peak/hole / e Å^{-3}	0.64/-0.31
Flack parameter	-0.01(4)

Table S16 Fractional Atomic Coordinates (×10^4) and Equivalent Isotropic Displacement Parameters (Å^2×10^3) for 9. U_{eq} is defined as 1/3 of of the trace of the orthogonalised U_{IJ} tensor.

Atom	*x*	*y*	*z*	U(eq)
Si01	3706.9(10)	4459.8(8)	9065.1(7)	24.5(3)
O002	3205(3)	3624(3)	5077(2)	34.9(6)
O003	4720(3)	4885(3)	6013(2)	32.7(6)

N1	2359(3)	4266(3)	6970(2)	25.5(7)
N2	2713(3)	5008(3)	7855(2)	25.4(6)
C006	2068(4)	6032(3)	7735(3)	25.3(7)
C007	1055(4)	3579(3)	7156(3)	26.9(8)
C008	2291(4)	6799(4)	8586(3)	29.8(8)
C009	-84(4)	3868(4)	7939(3)	32.1(8)
C00A	5511(4)	5267(3)	9430(3)	28.0(8)
C00B	935(5)	2584(4)	6608(3)	31.9(8)
C00C	6307(5)	5854(4)	8619(3)	35.7(9)
C00D	1634(5)	7818(4)	8492(4)	36.1(9)
C00E	1167(4)	6327(4)	6763(3)	32.3(8)
C00F	6194(4)	5173(4)	10522(3)	32.7(8)
C00G	8395(4)	6236(4)	9962(4)	39.8(10)
C00H	517(5)	7345(4)	6670(4)	38.6(9)
C00I	-286(6)	1900(4)	6848(4)	42.7(10)
C00J	7736(5)	6340(4)	8886(4)	42.1(10)
C00K	7623(5)	5656(4)	10783(4)	38.9(10)
C00L	745(5)	8105(4)	7526(4)	38.9(9)
C00M	2396(5)	4411(5)	10324(3)	39.4(9)
C00N	4341(6)	3999(5)	4263(4)	45.5(11)
C00O	-1298(5)	3156(4)	8178(3)	39.6(10)
B1	3417(4)	4252(4)	6027(3)	25.9(9)
C00Q	-1396(5)	2165(4)	7647(4)	42.5(10)

C00R	5596(4)	4551(5)	5023(3)	40.8(10)
C00S	4362(5)	3090(4)	8650(4)	36.6(9)
C00T	3448(7)	4877(6)	3550(4)	59.8(15)
C00U	4772(8)	3130(6)	3476(5)	63.8(16)
C00V	6744(7)	3640(7)	5479(5)	71(2)
C00W	6520(9)	5431(7)	4562(6)	78(2)

Table S17 Anisotropic Displacement Parameters (Å^2×10^3) for 9. The Anisotropic displacement factor exponent takes the form: $-2\pi^2[h^2a^{*2}U_{11}+2hka^*b^*U_{12}+\ldots]$.

Atom	U_{11}	U_{22}	U_{33}	U_{23}	U_{13}	U_{12}
Si01	26.6(4)	23.5(5)	23.5(4)	1.1(4)	0.7(3)	-4.6(4)
O002	41.4(13)	36.8(17)	26.8(12)	-9.1(12)	6.8(10)	-3.7(13)
O003	35.2(12)	36.7(17)	26.4(12)	-6.2(11)	5.2(10)	-3.0(12)
N1	28.7(12)	23.0(18)	24.9(12)	-6.6(12)	2.7(10)	-2.2(12)
N2	30.3(13)	24.5(17)	21.3(12)	-2.6(12)	0.7(10)	-0.9(13)
C006	27.1(14)	23(2)	25.5(15)	-0.1(14)	5.8(12)	-3.9(14)
C007	27.5(14)	27(2)	26.2(15)	-2.1(14)	-3.2(13)	-0.9(16)
C008	35.2(17)	26(2)	28.9(16)	-1.2(15)	3.6(14)	-1.1(16)
C009	28.0(15)	38(2)	30.7(17)	-6.6(16)	-0.4(13)	-1.5(16)
C00A	30.9(16)	23(2)	30.1(16)	-3.2(14)	0.0(13)	-0.2(15)
C00B	34.7(17)	28(2)	33.0(18)	-4.9(16)	-1.0(15)	-1.4(16)
C00C	38.5(18)	37(2)	31.4(18)	-1.8(16)	1.2(15)	-7.6(18)
C00D	47(2)	22(2)	39(2)	0.8(17)	8.0(17)	-2.7(18)
C00E	36.6(17)	33(2)	27.2(17)	1.5(16)	1.8(14)	2.5(17)

C00F	37.6(18)	27(2)	33.4(18)	-1.8(16)	-4.5(15)	0.9(17)
C00G	25.5(15)	31(2)	63(3)	-11(2)	-3.4(17)	0.2(17)
C00H	37.4(18)	39(3)	39(2)	10.7(18)	2.7(16)	6(2)
C00I	46(2)	31(2)	50(2)	-4.3(19)	-10.3(18)	-7.1(19)
C00J	35.1(18)	42(3)	50(2)	-2(2)	10.6(17)	-9.6(19)
C00K	38.4(19)	31(2)	47(2)	-5.4(18)	-12.9(17)	4.2(18)
C00L	39.3(19)	27(2)	51(2)	8.5(19)	4.9(18)	3.5(18)
C00M	42.5(18)	49(3)	26.4(15)	1.8(18)	2.7(14)	-10(2)
C00N	54(2)	51(3)	31.6(19)	-9(2)	14.8(18)	-3(2)
C00O	30.8(17)	53(3)	34.5(18)	-0.3(18)	1.2(15)	-5.6(18)
B1	30.2(16)	27(2)	20.8(15)	-3.5(15)	0.5(13)	4.2(16)
C00Q	37.2(19)	45(3)	45(2)	8(2)	-4.2(18)	-13.3(19)
C00R	33.4(16)	62(3)	27.1(16)	-5(2)	10.1(14)	-4(2)
C00S	33.9(17)	30(2)	45(2)	2.6(17)	-6.2(16)	-2.6(16)
C00T	63(3)	78(4)	38(2)	9(2)	-5(2)	11(3)
C00U	72(3)	74(4)	46(2)	-20(3)	18(2)	5(3)
C00V	58(3)	107(6)	49(3)	-2(3)	-4(2)	29(3)
C00W	80(4)	95(6)	61(3)	-5(4)	23(3)	-32(4)

Table S18 Bond Lengths for 9.

Atom	Atom	Length/Å	Atom	Atom	Length/Å
Si01	N2	1.773(3)	C00A	C00C	1.386(6)
Si01	C00A	1.878(4)	C00A	C00F	1.400(5)
Si01	C00M	1.867(4)	C00B	C00I	1.377(6)

Si01	C00S	1.860(5)	C00C	C00J	1.389(6)
O002	C00N	1.448(5)	C00D	C00L	1.395(6)
O002	B1	1.369(5)	C00E	C00H	1.386(7)
O003	B1	1.361(5)	C00F	C00K	1.386(6)
O003	C00R	1.452(4)	C00G	C00J	1.376(7)
N1	N2	1.417(4)	C00G	C00K	1.379(7)
N1	C007	1.421(5)	C00H	C00L	1.390(7)
N1	B1	1.438(5)	C00I	C00Q	1.383(7)
N2	C006	1.394(5)	C00N	C00R	1.540(6)
C006	C008	1.391(5)	C00N	C00T	1.564(8)
C006	C00E	1.410(5)	C00N	C00U	1.472(7)
C007	C009	1.395(5)	C00O	C00Q	1.383(7)
C007	C00B	1.398(5)	C00R	C00V	1.583(8)
C008	C00D	1.390(6)	C00R	C00W	1.458(9)
C009	C00O	1.393(6)			

Table S19 Bond Angles for 9.

Atom	Atom	Atom	Angle/°	Atom	Atom	Atom	Angle/°
N2	Si01	C00A	110.64(16)	C008	C00D	C00L	120.6(4)
N2	Si01	C00M	111.12(17)	C00H	C00E	C006	120.8(4)
N2	Si01	C00S	106.52(17)	C00K	C00F	C00A	120.9(4)
C00M	Si01	C00A	109.62(18)	C00J	C00G	C00K	119.7(4)
C00S	Si01	C00A	107.55(17)	C00E	C00H	C00L	121.0(4)
C00S	Si01	C00M	111.3(2)	C00B	C00I	C00Q	121.3(5)

B1	O002	C00N	105.8(3)	C00G	C00J	C00C	120.4(4)
B1	O003	C00R	106.0(3)	C00G	C00K	C00F	120.1(4)
N2	N1	C007	115.8(3)	C00H	C00L	C00D	118.5(4)
N2	N1	B1	116.3(3)	O002	C00N	C00R	103.2(3)
C007	N1	B1	127.8(3)	O002	C00N	C00T	104.6(4)
N1	N2	Si01	115.3(2)	O002	C00N	C00U	110.7(5)
C006	N2	Si01	127.9(2)	C00R	C00N	C00T	108.8(4)
C006	N2	N1	116.3(3)	C00U	C00N	C00R	120.7(4)
N2	C006	C00E	121.9(3)	C00U	C00N	C00T	107.6(4)
C008	C006	N2	120.4(3)	C00Q	C00O	C009	121.0(4)
C008	C006	C00E	117.7(4)	O002	B1	N1	123.8(3)
C009	C007	N1	120.0(4)	O003	B1	O002	114.6(3)
C009	C007	C00B	119.0(4)	O003	B1	N1	121.6(3)
C00B	C007	N1	120.9(3)	C00I	C00Q	C00O	118.7(4)
C00D	C008	C006	121.3(4)	O003	C00R	C00N	103.2(3)
C00O	C009	C007	119.7(4)	O003	C00R	C00V	105.0(3)
C00C	C00A	Si01	122.3(3)	O003	C00R	C00W	111.7(5)
C00C	C00A	C00F	118.1(4)	C00N	C00R	C00V	107.0(5)
C00F	C00A	Si01	119.2(3)	C00W	C00R	C00N	119.6(4)
C00I	C00B	C007	120.2(4)	C00W	C00R	C00V	109.2(5)
C00A	C00C	C00J	120.8(4)				

Table S20 Hydrogen Atom Coordinates (Å×10^4) and Isotropic Displacement Parameters (Å^2×10^3) for 9.

Atom	*x*	*y*	*z*	U(eq)
H008	2904	6622	9246	36
H009	-33	4547	8309	39
H00B	1699	2378	6069	38
H00C	5870	5925	7871	43
H00D	1791	8325	9090	43
H00E	1005	5822	6164	39
H00F	5672	4772	11091	39
H00G	9378	6564	10139	48
H00H	-95	7525	6011	46
H00I	-368	1233	6456	51
H00J	8262	6745	8323	51
H00K	8071	5588	11528	47
H00L	306	8804	7456	47
H00A	2050	5141	10511	59
H00M	2971	4105	10979	59
H00N	1478	3963	10143	59
H00O	-2070	3355	8714	48
H00Q	-2211	1676	7828	51
H00P	3446	2657	8425	55
H00R	4905	2749	9300	55
H00S	5079	3143	8008	55

H00T	2510	4559	3188	90
H00U	4139	5158	2961	90
H00V	3134	5464	4055	90
H00W	5312	2558	3904	96
H00X	5471	3414	2893	96
H00Y	3821	2839	3108	96
H00Z	7188	3852	6223	107
H	7595	3538	4937	107
HA	6160	2967	5558	107
H00	5833	6048	4406	117
HB	7007	5200	3851	117
HC	7340	5638	5117	117

Crystal structure data for **11**

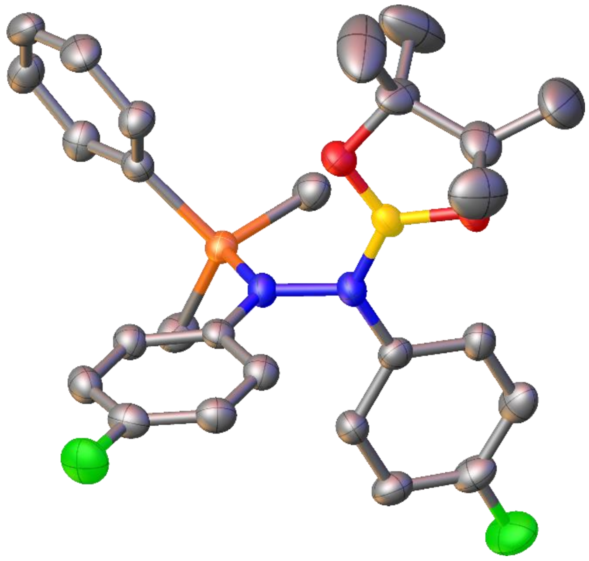

Table S21 Crystal data and structure refinement for 11.

Empirical formula	$C_{26}H_{31}BF_2N_2O_2Si$

Formula weight	480.43
Temperature/K	173
Crystal system	monoclinic
Space group	$P2_1$
a/Å	8.58378(17)
b/Å	12.6067(2)
c/Å	11.7191(2)
α/°	90
β/°	90.5690(18)
γ/°	90
Volume/Å^3	1268.10(4)
Z	2
ρ_{calc}g/cm^3	1.258
μ/mm^{-1}	1.154
F(000)	508.0
Crystal size/mm^3	0.4 × 0.3 × 0.25
Radiation	CuKα (λ = 1.54184)
2Θ range for data collection/°	7.544 to 142.394
Index ranges	$-8 \leq h \leq 10$, $-15 \leq k \leq 14$, $-14 \leq l \leq 12$
Reflections collected	7720
Independent reflections	4491 [R_{int} = 0.0345, R_{sigma} = 0.0509]
Data/restraints/parameters	4491/1/313
Goodness-of-fit on F^2	1.061

Final R indexes [I>=2σ (I)]	R_1 = 0.0635, wR_2 = 0.1735
Final R indexes [all data]	R_1 = 0.0716, wR_2 = 0.1806
Largest diff. peak/hole / e Å^{-3}	0.77/-0.39
Flack parameter	-0.01(3)

Table S22 Fractional Atomic Coordinates (×10^4) and Equivalent Isotropic Displacement Parameters (Å^2×10^3) for 11. U_{eq} is defined as 1/3 of of the trace of the orthogonalised U_{IJ} tensor.

Atom	*x*	*y*	*z*	U(eq)
Si01	6314.9(13)	5851.2(10)	912.9(9)	28.8(3)
F002	9733(4)	1286(3)	2653(3)	54.6(9)
O003	6749(5)	6756(3)	4860(3)	41.0(8)
O004	5278(4)	5478(3)	3968(3)	39.1(8)
F005	12559(4)	8715(3)	2000(4)	64.6(11)
N006	7631(4)	6058(3)	3004(3)	31.4(9)
N007	7287(4)	5324(3)	2128(3)	29.2(8)
C008	7917(5)	4306(4)	2271(4)	28.2(9)
C009	8804(6)	4039(4)	3241(4)	34.5(10)
C00A	8938(5)	6728(4)	2804(4)	31.5(9)
C00B	4527(5)	5049(4)	567(4)	30.9(9)
C00C	7708(6)	3534(4)	1427(4)	33.4(10)
C00D	10079(5)	6397(4)	2048(4)	34.7(10)
C00E	8320(6)	2519(4)	1555(5)	37.2(10)
C00F	9163(6)	2275(4)	2528(5)	37.9(11)

C00G	9418(6)	3018(5)	3368(5)	38.6(11)
C00H	1659(6)	4092(5)	89(5)	45.1(13)
C00I	3741(6)	4471(4)	1404(4)	38.3(11)
C00J	11307(6)	7079(5)	1774(5)	41.2(12)
C00K	9050(6)	7732(4)	3302(5)	37.0(11)
C00L	3842(6)	5132(4)	-510(5)	38.8(11)
C00M	11367(6)	8065(5)	2281(5)	43.7(12)
C00N	2326(7)	3996(5)	1153(5)	46.4(13)
C00O	10278(7)	8398(5)	3036(5)	44.2(12)
C00P	2409(6)	4655(4)	-742(5)	44.1(12)
C00Q	7642(6)	5906(6)	-341(4)	43.9(11)
B00R	6562(6)	6098(4)	3935(4)	29.8(11)
C00S	5664(6)	7201(4)	1321(5)	40.9(11)
C00T	4415(6)	5817(7)	4966(4)	52.8(15)
C00U	5604(8)	6410(6)	5682(5)	57.1(16)
C00V	6508(9)	5542(9)	6417(6)	78(3)
C00W	3444(13)	4994(10)	5419(8)	114(5)
C00X	3232(11)	6710(11)	4492(7)	97(4)
C00Y	5194(11)	7213(10)	6478(7)	91(3)

Table S23 Anisotropic Displacement Parameters ($Å^2×10^3$) for 11. The Anisotropic displacement factor exponent takes the form: $-2\pi^2[h^2a^{*2}U_{11}+2hka^*b^*U_{12}+…]$.

Atom	U_{11}	U_{22}	U_{33}	U_{23}	U_{13}	U_{12}
Si01	29.0(5)	30.5(6)	26.9(5)	0.6(5)	2.6(4)	-4.5(5)

F002	60(2)	35.5(16)	68(2)	8.6(16)	0.4(16)	10.4(16)
O003	47.3(19)	46(2)	29.8(17)	-11.6(16)	7.5(14)	-2.8(17)
O004	36.2(18)	48(2)	33.1(17)	-8.5(15)	6.6(13)	-0.9(15)
F005	49(2)	48(2)	96(3)	7(2)	10.4(19)	-16.4(17)
N006	33.3(18)	32(2)	29.2(17)	-7.5(15)	3.9(14)	-1.9(15)
N007	31.9(18)	30(2)	25.8(16)	-4.4(15)	2.3(13)	-3.7(16)
C008	25.6(19)	32(2)	27(2)	-1.2(18)	6.3(16)	-2.7(17)
C009	36(2)	38(3)	30(2)	0(2)	4.9(19)	-3(2)
C00A	27(2)	37(2)	30(2)	-0.2(19)	-2.0(16)	-1.4(19)
C00B	31(2)	29(2)	33(2)	-2.2(18)	2.9(18)	0.6(17)
C00C	34(2)	33(2)	32(2)	-1.7(19)	3.0(18)	-2.4(19)
C00D	33(2)	35(2)	37(2)	-7(2)	2.4(18)	0(2)
C00E	40(2)	32(2)	40(2)	-4(2)	7(2)	-3(2)
C00F	36(2)	32(2)	45(3)	7(2)	7(2)	2(2)
C00G	35(2)	42(3)	39(3)	5(2)	2(2)	2(2)
C00H	29(2)	40(3)	66(3)	-11(3)	-2(2)	0(2)
C00I	38(2)	40(3)	36(2)	0(2)	2(2)	-8(2)
C00J	30(2)	48(3)	45(3)	1(2)	0(2)	-5(2)
C00K	37(2)	34(3)	40(3)	-5(2)	-3(2)	-1(2)
C00L	44(3)	34(2)	38(2)	3(2)	-4(2)	0(2)
C00M	34(2)	42(3)	55(3)	7(2)	-1(2)	-9(2)
C00N	40(3)	46(3)	54(3)	-4(3)	13(2)	-14(2)
C00O	43(3)	31(2)	58(3)	1(2)	-8(2)	-1(2)

C00P	42(3)	39(3)	52(3)	-6(2)	-12(2)	3(2)
C00Q	41(2)	61(3)	30(2)	3(3)	6.6(18)	-13(3)
B00R	32(2)	32(3)	25(2)	-2.6(18)	0.0(18)	6.4(19)
C00S	38(2)	35(2)	49(3)	0(2)	-2(2)	-3(2)
C00T	38(2)	92(5)	29(2)	-9(3)	12.4(19)	-7(3)
C00U	61(4)	69(4)	42(3)	-11(3)	21(3)	-14(3)
C00V	71(5)	114(8)	49(4)	11(4)	-8(3)	6(5)
C00W	121(8)	139(10)	82(6)	-43(6)	62(6)	-71(8)
C00X	76(5)	160(11)	56(4)	-13(5)	-3(4)	57(6)
C00Y	75(5)	132(9)	65(5)	-47(6)	21(4)	-12(6)

Table S24 Bond Lengths for 11

Atom	Atom	Length/Å	Atom	Atom	Length/Å
Si01	N007	1.773(4)	C00B	C00I	1.401(7)
Si01	C00B	1.878(5)	C00B	C00L	1.391(7)
Si01	C00Q	1.869(5)	C00C	C00E	1.391(7)
Si01	C00S	1.855(6)	C00D	C00J	1.400(7)
F002	C00F	1.348(6)	C00E	C00F	1.380(8)
O003	B00R	1.373(6)	C00F	C00G	1.374(8)
O003	C00U	1.450(7)	C00H	C00N	1.372(9)
O004	B00R	1.352(7)	C00H	C00P	1.372(9)
O004	C00T	1.455(6)	C00I	C00N	1.383(7)
F005	C00M	1.355(6)	C00J	C00M	1.379(8)
N006	N007	1.412(5)	C00K	C00O	1.386(8)

N006	C00A	1.426(6)	C00L	C00P	1.394(8)
N006	B00R	1.433(6)	C00M	C00O	1.360(9)
N007	C008	1.402(6)	C00T	C00U	1.513(9)
C008	C009	1.403(7)	C00T	C00W	1.436(12)
C008	C00C	1.397(7)	C00T	C00X	1.611(12)
C009	C00G	1.397(7)	C00U	C00V	1.591(12)
C00A	C00D	1.392(7)	C00U	C00Y	1.423(11)
C00A	C00K	1.396(7)			

Table 25 Bond Angles for 11.

Atom	Atom	Atom	Angle/˚	Atom	Atom	Atom	Angle/˚
N007	Si01	C00B	110.4(2)	C00G	C00F	C00E	121.2(5)
N007	Si01	C00Q	111.1(2)	C00F	C00G	C009	119.6(5)
N007	Si01	C00S	106.1(2)	C00P	C00H	C00N	119.8(5)
C00Q	Si01	C00B	110.7(2)	C00N	C00I	C00B	120.4(5)
C00S	Si01	C00B	107.6(2)	C00M	C00J	C00D	118.7(5)
C00S	Si01	C00Q	110.9(3)	C00O	C00K	C00A	120.3(5)
B00R	O003	C00U	105.6(4)	C00B	C00L	C00P	120.6(5)
B00R	O004	C00T	106.0(4)	F005	C00M	C00J	117.8(5)
N007	N006	C00A	115.4(4)	F005	C00M	C00O	119.8(6)
N007	N006	B00R	116.5(4)	C00O	C00M	C00J	122.4(5)
C00A	N006	B00R	128.0(4)	C00H	C00N	C00I	120.8(5)
N006	N007	Si01	115.6(3)	C00M	C00O	C00K	119.2(5)
C008	N007	Si01	128.1(3)	C00H	C00P	C00L	120.3(5)

C008	N007	N006	115.8(4)	O003	B00R	N006	123.5(4)
N007	C008	C009	121.4(4)	O004	B00R	O003	114.5(4)
C00C	C008	N007	120.4(4)	O004	B00R	N006	122.0(4)
C00C	C008	C009	118.1(5)	O004	C00T	C00U	104.2(4)
C00G	C009	C008	120.5(5)	O004	C00T	C00X	104.6(5)
C00D	C00A	N006	119.1(4)	C00U	C00T	C00X	105.4(7)
C00D	C00A	C00K	119.5(5)	C00W	C00T	O004	112.8(7)
C00K	C00A	N006	121.3(4)	C00W	C00T	C00U	122.9(6)
C00I	C00B	Si01	121.8(4)	C00W	C00T	C00X	105.5(8)
C00L	C00B	Si01	119.5(4)	O003	C00U	C00T	103.8(5)
C00L	C00B	C00I	118.1(5)	O003	C00U	C00V	103.7(6)
C00E	C00C	C008	121.2(5)	C00T	C00U	C00V	106.5(7)
C00A	C00D	C00J	119.9(5)	C00Y	C00U	O003	113.3(7)
C00F	C00E	C00C	119.3(5)	C00Y	C00U	C00T	123.1(7)
F002	C00F	C00E	118.9(5)	C00Y	C00U	C00V	104.9(7)
F002	C00F	C00G	119.9(5)				

Table S26 Hydrogen Atom Coordinates (Å×10^4) and Isotropic Displacement Parameters (Å^2×10^3) for 11.

Atom	*x*	*y*	*z*	U(eq)
H009	8988	4555	3817	41
H00C	7136	3706	754	40
H00D	10025	5709	1719	42
H00E	8160	2000	978	45

H00G	10008	2840	4030	46
H00H	681	3769	-71	54
H00I	4181	4405	2147	46
H00J	12084	6866	1249	49
H00K	8280	7959	3825	44
H00L	4355	5518	-1092	47
H00N	1809	3599	1725	56
H00O	10358	9079	3378	53
H00P	1950	4720	-1480	53
H00A	8524	6374	-166	66
H00B	7070	6180	-1006	66
H00F	8029	5191	-508	66
H00M	4967	7153	1976	61
H00Q	5109	7529	677	61
H00R	6573	7634	1526	61
H00S	6920	4994	5910	117
H00T	5791	5218	6962	117
H00U	7371	5880	6834	117
H00V	2611	4829	4870	170
H00W	2988	5233	6137	170
H00X	4073	4357	5559	170
H00Y	3761	7399	4480	146
H	2324	6751	4991	146

HA	2890	6524	3717	146
H00Z	6133	7461	6879	136
HB	4460	6923	7032	136
HC	4707	7808	6072	136

References:

[1] C. Zhang, N. Jiao, *Angew. Chem. Int. Ed.* **2010**, *49*, 6174-6177

[2] M. B. Ansell, V. H. Menezes da Silva, G. Heerdt, A. A. C. Braga, J. Spencer, O. Navarro, *Catal. Sci. Technol.* **2016**, DOI: 10.1039/C6CY01266C

[3] P. S. Engel, W. -X. Wu, *J. Am. Chem. Soc.* **1989**, *111*, 1830-1835

[4] G. M. Sheldrick, *Acta Cryst.* **2015**, *A71*, 3-8

[5] G. M. Sheldrick, *Acta Cryst.* **2008**, *A64*, 112-122

[6] R. H. Blessing, *Acta Crystallogr. A. Found. Crystallogr.* **1995**, *51*, 33-38

[7] G. M. Sheldrick, *J. Appl. Cryst.* **2011**, *44*, 1281-1284

[8] O. V. Dolomanov, L. J. Bourhis, R. J. Gildea, J. A. K. Howard, H. Puschmann, *J. Appl. Cryst.* **2009**, *42*, 339-341
